# Improved pathogenicity prediction for rare human missense variants

**DOI:** 10.1016/j.ajhg.2021.11.010

**Published:** 2021-12-02

**Authors:** Yingzhou Wu, Hanqing Liu, Roujia Li, Song Sun, Jochen Weile, Frederick P. Roth

(The American Journal of Human Genetics *108*, 1891–1906; October 7, 2021)

In the originally published version of this article, Hanqing Liu was omitted from the author list. The author list has now been corrected online and includes Hanqing Liu and his current affiliations. The authors regret the error.

