# Supplementary material for: Improved pathogenicity prediction for rare human missense variants
Source: Am J Hum Genet. 2021 Sep 21;108(10):1891–906. doi: 10.1016/j.ajhg.2021.08.012 (PMC8546039; doi:10.1016/j.ajhg.2021.08.012)
Supplement: Document S1. Figures S1–S16 and Tables S1–S15 [file mmc1.pdf]

The American Journal of Human Genetics, Volume *108*

Supplemental information

Improved pathogenicity prediction for rare human missense variants

Yingzhou Wu, Roujia Li, Song Sun, Jochen Weile, and Frederick P. Roth

Supplemental Figures

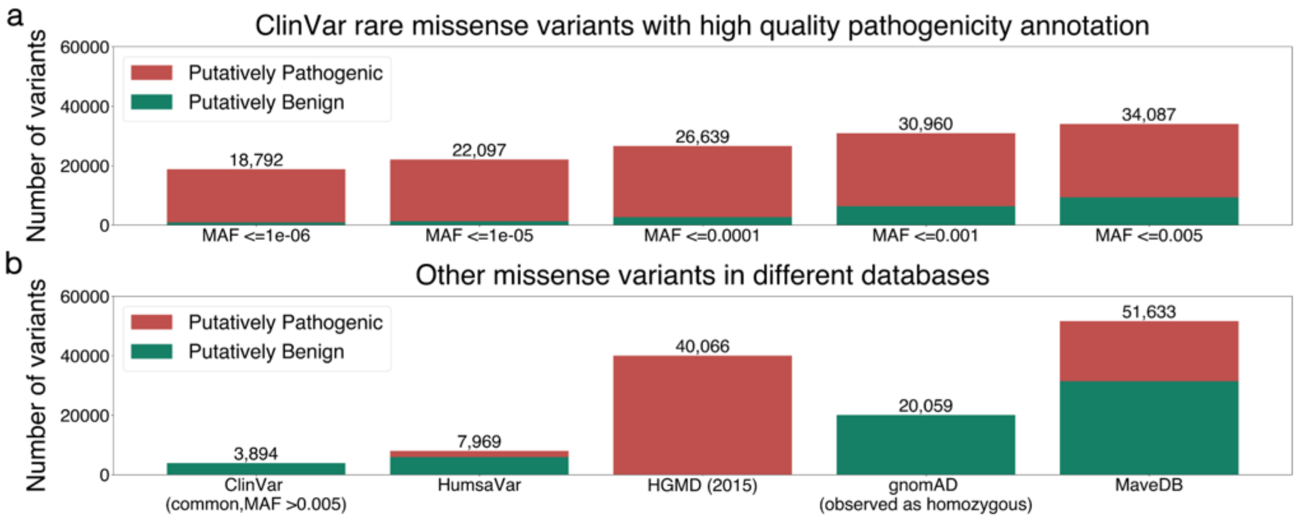

Figure S1: (a) The number of annotated variants from the high-quality ClinVAR[1] database with minor allele frequency (MAF) below the specified thresholds. (b) Other sources of variant training examples with less certain accuracy and representativity.

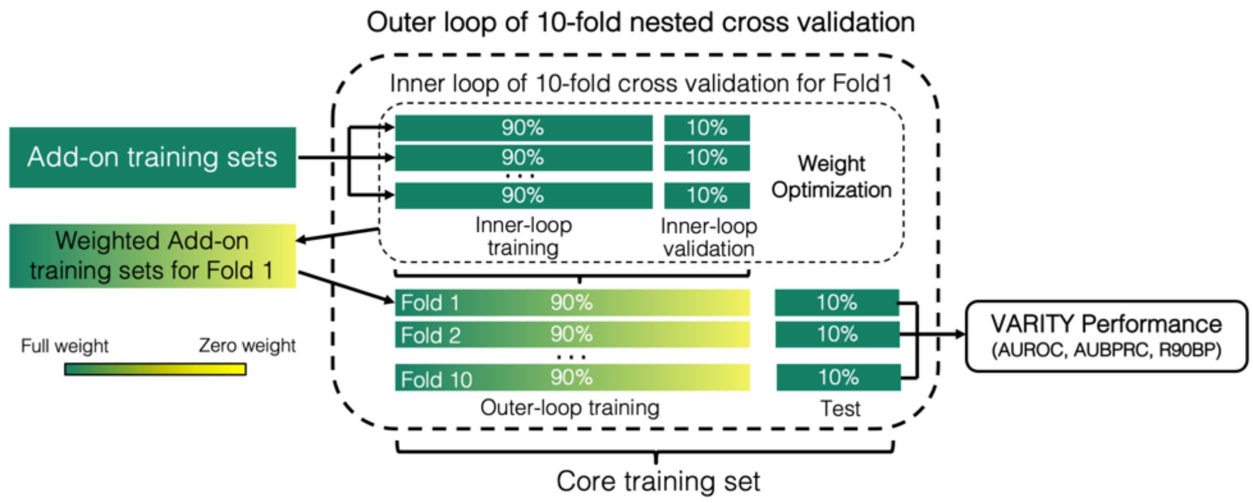

Figure S2: 10-fold nested cross validation. To measure VARITY performance, we used 10-fold nested cross validation, with the first fold of the outer loop ("Fold1") shown here for illustration. In each outer-loop, training sets (as weighted according to hyperparameters learned in the inner loop) were used to train VARITY models and test them using held-out test data. Three metrics—AUROC, AUBPRC and R90BP—were used for performance evaluation (see Methods).

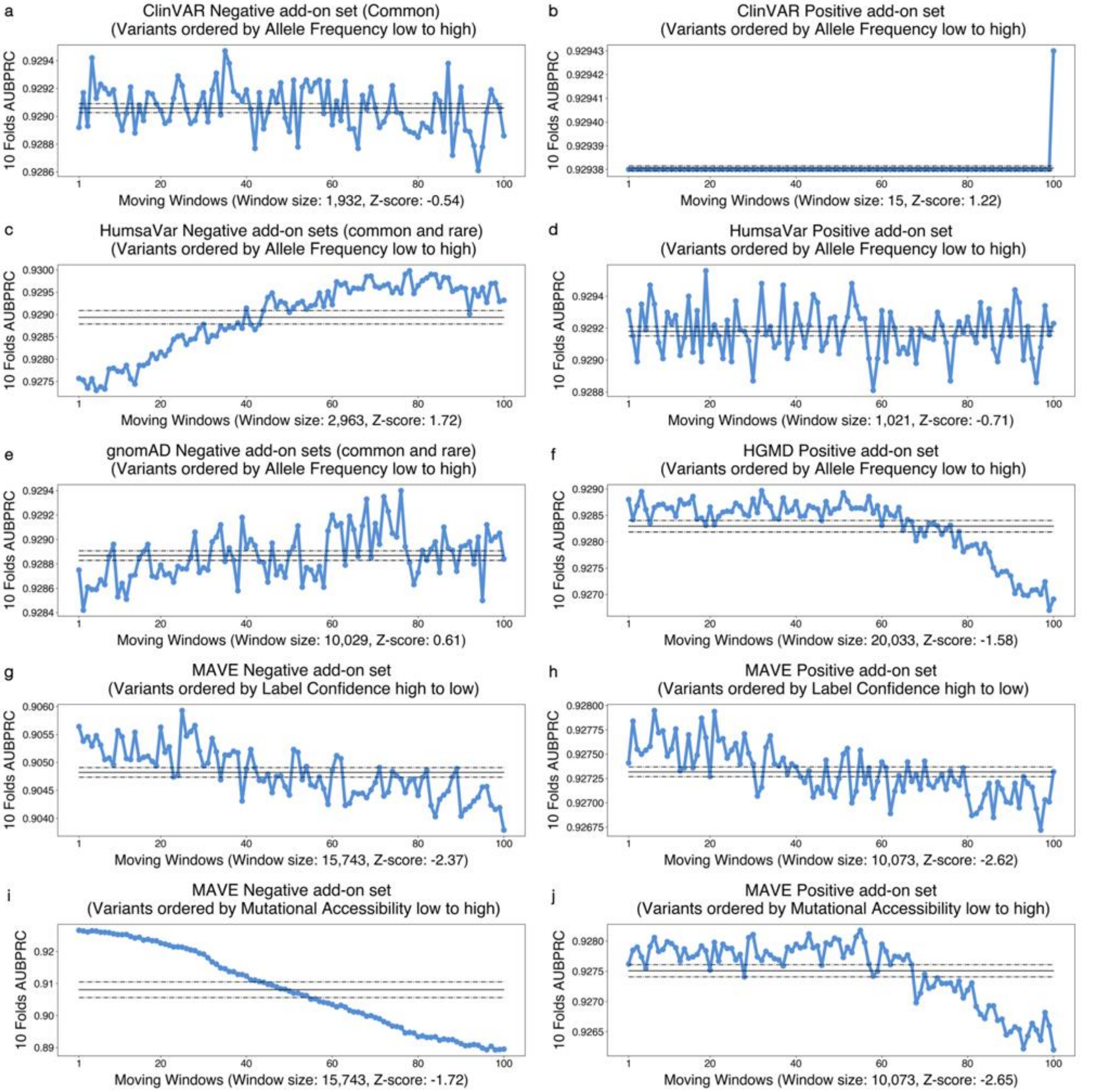

Figure S3: Moving window analysis for VARITY_R. Each panel illustrates assessment of a candidate informative property as a basis for weighting a single add-on set or combination of add-on sets. For Negative variants from HumsaVar[2] and gnomAD[3] (**Panel c and e**), the common (MAF>0.5%) and rare (MAF < 0.5%) add-on sets were combined for moving window analysis. Variants in add-on set(s) were ordered by the candidate informative property and 100 moving windows, each capturing same number of examples, were examined. To evaluate the predictive utility of each window, the model performance on the core set was estimated using 10-fold cross validation where the training examples in each fold was supplemented by examples in that moving window. Solid and dashed black lines indicate the mean ± standard error of VARITY_R performance measures over all moving windows. A Z-score was calculated to estimate direction and significance of the observed trend (see Methods).

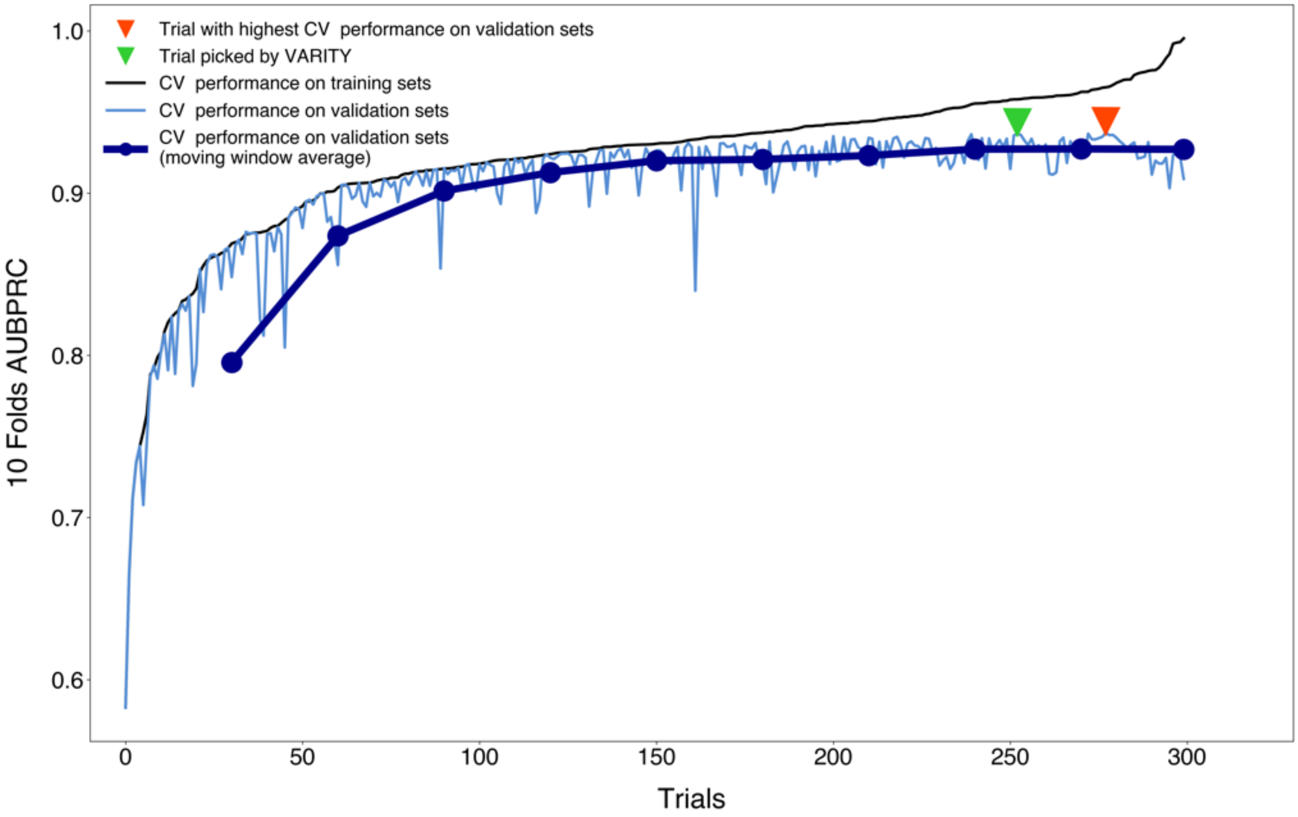

Figure S4: Hyperparameter tuning for the VARITY_R model. To optimize hyperparameters, 300 trials were performed using HyperOpt[4-5], each using a set of hyperparameter values suggested from previous trials (see Methods). To avoid overfitting in hyperparameter optimization, we did not simply choose hyperparameters showing the best numeric performance on validation sets (red triangle). Rather, the best hyperparameters (green triangle) were chosen as follows: 1) all trials were re-ordered from low to high AUBPRC (averaged over 10 training sets; black curve), and the average AUBPRC value from 10 validation sets was also shown (light blue curve); 2) a moving window (30 trials per window) was calculated for the cross-validation performance on validation sets (dark blue curve); 3) the 'fittest' moving window, i.e., the point at which the moving window performance starts to descend due to overfitting, was identified; and 4) Select the trial that performed best in cross-validation from within the fittest moving window (green triangle).

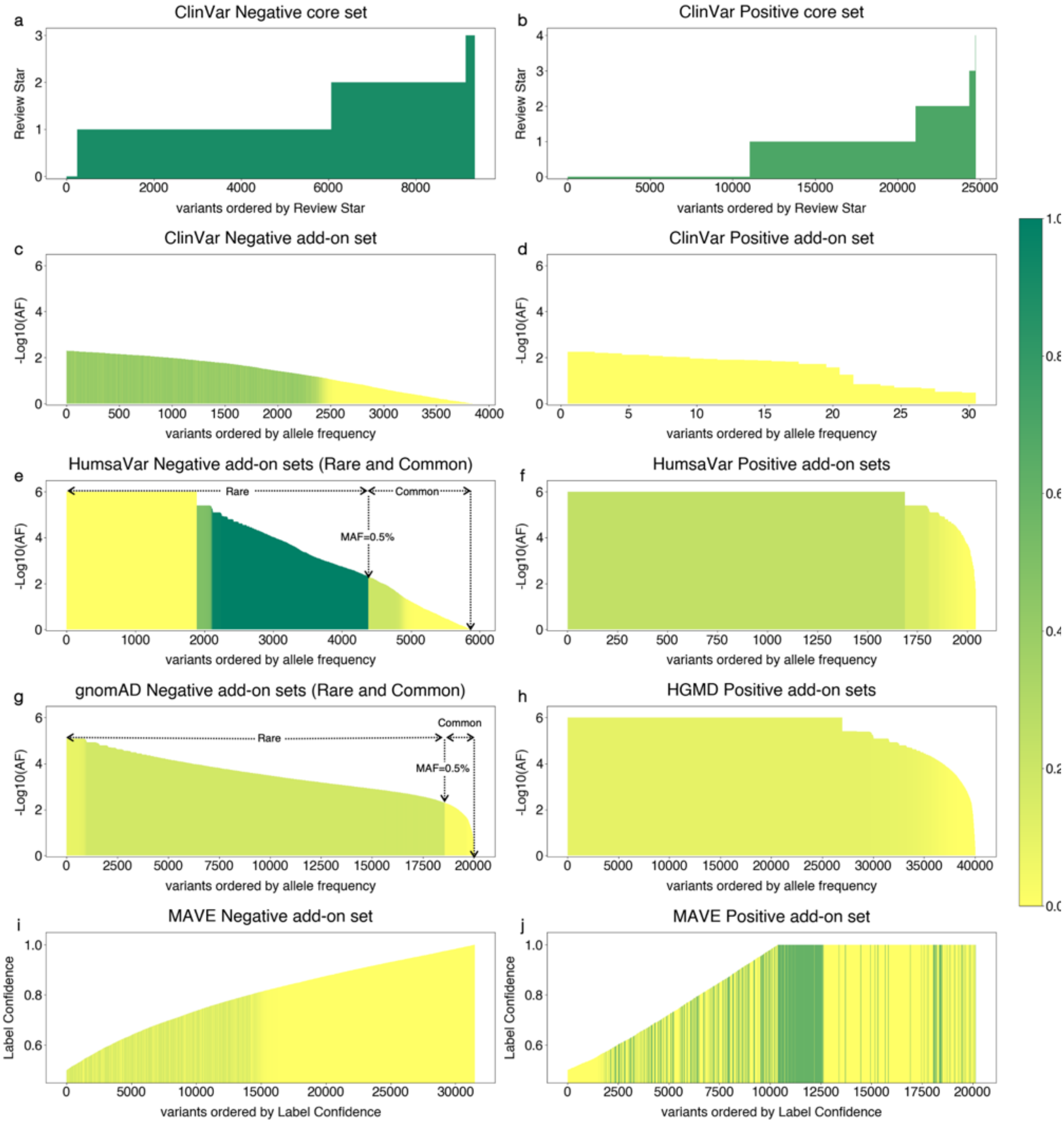

Figure S5: Weighted training sets for VARITY_R. Each plot illustrates the optimized weight with a color that varies between 0 (yellow; lowest weight) and 1 (green; highest weight) for a single core/add-on set or multiple core or add-on sets. For compactness, weighting for common and rare add-on sets of Negative training examples are shown together for HumsaVar[2] and gnomAD[3] (Panels **e** and **g**, respectively), while other Panels correspond to single core or add-on sets. For each plot, the x-axis indicates the rank of each variant ordered by the associated informative property (y-axis). Discontinuities in weight along the x-axis are possible where variant weights were based on multiple quality-informative properties (in which case the overall variant weight is the product of individual weights; see Methods and Table S3).

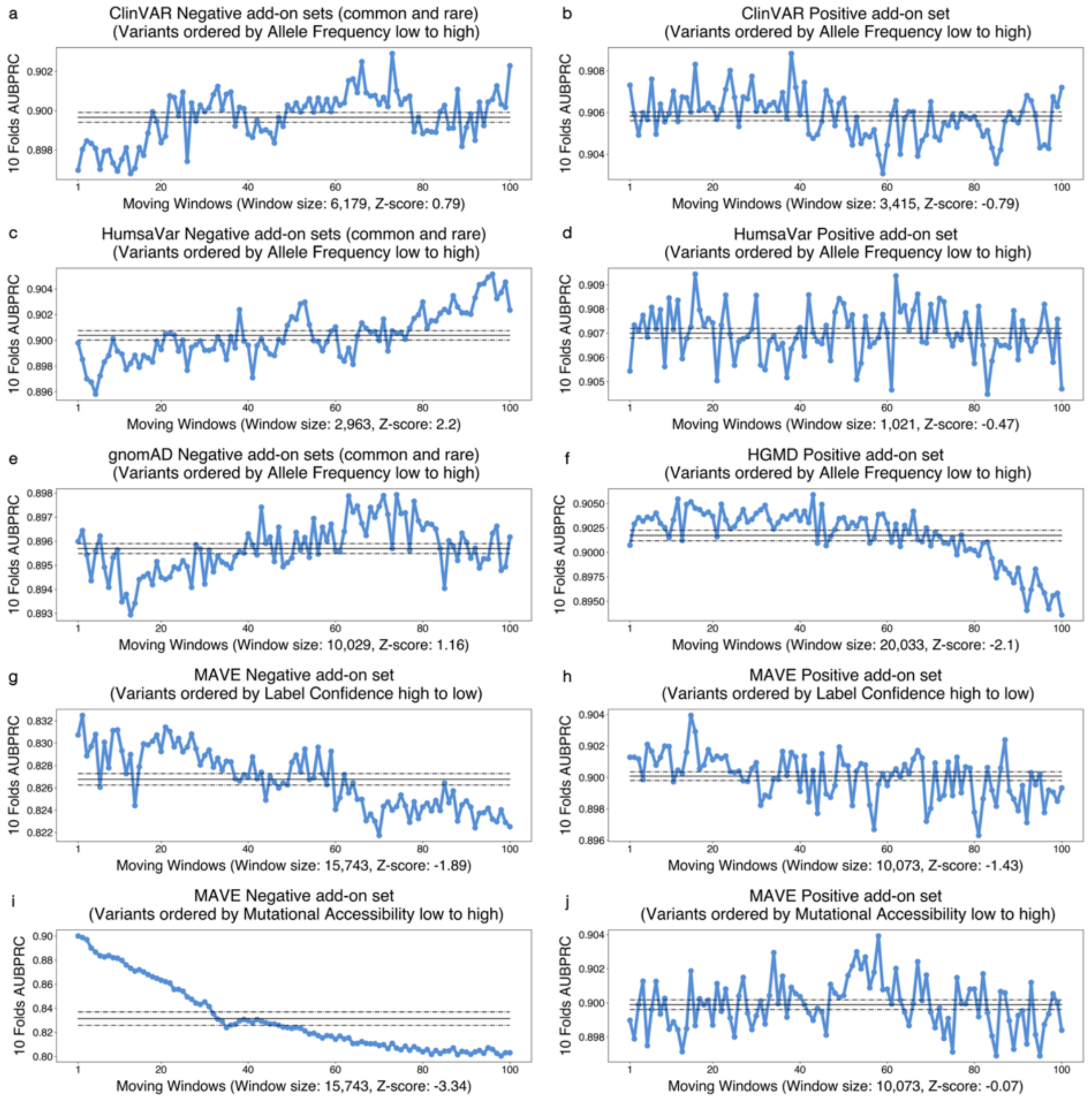

Figure S6: Moving window analysis for VARITY_ER. Each panel illustrates assessment of a candidate informative property as a basis for weighting a single add-on set or combination of add-on sets. For Negative variants from HumsaVar[2] and gnomAD[3] (**Panel c and e**), the common (MAF>=0.5%) and rare (MAF < 0.5%) add-on sets were combined for moving window analysis. Variants in add-on set(s) were ordered by the candidate informative property and 100 moving windows, each capturing same number of examples, were examined. To evaluate the predictive utility of each window, the model performance on the core set was estimated using 10-fold cross validation where the training examples in each fold was supplemented by examples in that moving window. Solid and dashed black lines indicate the mean ± standard error of VARITY_R performance measures over all moving windows. A Z-score was calculated to estimate direction and significance of the observed trend (see Methods).

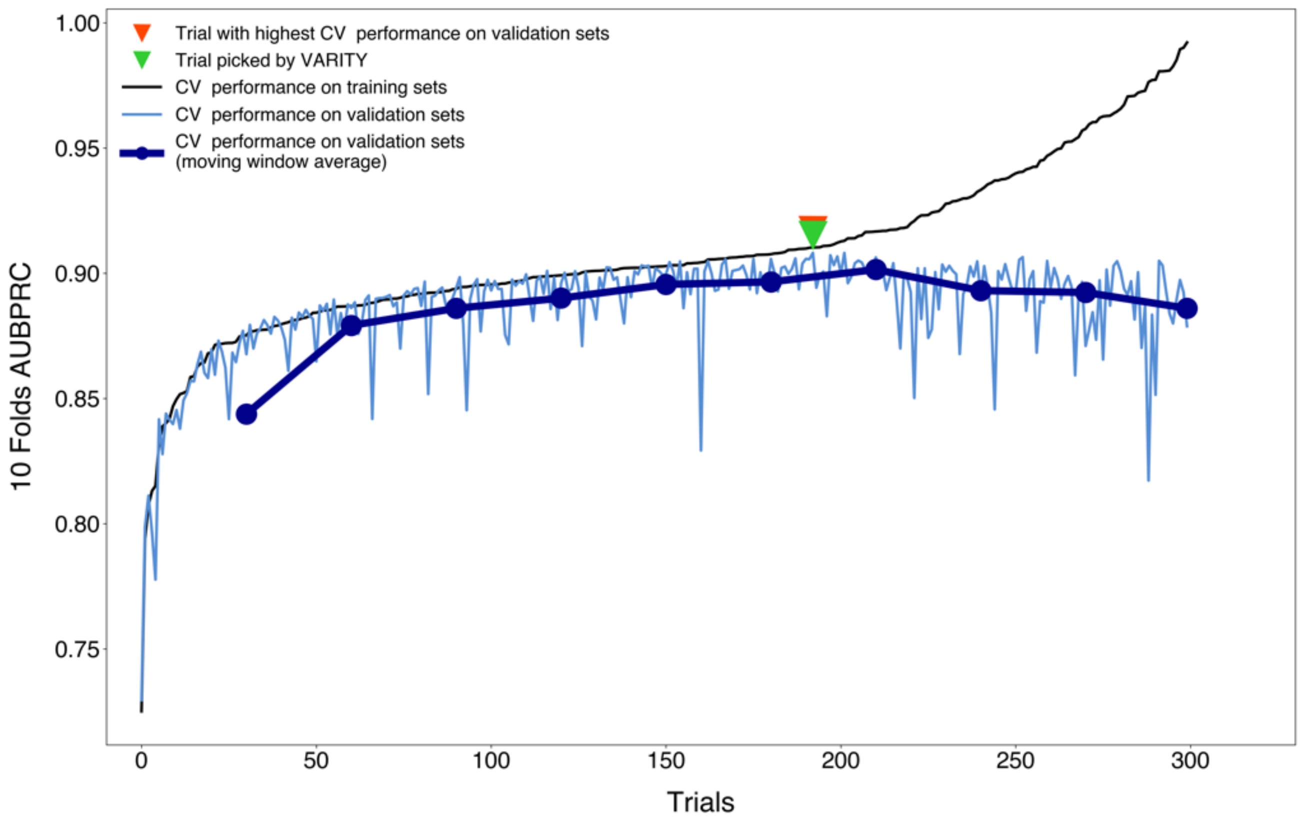

Figure S7: Hyperparameter tuning for the VARITY_ER model. To optimize hyperparameters, 300 trials were performed using HyperOpt[45], each using a set of hyperparameter values suggested from previous trials (see Methods). To avoid overfitting in hyperparameter optimization, we did not simply choose hyperparameters showing the best numeric performance on validation sets (red triangle). Rather, the best hyperparameters (green triangle) were chosen as follows: 1) all trials were re-ordered from low to high AUBPRC (averaged over 10 training sets; black curve), and the average AUBPRC value from 10 validation sets was also shown (light blue curve); 2) a moving window (30 trials per window) was calculated for the cross-validation performance on validation sets (dark blue curve); 3) the 'fittest' moving window, i.e., the point at which the moving window performance starts to descend due to overfitting, was identified; and 4) Select the trial that performed best in cross-validation from within the fittest moving window (green triangle).

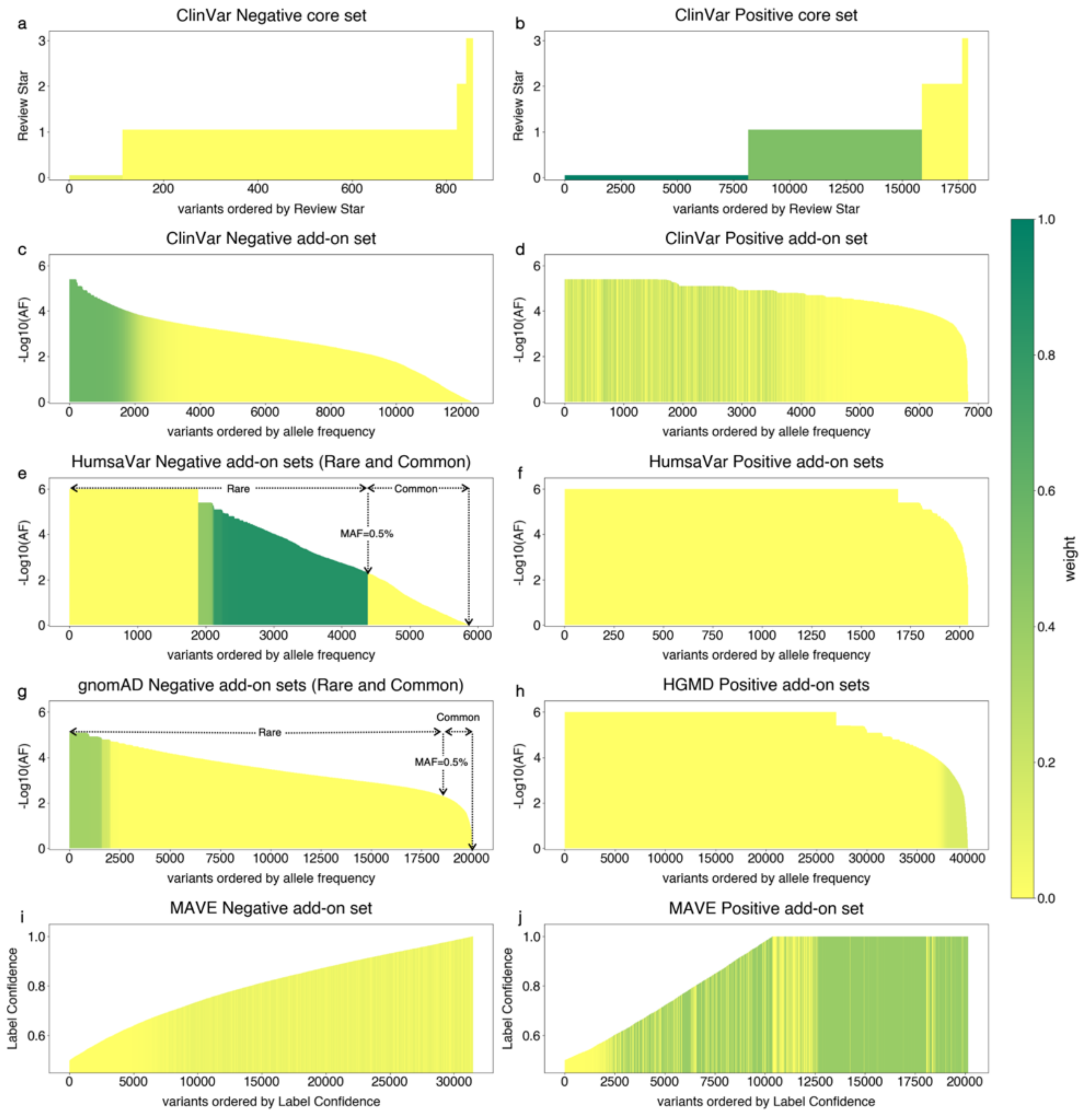

Figure S8: Weighted training sets for VARITY_ER. Each plot illustrates the optimized weight with a color that varies between 0 (yellow; lowest weight) and 1 (green; highest weight) for a single core/add-on set or multiple core or add-on sets. For compactness, weighting for common and rare add-on sets of Negative training examples are shown together for HumsaVar[2] and gnomAD[3] (Panels **e** and **g**, respectively), while other Panels correspond to single core or add-on sets. For each plot, the x-axis indicates the rank of each variant ordered by the associated informative property (y-axis). Discontinuities in weight along the x-axis are possible where variant weights were based on multiple quality-informative properties (in which case the overall variant weight is the product of individual weights; see Methods and Table S3).

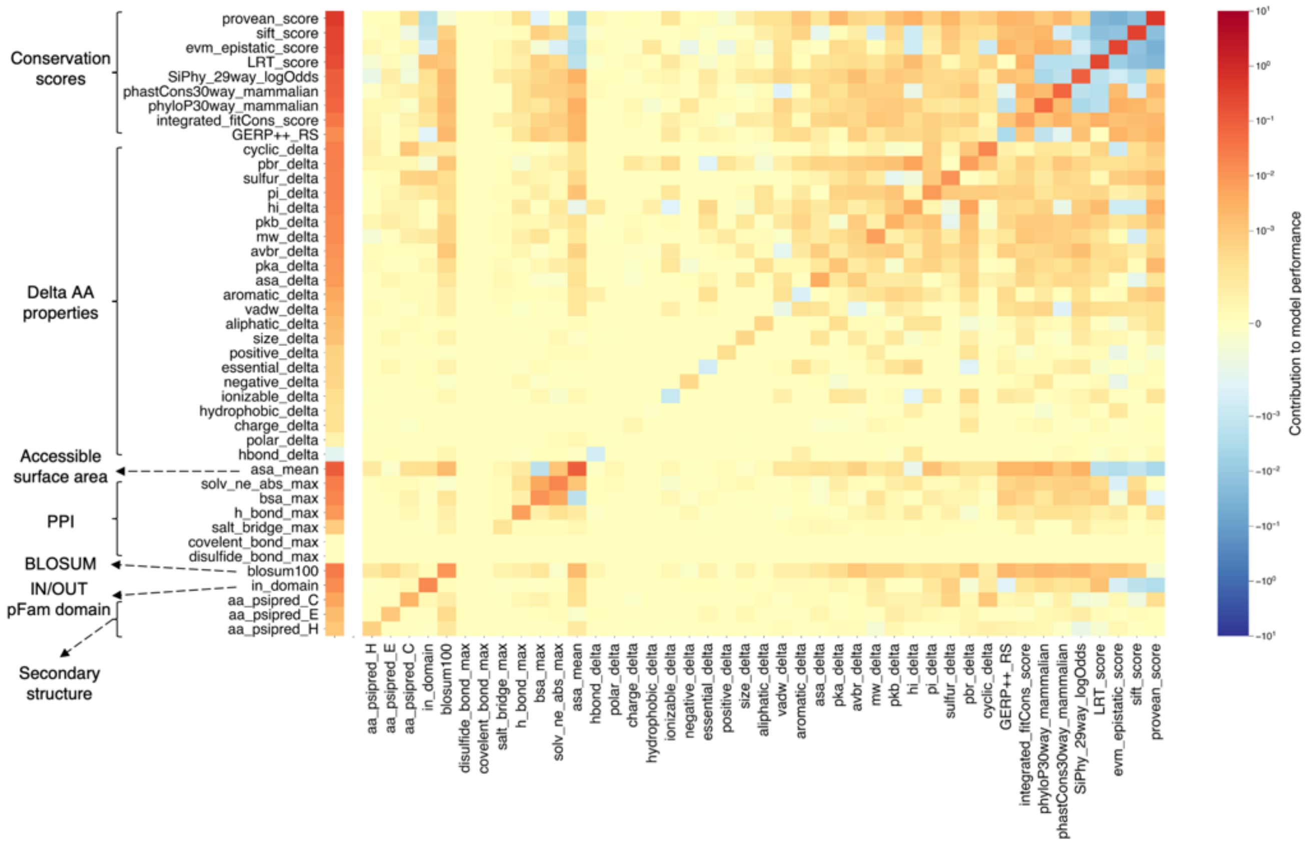

Figure S9: Individual feature contributions to VARITY_R model performance. The contribution of each feature to VARITY_R model performance was combined by weighted averaging across all training examples, using the weight of each training example that was optimized during hyperparameter tuning. The first column (left) indicates the total contribution to model performance of each feature, which consists of a feature-independent contribution (matrix cell on the diagonal on the corresponding row) and pair-wise differential feature contributions (non-diagonal matrix cells on the corresponding row). Red and blue color indicates positive and negative contribution to model performance respectively. A blue colored cell for pair-wise differential contribution indicates there is some redundancy between two features (e.g., between different conservation scores). The description of each feature can be found in Table S1.

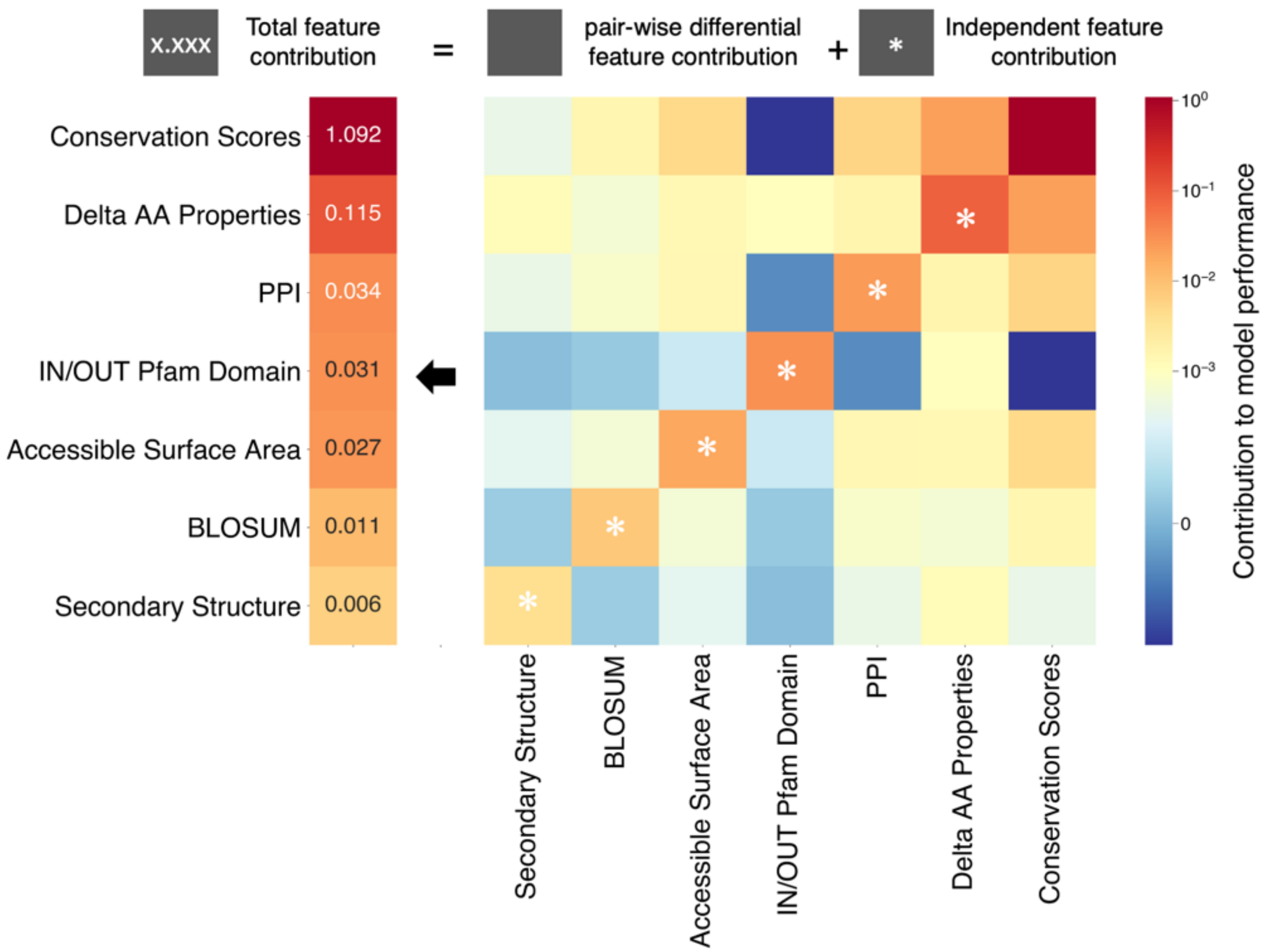

Figure S10: Feature group contributions to VARITY_ER model performance. The contribution of each feature group to VARITY_R model performance was averaged (weighted) across all training examples using the weight of each training example as optimized during hyperparameter tuning. The first column (left) indicates the total contribution to model performance of each feature group. For each feature group, the total contribution can be decomposed into the individual feature contribution (matrix cell with a star symbol on the corresponding row) and the differential contribution of that feature when it is combined with each other feature group (matrix cells without a star symbol on the corresponding row). Red and blue color indicates positive and negative contribution to model performance respectively. A blue colored cell for pair-wise differential feature contribution indicates there is a certain amount of redundancy between two feature groups (e.g., between Conservation Scores and IN/OUT Pfam domain).

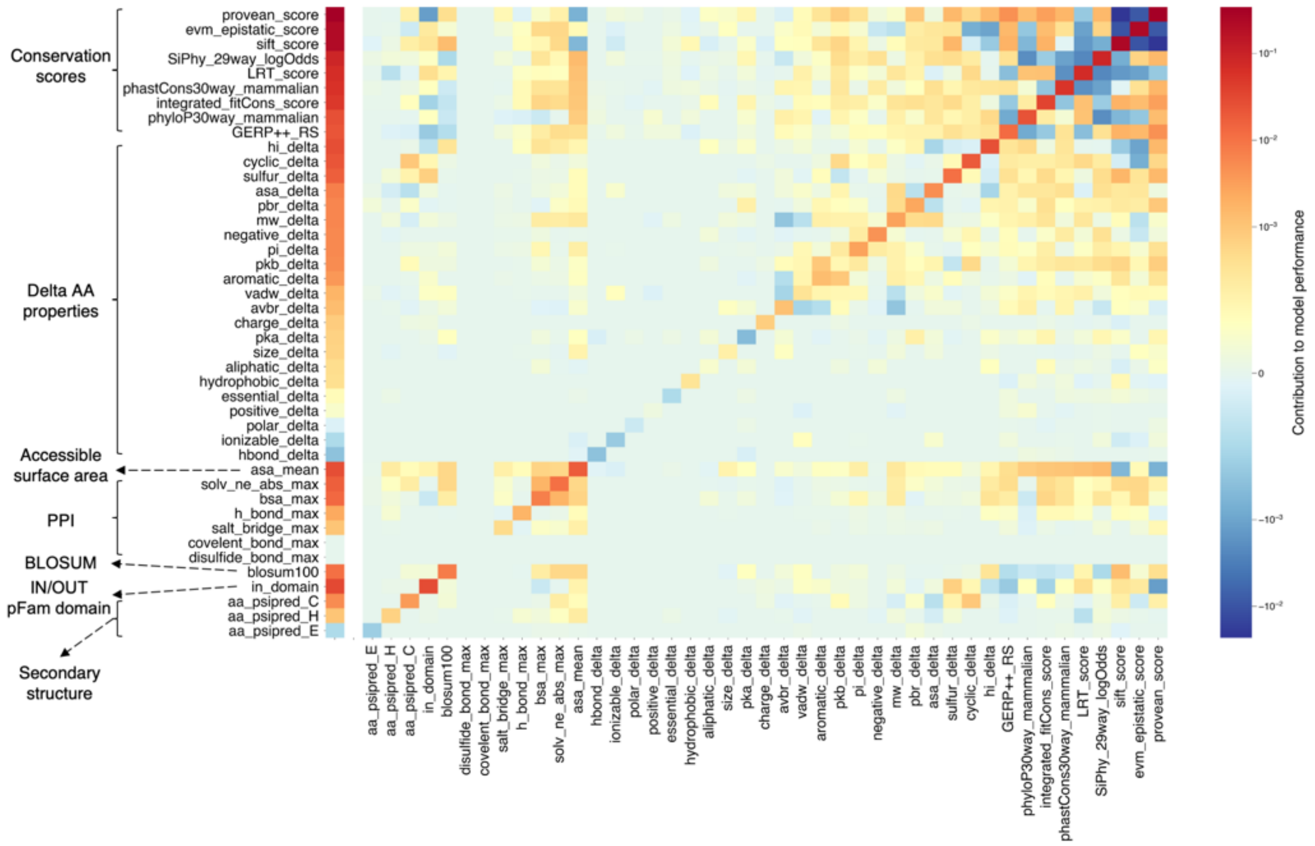

Figure S11: Individual feature contributions to VARITY_ER model performance. The contribution of each feature to VARITY_R model performance was combined by weighted averaging across all training examples, using the weight of each training example that was optimized during hyperparameter tuning. The first column (left) indicates the total contribution to model performance of each feature, which consists of a feature-independent contribution (matrix cell on the diagonal on the corresponding row) and pair-wise differential feature contributions (non-diagonal matrix cells on the corresponding row). Red and blue color indicates positive and negative contribution to model performance respectively. A blue colored cell for pair-wise differential contribution indicates there is some redundancy between two features (e.g., between different conservation scores). The description of each feature can be found in Table S1.

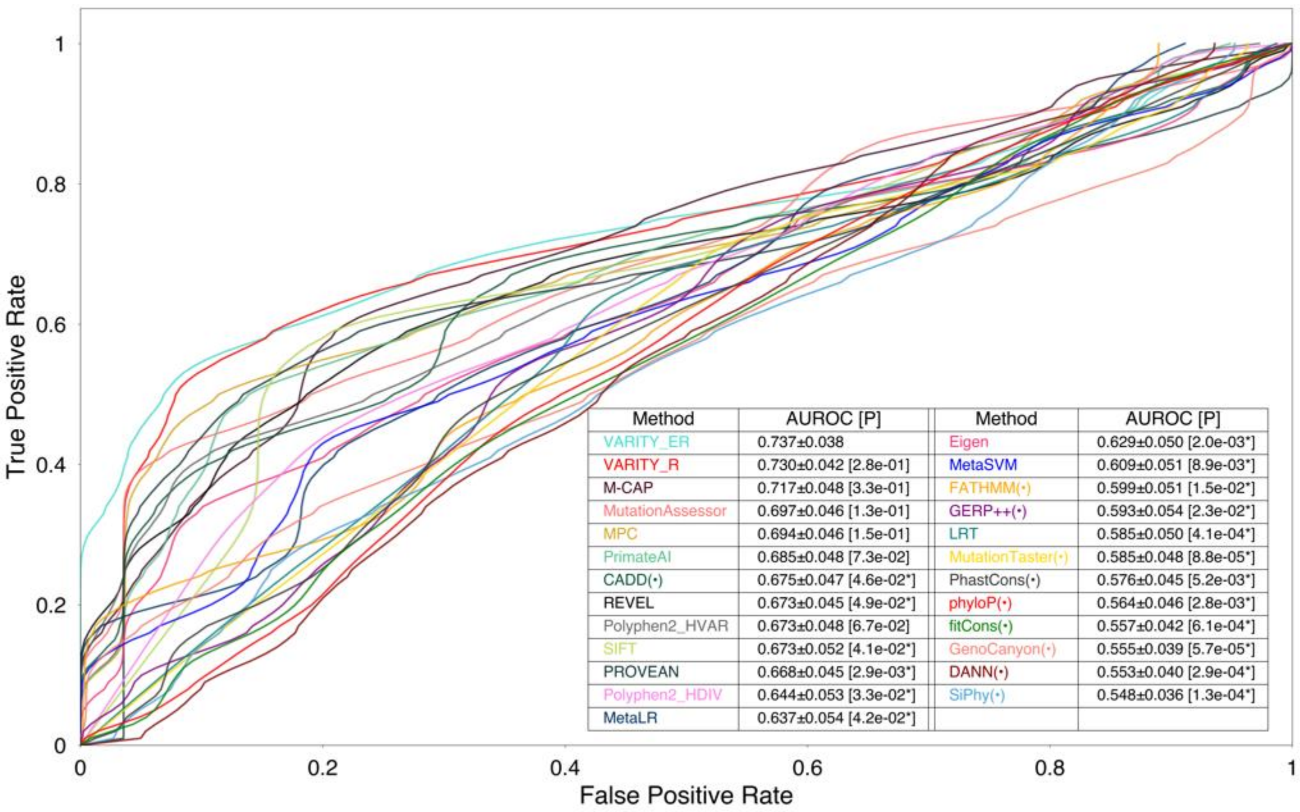

Figure S12: Assessing VARITY ROC performance for *de novo* variants observed in neurodevelopmental disorder studies. Here we show balanced ROC curves for VARITY_ER, VARITY_R and 23 other variant pathogenicity predictors, using a test set of 215 rare *de novo* variants (188 positive and 27 negative examples, see Methods). Recall was averaged over 2,000 bootstrapped test sets with standard error indicated by the surrounding grey region. As overall performance measures, AUROC and their standard errors are shown. Predictors designed specifically for nucleotide variants are indicated with a '(•)'. Statistical significance relative to VARITY_ER was assessed using a one-sided Z test applied to 2,000 bootstrapped test sets (P-values are shown in brackets, with '*' indicating where P < 0.05). Other test statistics such as 95% confidence interval and effect size can be found in Table S6.

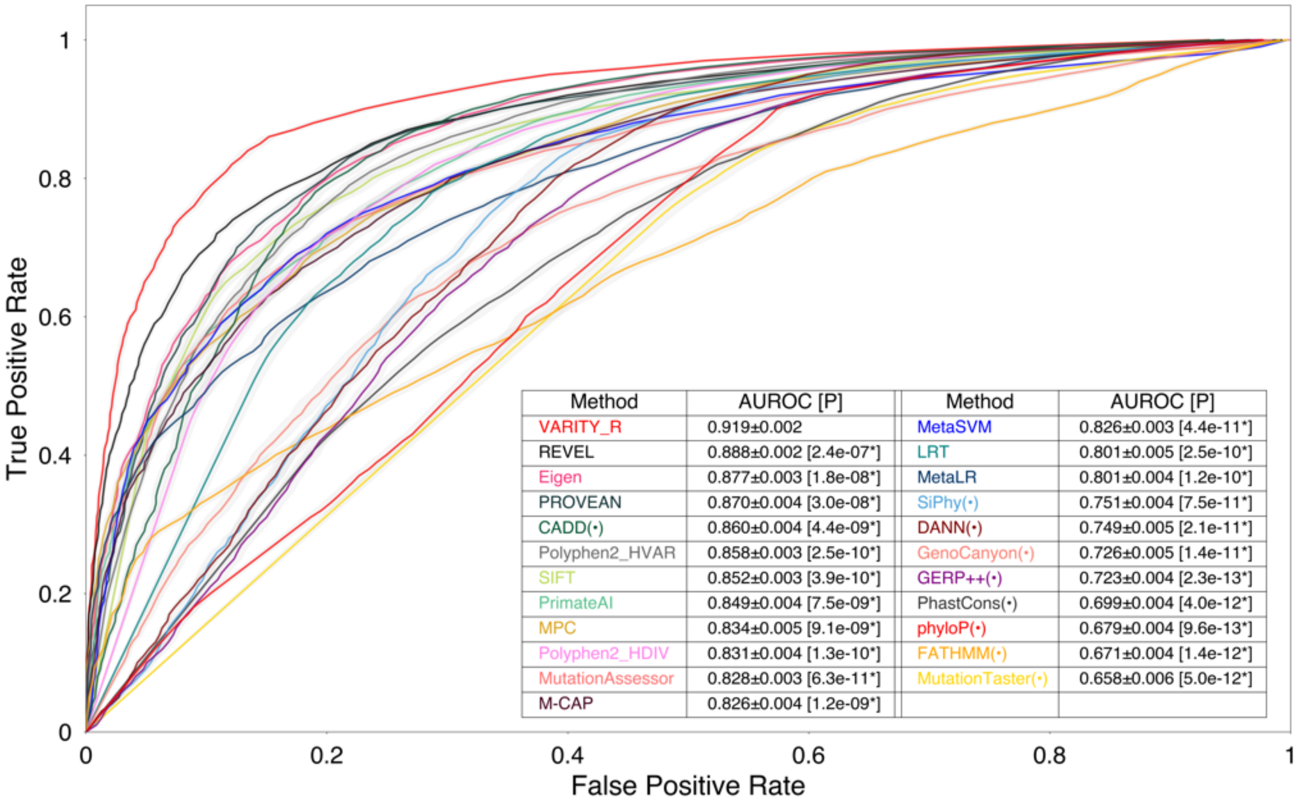

Figure S13: Comparing ROC performance of VARITY_R with other predictors for a high-quality 'core' variant set (MAF < 0.5%). We compare ROC performance for VARITY_R (using nested cross-validation) with other 23 variant pathogenicity predictors. For compactness, a predictor with AUROC smaller than 0.6 (see Table S11) is not shown. Predictors designed specifically for nucleotide variants indicated with a '(•)'. The test set was 9,719 variants (5,912 positive and 3,807 negative examples) from the core training set, after removing variants annotated by HGMD[6] and retaining only variants that had been scored by all methods. At any given false positive rate, true positive rate is averaged over all 10 outer-loop folds and the standard error is indicated by the surrounding grey region. As overall performance measure, AUROC and their standard errors are shown. Statistical significance of performance relative to VARITY_R used a one-sided paired t-test with 9 degrees of freedom (P-values are in brackets, with '*' indicating P < 0.05). Other test statistics such as 95% confidence interval and effect size are in Table S11.

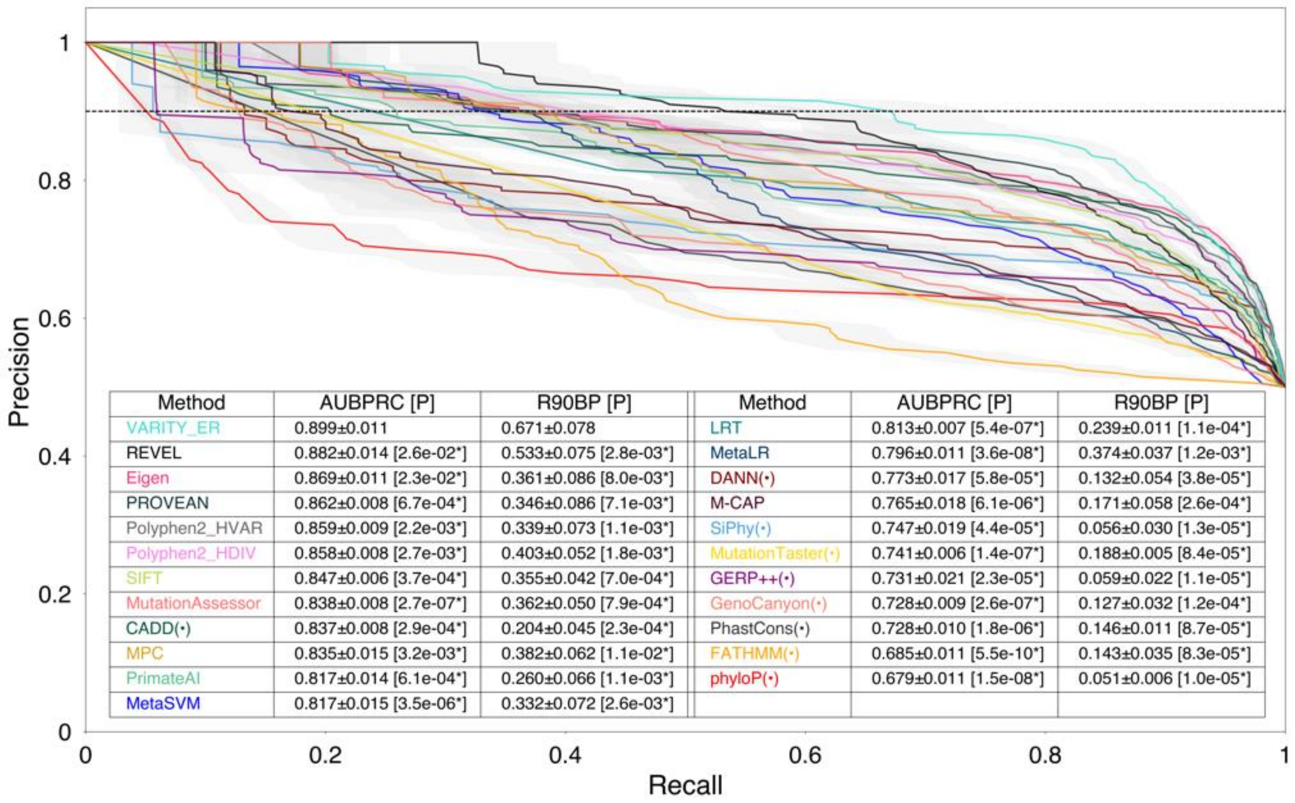

Method	AUBPRC [P]	R90BP [P]	Method	AUBPRC [P]	R90BP [P]
VARITY_ER	0.899±0.011	0.671±0.078	LRT	0.813±0.007 [5.4e-07*]	0.239±0.011 [1.1e-04*]
REVEL	0.882±0.014 [2.6e-02*]	0.533±0.075 [2.8e-03*]	MetaLR	0.796±0.011 [3.6e-08*]	0.374±0.037 [1.2e-03*]
Eigen	0.869±0.011 [2.3e-02*]	0.361±0.086 [8.0e-03*]	DANN(•)	0.773±0.017 [5.8e-05*]	0.132±0.054 [3.8e-05*]
PROVEAN	0.862±0.008 [6.7e-04*]	0.346±0.086 [7.1e-03*]	M-CAP	0.765±0.018 [6.1e-06*]	0.171±0.058 [2.6e-04*]
Polyphen2_HVAR	0.859±0.009 [2.2e-03*]	0.339±0.073 [1.1e-03*]	SiPhy(•)	0.747±0.019 [4.4e-05*]	0.056±0.030 [1.3e-05*]
Polyphen2_HDIV	0.858±0.008 [2.7e-03*]	0.403±0.052 [1.8e-03*]	MutationTaster(•)	0.741±0.006 [1.4e-07*]	0.188±0.005 [8.4e-05*]
SIFT	0.847±0.006 [3.7e-04*]	0.355±0.042 [7.0e-04*]	GERP++(•)	0.731±0.021 [2.3e-05*]	0.059±0.022 [1.1e-05*]
MutationAssessor	0.838±0.008 [2.7e-07*]	0.362±0.050 [7.9e-04*]	GenoCanyon(•)	0.728±0.009 [2.6e-07*]	0.127±0.032 [1.2e-04*]
CADD(•)	0.837±0.008 [2.9e-04*]	0.204±0.045 [2.3e-04*]	PhastCons(•)	0.728±0.010 [1.8e-06*]	0.146±0.011 [8.7e-05*]
MPC	0.835±0.015 [3.2e-03*]	0.382±0.062 [1.1e-02*]	FATHMM(•)	0.685±0.011 [5.5e-10*]	0.143±0.035 [8.3e-05*]
PrimateAI	0.817±0.014 [6.1e-04*]	0.260±0.066 [1.1e-03*]	phyloP(•)	0.679±0.011 [1.5e-08*]	0.051±0.006 [1.0e-05*]
MetaSVM	0.817±0.015 [3.5e-06*]	0.332±0.072 [2.6e-03*]			

Figure S14: Comparing balanced precision recall performance of VARITY_ER with other predictors in predicting a high-quality 'core' variant set (MAF < 10^{-6}). We compare balanced precision recall performance for VARITY_ER (using nested cross-validation) with other 23 variant pathogenicity predictors. For compactness, one predictor with AUBPRC < 0.6 is not shown (see Table S13). Predictors designed specifically for nucleotide variants indicated with a '(•)'. The test set was 5,160 variants (4,675 positive and 485 negative examples) from the core training set, after removing variants annotated by HGMD[6] and retaining only variants that had been scored by all methods. Recall was averaged over all 10 outer-loop folds and the standard error is indicated by the surrounding grey region. As overall performance measures, AUBPRC and R90BP (the black dotted line) and their standard errors are shown. Statistical significance of performance relative to VARITY_ER used a one-sided paired t-test with 9 degrees of freedom (P-values in brackets were indicated with a '*' where P < 0.05). Other test statistics such as 95% confidence interval and effect size are in Table S13.

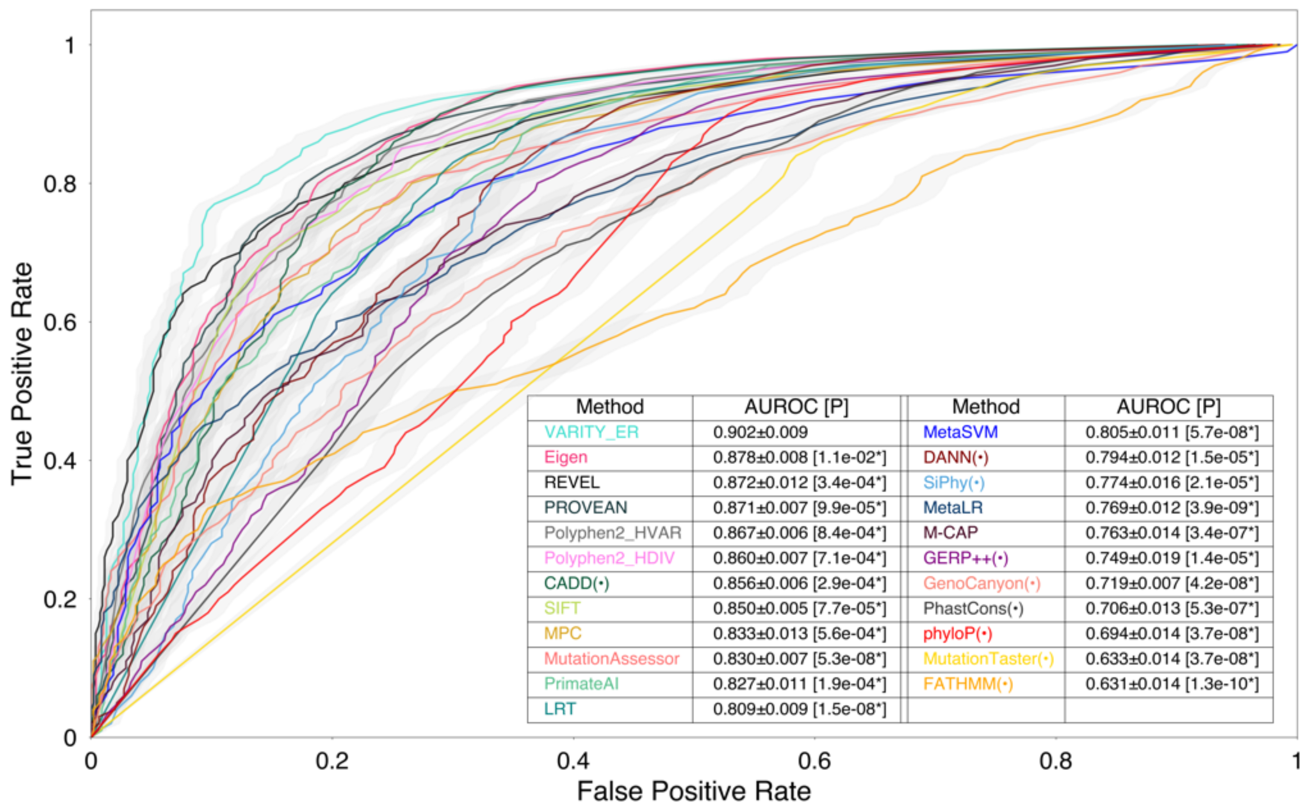

Figure S15: Comparing ROC performance of VARITY_ER with other predictors in predicting a high-quality 'core' variant set (MAF < 10^{-6}). We compare ROC performance for VARITY_ER (using nested cross-validation) with other 23 variant pathogenicity predictors. For compactness, one predictor with AUROC < 0.6 is not shown (see Table S14). Predictors designed specifically for nucleotide variants indicated with a '(•)'. The test set was 5,160 variants (4,675 positive and 485 negative examples from the core training set, after removing variants annotated by HGMD[6] and retaining only variants that had been scored by all methods. At any given false positive rate, true positive rate is averaged over all 10 outer-loop folds and the standard error is indicated by the surrounding grey region. As overall performance measure, AUROC and their standard errors are shown. Statistical significance of performance relative to VARITY_R used a one-sided paired t-test with 9 degrees of freedom (P-values in brackets were indicated with a '*' where P < 0.05). Other test statistics such as 95% confidence interval and effect size are in Table S14.

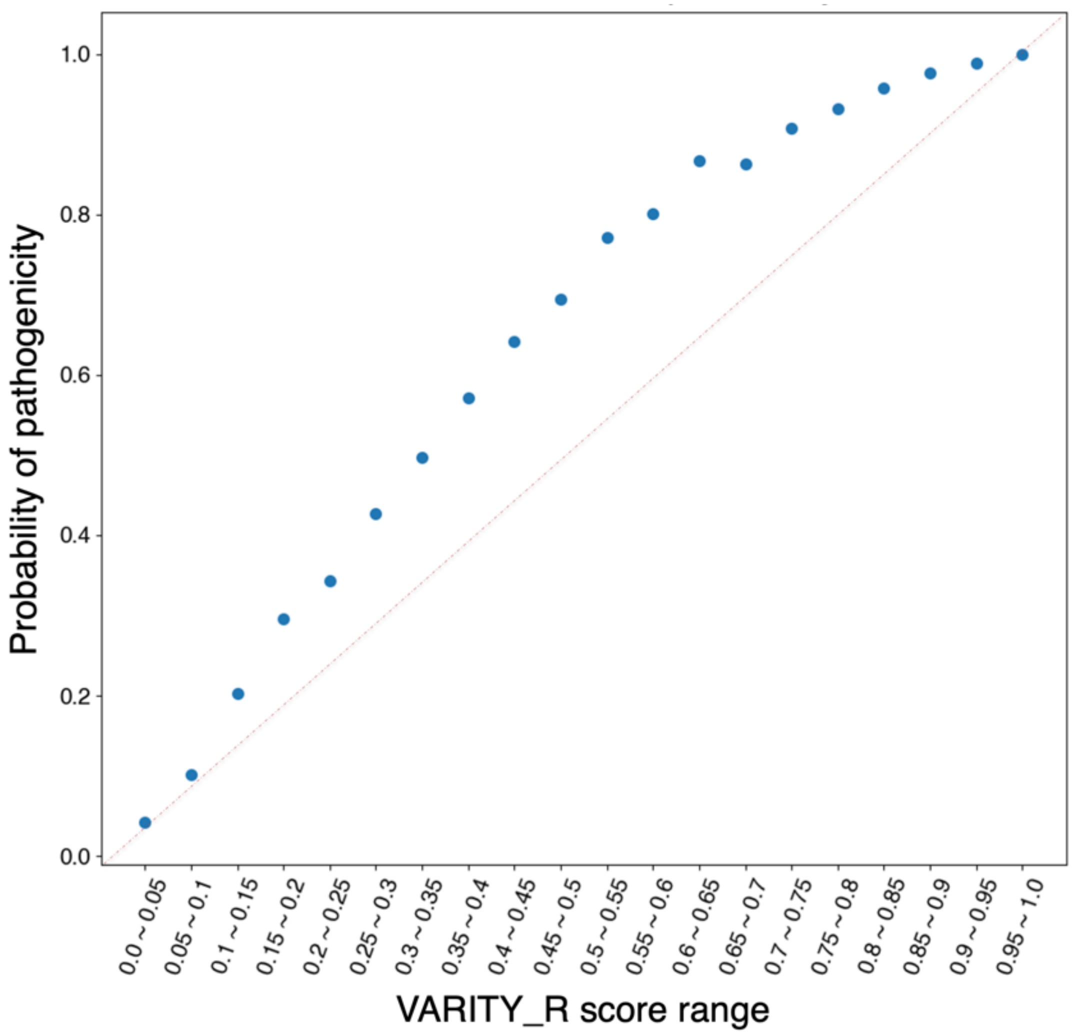

Figure S16: The relationship between VARITY_R score and probability of pathogenicity. VARITY_R scores on the 38,047 labelled core set variants (MAF < 0.5%) were used for the plot. All core set variants were assorted into 20 bins each represents a different VARITY score range (see X axis). For each bin, the probability of pathogenicity (Y axis; fraction of variants annotated as either likely pathogenic or pathogenic in ClinVar) was calculated as number of variants labelled as ‘putatively pathogenic’ divided by number of total variants in the bin.

Supplemental Tables

Table S1: Features used by VARITY models

Feature Group	Feature Names	Feature Description	Source
Conservation Scores	PROVEAN_selected_score	Provean[7] score	dbNSFP (V4.0b2)[17]
	SIFT_selected_score	SIFT[8-9] score	
	Evm_selected_score	EVMutation[10] score	
	Integrated_fitCons_score	fitCons[11] score	
	LRT_score	LRT[12] score	
	GERP++_RS	GERP++[13] score	
	phyloP30way_mammalian	phyloP[14] score	
	phastCons30way_mammalian	phastCons[15] score	
	SiPhy_29way_logOdds	SiPhy[16] score	
AA Delta properties	mw_delta	Change in molecular weight	"*Handbook of Chemistry and Physics*"[18]
	pka_delta	Change in acid dissociation constant pK_a	
	pkb_delta	Change in base dissociation constant pK_b	
	pi_delta	Change in isoelectric point pI	
	hi_delta	Change in hydropathy index	Kyte, J. & Doolittle, R. F.[19]
	pbr_delta	Change in percent buried residues	*"Proteins and proteomics: A laboratory manual"*[20]
	avbr_delta	Change in average buried residue volume	
	vadw_delta	Change in Van der Waals volume	
	asa_delta	Change in side chain accessible surface area	
	cyclic_delta	Change to/from cyclic amino acid (proline)	*"Biochemistry"*[21]
	positve_delta	Change to/from positive-charge amino acid	
	negative_delta	Change to/from negative-charge amino acid	
	charge_delta	Change to/from charged amino acid	
	Hydrophobic_delta	Change to/from hydrophobic amino acid	
	polar_delta	Change to/from polar amino acid	
	aromatic_delta	Change to/from aromatic amino acid	
	aliphatic_delta	Change to/from aliphatic amino acid	
	size_delta	Change in size	
	ionizable_delta	Change to/from ionizable amino acid	
	hbond_delta	Change to/from hydrogen-bonding amino acid	
	sulfur_delta	Change to/from sulfur-containing amino acid	
	essential_delta	Change to/from human-essential amino acid	
Secondary Structure	aa_psipred_H	Alpha Helix	PSIPRED[22]
	aa_psipred_E	Beta Sheet	
	aa_psipred_C	Coiled Coil	
Accessible Surface Area	asa_mean	Accessible Surface Area	PDBePISA[23]
Protein-protein interaction	bsa_max	Maximum solvent-accessible surface area buried by interaction partners	
	solv_ne_abs_max	Maximum solvation energy change by interaction partners	
	h_bond_max	Maximum # of hydrogen bonds with interaction partners	
	salt_bridge_max	Maximum # of salt bridges with interaction partners	
	covelent_bond_max	Maximum # of covalent bonds with interaction partners	
	disulfide_bond_max	Maximum # of disulfide bonds with interaction partners	
BLOSUM	blosum100	Blosum100	Henikoff, S. & Henikoff, J. G.[24]

In/Out Domain	in_domain	Located in vs. out of Pfam Domain	Pfam [25]

Table S2: Multiplexed Assays of Variant Effect (MAVE) variants included in training

MAVE Proteins	Uniprot ID	MIM ID	Number of Variants	Source
UBE2I	P63279	601661	2,879	Weile et al[26]
SUMO1	P63165	601912	1,779	
CALM1	P0DP23	114180	2,525	
TPK1	Q9H3S4	606370	4,124	
NCS1	P62166	603315	2,997	Sun et al., in preparation
GDI1	P31150	300104	4,365	Silverstein et al., in preparation
TECR	Q9NZ01	610057	3,756	Kishore et al., in preparation
MTHFR	P42898	607093	11,737	Weile et al[27]
CBS	P35520	613381	8,354	Sun, S. et al[28]
BRCA1	P38398	113705	1,837	Findlay, G. M. et al[29]
PTEN	P60484	601728	4,112	Matreyek, K. A. et al[30]
TPMT	P51580	187680	3,689	

Table S3: Core and add-on training sets, informative properties and hyperparameters

Resources	Training sets	# of Variants	VARITY_R	VARITY_ER	Informative Properties	Hyperparameters
ClinVAR[1]	Extremely Rare Positive (AF <10^{-6})	17,936	Core	Core	Allele frequency and ClinVAR review stars	L, k, x_0 parameters from each logistic function used for weighting are optimized for each pairing of a training set and informative property.
	Extremely Rare Negative (AF <10^{-6})	856	Core	Core		
	Moderately Rare Positive (0.5% > AF >= 10^{-6})	6,800	Core	Add-on		
	Common Positive (AF >= 0.5%)	30	Add-on			
	Moderately Rare Negative (0.5% > AF >= 10^{-6})	8,495	Core	Add-on		
	Common Negative (AF >= 0.5%)	3,864	Add-on	Add-on		
HGMD[6] (2015 version)	HGMD Positive	40,066	Add-on	Add-on	Allele frequency	
HumsaVar[2]	HumsaVar Positive	2,042	Add-on	Add-on		
	HumsaVar Common Negative (AF >= 0.5%)	1,546	Add-on	Add-on		
	HumsaVar Rare Negative (AF < 0.5%)	4,381	Add-on	Add-on		
gnomAD[3]	gnomAD Common Negative (AF >= 0.5%)	1,480	Add-on	Add-on	Allele frequency and Number of homozygotes	
	gnomAD Rare Negative (AF < 0.5%)	18,579	Add-on	Add-on		
MAVE[26-30]	MAVE Positive	20,147	Add-on	Add-on	Label confidence and Mutational accessibility	
	MAVE Negative	31,486	Add-on	Add-on		

Table S4: Algorithm level hyperparameters used in VARITY

Name	Description
n_estimators	Number of trees in the model
eta (learning rate)	Step size shrinkage used in each boosting step to prevent overfitting
gamma	Minimum loss reduction required to further partition a leaf node
max_depth	Maximum depth of a tree
min_child_weight	Minimum sum of instance weight (Hessian) needed in a child node
subsample	Subsample ratio of the training instances when constructing each tree
colsample_bytree	Subsample ratio of columns(features) when constructing each tree

Table S5: Performance on *de novo* variants from neurodevelopmental disorder studies

Test set: 215 *de novo* variants (188 positively and 27 negatively labelled examples)
Statistical test: one sided Z test based on 2,000 bootstrapped test sets

Methods	AUBPRC					R90BP				
	Value	SE	Effect Size	95% CI (one sided)	P Value (one sided)	Value	SE	Effect Size	95% CI (one sided)	P Value (one sided)
VARITY_ER	0.812	0.029				0.476	0.105			
VARITY_R	0.794	0.040	0.019	-0.005 ~ inf	1.22E-01	0.398	0.183	0.077	-0.021 ~ inf	1.90E-01
MPC[31]	0.767	0.041	0.045	-0.017 ~ inf	1.25E-01	0.366	0.165	0.109	-0.101 ~ inf	2.69E-01
MutationAssessor[32]	0.764	0.038	0.048	0.005 ~ inf	2.85E-02	0.370	0.131	0.106	-0.016 ~ inf	7.36E-02
M-CAP[33]	0.764	0.044	0.049	-0.012 ~ inf	9.85E-02	0.261	0.127	0.214	0.026 ~ inf	3.64E-02
PrimateAI[34]	0.749	0.044	0.063	0.015 ~ inf	1.81E-02	0.254	0.166	0.221	0.032 ~ inf	2.13E-02
REVEL[35]	0.747	0.040	0.065	0.010 ~ inf	2.34E-02	0.281	0.116	0.195	0.011 ~ inf	3.40E-02
Provean[7]	0.746	0.047	0.066	0.020 ~ inf	1.26E-02	0.288	0.208	0.188	-0.043 ~ inf	1.01E-01
CADD[36]	0.746	0.041	0.066	0.013 ~ inf	1.71E-02	0.289	0.138	0.187	0.016 ~ inf	4.34E-02
Polyphen2_HVAR[37-38]	0.741	0.040	0.071	0.003 ~ inf	4.52E-02	0.300	0.129	0.176	-0.112 ~ inf	1.41E-01
SIFT[8-9]	0.729	0.046	0.083	0.030 ~ inf	3.60E-03	0.157	0.138	0.319	0.033 ~ inf	6.35E-03
Eigen[39]	0.700	0.046	0.112	0.055 ~ inf	6.15E-04	0.197	0.137	0.279	0.080 ~ inf	1.03E-02
MetaLR[40]	0.699	0.040	0.113	0.043 ~ inf	3.86E-03	0.211	0.057	0.265	0.090 ~ inf	7.75E-03
MutationTaster[41]	0.697	0.039	0.116	0.068 ~ inf	7.11E-04	0.152	0.037	0.323	0.151 ~ inf	8.07E-04
Polyphen2_HDIV[37-38]	0.692	0.046	0.120	0.036 ~ inf	6.69E-03	0.145	0.094	0.331	0.054 ~ inf	9.71E-03
LRT[12]	0.685	0.038	0.127	0.072 ~ inf	2.58E-04	0.140	0.035	0.335	0.165 ~ inf	4.78E-04
MetaSVM[40]	0.680	0.043	0.132	0.063 ~ inf	8.48E-04	0.162	0.075	0.313	0.143 ~ inf	1.77E-03
FATHMM[41]	0.676	0.038	0.136	0.063 ~ inf	1.58E-03	0.202	0.056	0.273	0.085 ~ inf	8.55E-03
GenoCanyon[42]	0.647	0.034	0.165	0.093 ~ inf	3.63E-05	0.148	0.066	0.328	0.128 ~ inf	3.31E-03
GERP++[13]	0.636	0.046	0.176	0.073 ~ inf	1.18E-03	0.071	0.050	0.405	0.206 ~ inf	3.29E-04
phastCons[15]	0.626	0.052	0.187	0.082 ~ inf	1.17E-03	0.050	0.061	0.426	0.222 ~ inf	2.10E-04
SiPhy[16]	0.610	0.035	0.203	0.124 ~ inf	4.71E-06	0.044	0.060	0.432	0.228 ~ inf	2.36E-04
DANN[44]	0.596	0.031	0.217	0.136 ~ inf	2.76E-07	0.028	0.036	0.448	0.255 ~ inf	3.72E-05
phyloP[14]	0.593	0.037	0.220	0.127 ~ inf	3.97E-06	0.036	0.032	0.440	0.261 ~ inf	3.70E-05
fitCons[11]	0.590	0.041	0.222	0.135 ~ inf	1.67E-06	0.035	0.045	0.440	0.260 ~ inf	3.36E-05

Table S6: ROC performance on *de novo* variants in neurodevelopmental disorder studies

Test set: 215 *de novo* variants (188 positively and 27 negatively labelled examples)<br>Statistical test: one sided Z test based on 2,000 bootstrapped test set					
**Methods**	**AUROC**				
	**Value**	**SE**	**Effect Size**	**95% CI (one sided)**	**P Value (one sided)**
VARITY_ER	0.737	0.038			
VARITY_R	0.730	0.042	0.007	-0.014 ~ inf	2.79E-01
M-CAP[33]	0.717	0.048	0.020	-0.050 ~ inf	3.28E-01
MutationAssessor[32]	0.697	0.046	0.040	-0.020 ~ inf	1.32E-01
MPC[31]	0.694	0.046	0.043	-0.025 ~ inf	1.53E-01
PrimateAI[34]	0.685	0.048	0.052	-0.006 ~ inf	7.28E-02
CADD[36]	0.675	0.047	0.062	-0.000 ~ inf	4.59E-02
REVEL[35]	0.673	0.045	0.064	-0.001 ~ inf	4.91E-02
Polyphen2_HVAR[37-38]	0.673	0.048	0.064	-0.006 ~ inf	6.69E-02
SIFT[8-9]	0.673	0.052	0.064	0.002 ~ inf	4.15E-02
Provean[7]	0.668	0.045	0.069	0.028 ~ inf	2.87E-03
Polyphen2_HDIV[37-38]	0.644	0.053	0.093	0.011 ~ inf	3.30E-02
MetaLR[40]	0.637	0.054	0.100	0.003 ~ inf	4.21E-02
Eigen[39]	0.629	0.050	0.108	0.047 ~ inf	2.02E-03
MetaSVM[40]	0.609	0.051	0.129	0.040 ~ inf	8.87E-03
FATHMM[41]	0.599	0.051	0.139	0.035 ~ inf	1.51E-02
GERP++[13]	0.593	0.054	0.144	0.020 ~ inf	2.32E-02
LRT[12]	0.585	0.050	0.152	0.075 ~ inf	4.12E-04
MutationTaster[41]	0.585	0.048	0.152	0.085 ~ inf	8.75E-05
phastCons[15]	0.576	0.045	0.161	0.057 ~ inf	5.19E-03
phyloP[14]	0.564	0.046	0.173	0.057 ~ inf	2.79E-03
fitCons[11]	0.557	0.042	0.181	0.085 ~ inf	6.09E-04
GenoCanyon[42]	0.555	0.039	0.182	0.103 ~ inf	5.66E-05
DANN[44]	0.553	0.040	0.184	0.085 ~ inf	2.90E-04
SiPhy[16]	0.548	0.036	0.189	0.097 ~ inf	1.35E-04

Table S7: Comparing VARITY_ER with MPC[31] and M_CAP[45] Individually on *de novo* variants in neurodevelopmental disorder studies

Statistical test: one sided Z test based on 2,000 bootstrapped test set							
**Methods**	**Number of variants**	**Metrics**	**VARITY_ER**	**Value**	**Effect Size**	**95% CI**	**P Value**
MPC[31]	323<br>[P:285, N:38]	AUBPRC	0.791±0.025	0.731±0.040	0.059	0.000 ~ inf	4.79E-02
		R90BP	0.428±0.086	0.209±0.156	0.219	-0.067 ~ inf	9.43E-02
		AUROC	0.787±0.026	0.712±0.047	0.075	0.004 ~ inf	4.30E-02
M-CAP[33]	365<br>[P:320, N:45]	AUBPRC	0.765±0.025	0.709±0.035	0.056	0.007 ~ inf	2.91E-02
		R90BP	0.366±0.085	0.167±0.052	0.198	0.069 ~ inf	1.20E-02
		AUROC	0.691±0.033	0.674±0.042	0.017	-0.036 ~ inf	3.00E-01

Table S8: Pearson correlation between predictor scores and variant effect map scores

Pearson Correlation Coefficient Performance on 6 variant effect maps<br>Statistical test: one-sided paired t-test in 6 variant effect maps											
	BRCA1	***CALM1***	***CBS***	***PTEN***	***TPK1***	***VKORC1***	**PCC Mean**	**SE**	**Effect Size**	**95% CI (one sided)**	**P Value (one sided)**
VARITY_ER_LOO	0.570	0.211	0.475	0.493	0.199	0.485	0.405	0.065			
VARITY_ER	0.583	0.149	0.481	0.496	0.206	0.485	0.400	0.072	0.005	-0.016 ~ inf	3.29E-01
VARITY_R	0.554	0.123	0.444	0.444	0.226	0.471	0.377	0.067	0.028	-0.001 ~ inf	6.67E-02
VARITY_R_LOO	0.532	0.205	0.424	0.435	0.178	0.470	0.374	0.060	0.031	0.016 ~ inf	7.25E-03
REVEL[35]	0.595	0.134	0.411	0.417	0.188	0.450	0.366	0.071	0.040	0.009 ~ inf	3.14E-02
Eigen[39]	0.438	0.212	0.344	0.337	0.189	0.395	0.319	0.040	0.086	0.036 ~ inf	1.29E-02
Provean[7]	0.376	0.077	0.403	0.430	0.184	0.397	0.311	0.059	0.094	0.048 ~ inf	6.94E-03
MPC[31]	0.294	0.222	0.455	0.284	0.165	0.418	0.306	0.046	0.099	0.012 ~ inf	4.54E-02
CADD[36]	0.384	0.189	0.338	0.325	0.166	0.422	0.304	0.042	0.101	0.048 ~ inf	8.89E-03
M-CAP[33]	0.532	0.049	0.419	0.279	0.189	0.342	0.302	0.070	0.104	0.043 ~ inf	1.27E-02
MetaSVM[40]	0.453	0.131	0.287	0.244	0.218	0.435	0.295	0.052	0.111	0.038 ~ inf	1.89E-02
MetaLR[40]	0.436	0.111	0.250	0.219	0.221	0.425	0.277	0.052	0.128	0.047 ~ inf	1.68E-02
PrimateAI[34]	0.359	0.057	0.346	0.261	0.101	0.240	0.227	0.051	0.178	0.133 ~ inf	3.87E-04
SiPhy[16]	0.359	0.108	0.188	0.036	0.103	0.236	0.172	0.047	0.234	0.133 ~ inf	3.93E-03
GERP++[13]	0.290	0.123	0.168	0.054	0.125	0.257	0.170	0.036	0.236	0.131 ~ inf	4.42E-03
DANN[44]	0.165	0.068	0.175	0.131	0.070	0.290	0.150	0.034	0.255	0.168 ~ inf	1.51E-03
FATHMM[41]	0.358	0.030	0.157	0.003	0.114	0.188	0.142	0.052	0.264	0.159 ~ inf	2.81E-03
phyloP[14]	0.269	0.060	0.166	0.019	0.063	0.235	0.135	0.042	0.270	0.177 ~ inf	1.56E-03
LRT[12]	0.222	0.044	0.159	0.067	0.067	0.224	0.131	0.033	0.275	0.191 ~ inf	9.05E-04
GenoCanyon[42]	0.188	0.045	0.080	0.066	0.054	0.163	0.099	0.025	0.306	0.215 ~ inf	8.20E-04
phastCons[15]	0.161	0.002	0.155	0.009	0.043	0.139	0.085	0.031	0.320	0.229 ~ inf	6.81E-04
fitCons[11]	0.017	0.136	0.072	0.136	0.042	0.064	0.078	0.020	0.328	0.194 ~ inf	3.21E-03
MutationTaster[41]	0.073	0.065	0.111	0.047	0.001	0.049	0.058	0.015	0.348	0.240 ~ inf	9.82E-04

Table S9: Spearman correlation between predictor scores and variant effect map scores

Spearman Correlation Coefficient Performance on 6 variant effect maps<br>Statistical test: one-sided paired t-test in 6 variant effect maps											
	BRCA1	***CALM1***	***CBS***	***PTEN***	***TPK1***	***VKORC1***	**SRC Mean**	**SE**	**Effect Size**	**95% CI (one sided)**	**P Value (one sided)**
VARITY_R_LOO	0.470	0.220	0.461	0.425	0.184	0.480	0.373	0.055			
VARITY_R	0.483	0.132	0.474	0.430	0.223	0.481	0.370	0.063	0.003	-0.030 ~ inf	4.40E-01
VARITY_ER	0.473	0.151	0.458	0.435	0.198	0.485	0.367	0.061	0.007	-0.017 ~ inf	3.14E-01
VARITY_ER_LOO	0.460	0.145	0.453	0.437	0.190	0.485	0.362	0.062	0.012	-0.013 ~ inf	2.09E-01
REVEL[35]	0.520	0.121	0.411	0.401	0.182	0.453	0.348	0.065	0.025	-0.012 ~ inf	1.33E-01
Eigen[39]	0.452	0.202	0.365	0.338	0.195	0.421	0.329	0.044	0.045	0.013 ~ inf	2.49E-02
CADD[36]	0.364	0.190	0.351	0.315	0.172	0.445	0.306	0.043	0.067	0.032 ~ inf	8.10E-03
Provean[7]	0.323	0.083	0.389	0.402	0.173	0.450	0.303	0.059	0.070	0.025 ~ inf	1.73E-02
MPC[31]	0.266	0.195	0.435	0.309	0.173	0.418	0.299	0.045	0.074	0.018 ~ inf	2.91E-02
M-CAP[33]	0.493	0.045	0.406	0.277	0.203	0.349	0.295	0.065	0.078	0.013 ~ inf	3.90E-02
MetaSVM[40]	0.434	0.133	0.295	0.219	0.214	0.465	0.293	0.054	0.080	0.012 ~ inf	4.16E-02
MetaLR[40]	0.413	0.113	0.337	0.216	0.215	0.453	0.291	0.054	0.082	0.019 ~ inf	3.05E-02
PrimateAI[34]	0.302	0.026	0.338	0.280	0.103	0.239	0.214	0.050	0.159	0.117 ~ inf	4.76E-04
GERP++[13]	0.320	0.096	0.217	0.088	0.118	0.333	0.195	0.046	0.178	0.105 ~ inf	3.18E-03
LRT[12]	0.340	0.037	0.296	0.127	0.096	0.246	0.190	0.049	0.183	0.127 ~ inf	9.14E-04
DANN[44]	0.221	0.030	0.245	0.186	0.041	0.385	0.185	0.055	0.189	0.144 ~ inf	2.88E-04
GenoCanyon[42]	0.281	0.035	0.257	0.128	0.074	0.242	0.170	0.043	0.204	0.157 ~ inf	2.39E-04
SiPhy[16]	0.329	0.086	0.154	0.023	0.098	0.287	0.163	0.049	0.211	0.120 ~ inf	3.89E-03
FATHMM[41]	0.234	0.017	0.162	0.022	0.065	0.385	0.147	0.059	0.226	0.140 ~ inf	2.37E-03
phyloP[14]	0.243	0.041	0.195	0.049	0.039	0.281	0.141	0.045	0.232	0.171 ~ inf	4.67E-04
fitCons[11]	0.032	0.094	0.038	0.264	0.083	0.105	0.103	0.034	0.270	0.152 ~ inf	4.22E-03
phastCons[15]	0.182	0.036	0.147	0.058	0.014	0.143	0.097	0.028	0.277	0.216 ~ inf	2.05E-04
MutationTaster[41]	0.004	0.051	0.156	0.204	0.029	0.034	0.079	0.033	0.294	0.191 ~ inf	1.64E-03

Table S10: VARITY_R Performance (AUBPRC) comparison with 23 other predictors

Performance on core set (ClinVAR rare variants) using 10-fold nested cross validation based on 9,719 variants (5,912 positive and 3,807 negative examples; variants labelled by HGMD[6] were removed)
Statistical test: one-sided paired t-test on 10 outer-loop test sets relative to VARITY_R (9 degrees of freedom)

Methods	AUBPRC					R90BP				
	Value	SE	Effect Size	95% CI (one sided)	P Value (one sided)	Value	SE	Effect Size	95% CI (one sided)	P Value (one sided)
VARITY_R	0.920	0.003				0.752	0.012			
REVEL[35]	0.893	0.002	0.027	0.022 ~ inf	3.44E-06	0.623	0.012	0.129	0.103 ~ inf	5.64E-06
Eigen[39]	0.871	0.003	0.049	0.044 ~ inf	2.23E-08	0.494	0.024	0.258	0.218 ~ inf	7.14E-07
Provean[7]	0.862	0.005	0.058	0.050 ~ inf	1.06E-07	0.442	0.050	0.310	0.234 ~ inf	2.91E-05
SIFT[8-9]	0.853	0.002	0.067	0.062 ~ inf	2.64E-09	0.411	0.016	0.341	0.300 ~ inf	6.60E-08
PrimateAI[34]	0.846	0.005	0.074	0.067 ~ inf	7.41E-09	0.419	0.031	0.333	0.285 ~ inf	3.38E-07
Polyphen2_HVAR[3738]	0.844	0.004	0.076	0.072 ~ inf	7.64E-11	0.291	0.038	0.461	0.405 ~ inf	8.14E-08
MPC[31]	0.844	0.005	0.077	0.068 ~ inf	4.17E-08	0.429	0.021	0.323	0.282 ~ inf	1.13E-07
MutationAssessor[32]	0.838	0.003	0.082	0.076 ~ inf	3.57E-10	0.411	0.021	0.341	0.293 ~ inf	3.03E-07
MetaSVM[40]	0.832	0.003	0.088	0.081 ~ inf	1.52E-09	0.442	0.013	0.310	0.283 ~ inf	4.33E-09
CADD[36]	0.830	0.006	0.090	0.081 ~ inf	6.82E-09	0.149	0.049	0.603	0.522 ~ inf	2.06E-07
M-CAP[33]	0.828	0.006	0.092	0.082 ~ inf	3.06E-08	0.354	0.039	0.398	0.323 ~ inf	3.37E-06
Polyphen2_HDIV[3738]	0.827	0.004	0.093	0.087 ~ inf	7.05E-10	0.292	0.016	0.460	0.430 ~ inf	4.24E-10
MetaLR[40]	0.816	0.004	0.104	0.097 ~ inf	1.25E-09	0.384	0.015	0.368	0.337 ~ inf	3.39E-09
LRT[12]	0.807	0.005	0.113	0.106 ~ inf	1.76E-10	0.260	0.012	0.492	0.471 ~ inf	8.10E-12
MutationTaster[41]	0.747	0.003	0.173	0.166 ~ inf	7.56E-12	0.194	0.004	0.558	0.538 ~ inf	1.14E-12
phastCons[15]	0.714	0.003	0.207	0.200 ~ inf	6.12E-13	0.131	0.003	0.620	0.600 ~ inf	8.34E-13
GenoCanyon[42]	0.710	0.005	0.211	0.201 ~ inf	1.01E-11	0.044	0.004	0.708	0.685 ~ inf	5.56E-13
FATHMM[41]	0.702	0.004	0.218	0.209 ~ inf	7.09E-12	0.158	0.025	0.594	0.548 ~ inf	1.86E-09
SiPhy[16]	0.699	0.006	0.221	0.209 ~ inf	5.01E-11	0.009	0.004	0.743	0.722 ~ inf	2.26E-13
DANN[44]	0.695	0.005	0.225	0.216 ~ inf	6.67E-12	0.013	0.005	0.739	0.716 ~ inf	5.39E-13
GERP++[13]	0.675	0.004	0.245	0.239 ~ inf	4.69E-14	0.009	0.006	0.743	0.725 ~ inf	4.69E-14
phyloP[14]	0.650	0.004	0.270	0.263 ~ inf	1.35E-13	0.039	0.003	0.713	0.694 ~ inf	1.25E-13
fitCons[11]	0.532	0.004	0.388	0.378 ~ inf	9.66E-14	0.001	0.000	0.751	0.730 ~ inf	1.73E-13

Table S11: VARITY_R Performance (AUROC) comparison with 23 other predictors

Performance on core set (ClinVAR rare variants) using 10-fold nested cross validation based on 9,719 variants (5,912 positive 3,807 negative examples, variants labelled only by HGMD[6] were removed)
Statistical test: one-sided paired t-test on 10 outer-loop test sets relative to VARITY_R (9 degrees of freedom)

Methods	AUROC				
	Value	SE	Effect Size	95% CI (one sided)	P Value (one sided)
VARITY_R	0.919	0.002			
REVEL[35]	0.888	0.002	0.032	0.027 ~ inf	2.41E-07
Eigen[39]	0.877	0.003	0.042	0.038 ~ inf	1.76E-08
Provean[7]	0.87	0.004	0.049	0.044 ~ inf	3.02E-08
CADD[36]	0.86	0.004	0.06	0.054 ~ inf	4.42E-09
Polyphen2_HVAR[37-38]	0.858	0.003	0.062	0.058 ~ inf	2.49E-10
SIFT[8-9]	0.852	0.003	0.068	0.063 ~ inf	3.93E-10
PrimateAI[34]	0.849	0.004	0.071	0.064 ~ inf	7.45E-09
MPC[31]	0.834	0.005	0.085	0.077 ~ inf	9.06E-09
Polyphen2_HDIV[37-38]	0.831	0.004	0.089	0.084 ~ inf	1.31E-10
MutationAssessor[32]	0.828	0.003	0.092	0.087 ~ inf	6.28E-11
MetaSVM[40]	0.826	0.003	0.094	0.089 ~ inf	4.40E-11
M-CAP[33]	0.826	0.004	0.093	0.086 ~ inf	1.20E-09
MetaLR[40]	0.801	0.004	0.119	0.112 ~ inf	1.17E-10
LRT[12]	0.801	0.005	0.118	0.111 ~ inf	2.54E-10
SiPhy[16]	0.751	0.004	0.168	0.159 ~ inf	7.51E-11
DANN[44]	0.749	0.005	0.171	0.162 ~ inf	2.15E-11
GenoCanyon[42]	0.726	0.005	0.194	0.185 ~ inf	1.37E-11
GERP++[13]	0.723	0.004	0.197	0.191 ~ inf	2.27E-13
phastCons[15]	0.699	0.004	0.221	0.212 ~ inf	4.03E-12
phyloP[14]	0.679	0.004	0.24	0.232 ~ inf	9.63E-13
FATHMM[41]	0.671	0.004	0.248	0.239 ~ inf	1.40E-12
MutationTaster[41]	0.658	0.006	0.262	0.251 ~ inf	4.98E-12
fitCons[11]	0.531	0.006	0.389	0.377 ~ inf	3.10E-13

Table S12: Comparing VARITY_R performance with EVMutation[10] and DeepSequence[45]

Methods	Number of variants	Metrics	VARITY_R	Value	Effect Size	95% CI	P Value
EVMutation[10]	6,121 [P:4,517, N:1,604]	AUBPRC	0.920±0.005	0.859±0.009	0.060	0.050 ~ inf	1.46E-06
		R90BP	0.748±0.021	0.415±0.065	0.333	0.247 ~ inf	4.42E-05
		AUROC	0.921±0.004	0.864±0.007	0.057	0.051 ~ inf	5.89E-08
DeepSeqeuence[45]	200 [P:153, N:47]	AUBPRC	0.996±0.002	0.917±0.019	0.078	0.046 ~ inf	1.09E-03
		R90BP	0.982±0.009	0.696±0.088	0.286	0.144 ~ inf	3.26E-03
		AUROC	0.994±0.003	0.877±0.031	0.117	0.064 ~ inf	2.05E-03

Table S13: VARITY_ER Performance comparison (AUBRPC) with 23 other predictors

Performance on core set (ClinVAR extremely rare variants) using 10-fold nested cross validation based on 5,160 variants (4,675 positive and 485 negative examples, variants labelled only by HGMD[6] were removed)
Statistical test: one-sided paired t-test on 10 outer-loop test sets relative to VARITY_R (9 degrees of freedom)

Methods	AUBPRC					R90BP				
	Value	SE	Effect Size	95% CI (one sided)	P Value (one sided)	Value	SE	Effect Size	95% CI (one sided)	P Value (one sided)
VARITY_ER	0.899	0.011				0.671	0.078			
REVEL[35]	0.882	0.014	0.017	0.004 ~ inf	2.55E-02	0.533	0.075	0.138	0.071 ~ inf	2.82E-03
Eigen[39]	0.869	0.011	0.030	0.007 ~ inf	2.30E-02	0.361	0.086	0.310	0.128 ~ inf	7.99E-03
Provean[7]	0.862	0.008	0.037	0.023 ~ inf	6.72E-04	0.346	0.086	0.325	0.139 ~ inf	7.10E-03
Polyphen2_HVAR[37-38]	0.859	0.009	0.040	0.021 ~ inf	2.24E-03	0.339	0.073	0.332	0.196 ~ inf	1.08E-03
Polyphen2_HDIV[37-38]	0.858	0.008	0.041	0.021 ~ inf	2.70E-03	0.403	0.052	0.268	0.149 ~ inf	1.78E-03
SIFT[8-9]	0.847	0.006	0.052	0.034 ~ inf	3.73E-04	0.355	0.042	0.316	0.195 ~ inf	7.05E-04
MutationAssessor[32]	0.838	0.008	0.061	0.052 ~ inf	2.69E-07	0.362	0.050	0.309	0.189 ~ inf	7.86E-04
CADD[36]	0.837	0.008	0.062	0.041 ~ inf	2.87E-04	0.204	0.045	0.467	0.315 ~ inf	2.30E-04
MPC[31]	0.835	0.015	0.064	0.032 ~ inf	3.23E-03	0.382	0.062	0.289	0.108 ~ inf	1.08E-02
PrimateAI[34]	0.817	0.014	0.082	0.051 ~ inf	6.14E-04	0.260	0.066	0.411	0.242 ~ inf	1.12E-03
MetaSVM[40]	0.817	0.015	0.082	0.067 ~ inf	3.53E-06	0.332	0.072	0.339	0.178 ~ inf	2.61E-03
LRT[12]	0.813	0.007	0.086	0.073 ~ inf	5.39E-07	0.239	0.011	0.432	0.305 ~ inf	1.09E-04
MetaLR[40]	0.796	0.011	0.103	0.092 ~ inf	3.62E-08	0.374	0.037	0.297	0.173 ~ inf	1.23E-03
DANN[44]	0.773	0.017	0.126	0.092 ~ inf	5.81E-05	0.132	0.054	0.539	0.402 ~ inf	3.78E-05
M-CAP[33]	0.765	0.018	0.134	0.107 ~ inf	6.10E-06	0.171	0.058	0.500	0.335 ~ inf	2.62E-04
SiPhy[16]	0.747	0.019	0.152	0.113 ~ inf	4.36E-05	0.056	0.030	0.615	0.479 ~ inf	1.26E-05
MutationTaster[41]	0.741	0.006	0.158	0.137 ~ inf	1.40E-07	0.188	0.005	0.483	0.347 ~ inf	8.42E-05
GERP++[13]	0.731	0.021	0.168	0.128 ~ inf	2.34E-05	0.059	0.022	0.612	0.478 ~ inf	1.13E-05
GenoCanyon[42]	0.728	0.009	0.170	0.147 ~ inf	2.59E-07	0.127	0.032	0.544	0.383 ~ inf	1.19E-04
phastCons[15]	0.728	0.010	0.171	0.142 ~ inf	1.75E-06	0.146	0.011	0.525	0.376 ~ inf	8.68E-05
FATHMM[41]	0.685	0.011	0.214	0.199 ~ inf	5.54E-10	0.143	0.035	0.528	0.379 ~ inf	8.29E-05
phyloP[14]	0.679	0.011	0.220	0.198 ~ inf	1.48E-08	0.051	0.006	0.620	0.487 ~ inf	9.96E-06
fitCons[11]	0.563	0.007	0.336	0.317 ~ inf	1.10E-10	0.027	0.012	0.644	0.511 ~ inf	7.42E-06

Table S14: VARITY_ER Performance (AUROC) comparison with 23 other predictors

Performance on core set (ClinVAR extremely rare variants) using 10-fold nested cross validation
based on 5,160 variants (4,675 positive and 485 negative examples, variants labelled only by HGMD[6] were removed)
Statistical test: one-sided paired t-test on 10 outer-loop test sets relative to VARITY_R (9 degrees of freedom)

Methods	AUROC				
	Value	SE	Effect Size	95% CI (one sided)	P Value (one sided)
VARITY_ER	0.902	0.009			
Eigen[39]	0.878	0.008	0.024	0.009 ~ inf	1.10E-02
REVEL[35]	0.872	0.012	0.030	0.020 ~ inf	3.41E-04
Provean[7]	0.871	0.007	0.031	0.022 ~ inf	9.89E-05
Polyphen2_HVAR[37-38]	0.867	0.006	0.035	0.021 ~ inf	8.43E-04
Polyphen2_HDIV[3738]	0.860	0.007	0.042	0.026 ~ inf	7.12E-04
CADD[36]	0.856	0.006	0.047	0.031 ~ inf	2.91E-04
SIFT[8-9]	0.850	0.005	0.052	0.038 ~ inf	7.71E-05
MPC[31]	0.833	0.013	0.069	0.043 ~ inf	5.64E-04
MutationAssessor[32]	0.830	0.007	0.072	0.064 ~ inf	5.27E-08
PrimateAI[34]	0.827	0.011	0.075	0.051 ~ inf	1.94E-04
LRT[12]	0.809	0.009	0.093	0.084 ~ inf	1.52E-08
MetaSVM[40]	0.805	0.011	0.097	0.086 ~ inf	5.72E-08
DANN[44]	0.794	0.012	0.108	0.083 ~ inf	1.51E-05
SiPhy[16]	0.774	0.016	0.128	0.098 ~ inf	2.08E-05
MetaLR[40]	0.769	0.012	0.134	0.122 ~ inf	3.88E-09
M-CAP[33]	0.763	0.014	0.139	0.119 ~ inf	3.44E-07
GERP++[13]	0.749	0.019	0.153	0.119 ~ inf	1.39E-05
GenoCanyon[42]	0.719	0.007	0.183	0.162 ~ inf	4.19E-08
phastCons[15]	0.706	0.013	0.196	0.166 ~ inf	5.32E-07
phyloP[14]	0.694	0.014	0.208	0.185 ~ inf	3.71E-08
MutationTaster[41]	0.633	0.014	0.270	0.240 ~ inf	3.66E-08
FATHMM[41]	0.631	0.014	0.271	0.256 ~ inf	1.33E-10
fitCons[11]	0.538	0.007	0.364	0.342 ~ inf	1.40E-10

Table S15: Comparing VARITY_ER Performance with EVMutation[10]

	Size	Metrics	VARITY_R	Value	Effect Size	95% CI	P Value
EVMutation[10]	3,993 [P:3,794, N:199]	AUBPRC	0.886±0.017	0.866±0.013	0.020	0.001 ~ inf	5.37E-02
		R90BP	0.449±0.106	0.409±0.072	0.040	-0.117 ~ inf	3.35E-01
		AUROC	0.886±0.016	0.858±0.012	0.027	0.011 ~ inf	7.61E-03

References

1. Landrum, M.J., Lee, J.M., Benson, M., Brown, G.R., Chao, C., Chitipiralla, S., Gu, B., Hart, J., Hoffman, D., Jang, W., et al. (2018). ClinVar: Improving access to variant interpretations and supporting evidence. Nucleic Acids Res. 46, D1062–D1067.
2. The UniProt Consortium (2017). UniProt: the universal protein knowledgebase. Nucleic Acids Res. 45, D158.
3. Karczewski, K.J., Francioli, L.C., Tiao, G., Cummings, B.B., Alföldi, J., Wang, Q., Collins, R.L., Laricchia, K.M., Ganna, A., Birnbaum, D.P., et al. (2020). The mutational constraint spectrum quantified from variation in 141,456 humans. Nature 581, 434–443.
4. Bergstra, J., Bardenet, R., Bengio, Y., and Kégl, B. (2011). Algorithms for Hyper-Parameter Optimization. Adv. Neural Inf. Process. Syst. 24.
5. Bergstra, J., Komer, B., Eliasmith, C., Yamins, D., and Cox, D.D. (2015). Hyperopt: A Python library for model selection and hyperparameter optimization. Comput. Sci. Discov. 8.
6. Stenson, P.D., Mort, M., Ball, E. V., Evans, K., Hayden, M., Heywood, S., Hussain, M., Phillips, A.D., and Cooper, D.N. (2017). The Human Gene Mutation Database: towards a comprehensive repository of inherited mutation data for medical research, genetic diagnosis and next-generation sequencing studies. Hum. Genet. 136, 665–677.
7. Choi, Y., Sims, G.E., Murphy, S., Miller, J.R., and Chan, A.P. (2012). Predicting the Functional Effect of Amino Acid Substitutions and Indels. PLoS One 7.
8. Kumar, P., Henikoff, S. & Ng, P. C. Predicting the effects of coding non-synonymous variants on protein function using the SIFT algorithm. *Nat. Protoc.* **4**, 1073–1082 (2009).
9. Vaser, R., Adusumalli, S., Leng, S.N., Sikic, M., and Ng, P.C. (2016). SIFT missense predictions for genomes. Nat. Protoc. 11, 1–9.
10. Hopf, T.A., Ingraham, J.B., Poelwijk, F.J., Schärfe, C.P.I., Springer, M., Sander, C., and Marks, D.S. (2017). Mutation effects predicted from sequence co-variation. Nat. Biotechnol. 35, 128–135.
11. Gulko, B., Hubisz, M.J., Gronau, I., and Siepel, A. (2015). A method for calculating probabilities of fitness consequences for point mutations across the human genome. Nat. Genet. 47, 276–283.

12. Chun, S., and Fay, J.C. (2009). Identification of deleterious mutations within three human genomes. Genome Res. 19, 1553–1561.

13. Davydov, E. V., Goode, D.L., Sirota, M., Cooper, G.M., Sidow, A., and Batzoglou, S. (2010). Identifying a high fraction of the human genome to be under selective constraint using GERP++. PLoS Comput. Biol. 6,.

14. Pollard, K.S., Hubisz, M.J., Rosenbloom, K.R., and Siepel, A. (2010). Detection of nonneutral substitution rates on mammalian phylogenies. Genome Res. 20, 110–121.

15. Siepel, A., Bejerano, G., Pedersen, J.S., Hinrichs, A.S., Hou, M., Rosenbloom, K., Clawson, H., Spieth, J., Hillier, L.D.W., Richards, S., et al. (2005). Evolutionarily conserved elements in vertebrate, insect, worm, and yeast genomes. Genome Res. 15, 1034–1050.

16. Garber, M., Guttman, M., Clamp, M., Zody, M.C., Friedman, N., and Xie, X. (2009). Identifying novel constrained elements by exploiting biased substitution patterns. Bioinformatics 25, 54–62.

17. Liu, X., Wu, C., Li, C., and Boerwinkle, E. (2016). dbNSFP v3.0: A One-Stop Database of Functional Predictions and Annotations for Human Nonsynonymous and Splice-Site SNVs. Hum. Mutat. 37, 235–241.

18. D.R. Lide. (2006) CRC Handbook of Chemistry and Physics, 86th Edition (Boca Raton, FL: CRC Press)

19. J, K., and RF, D. (1982) A simple method for displaying the hydropathic character of a protein. J. Mol. Biol. 157, 105–132.

20. Simpson, R. J. (2003) Proteins and proteomics: A laboratory manual. (Cold Spring Harbor, NY: Cold Spring Harbor Laboratory Press)

21. Garrett, R. H., & Grishm, C. M. (2010) Biochemistry. (Belmont, CA: Brooks/Cole, Cengage Learning)

22. Buchan, D.W.A., and Jones, D.T. (2019). The PSIPRED Protein Analysis Workbench: 20 years on. Nucleic Acids Res. 47, W402–W407.

23. Krissinel, E., and Henrick, K. (2007). Inference of Macromolecular Assemblies from Crystalline State. J. Mol. Biol. 372, 774–797.

24. Henikoff, S., and Henikoff, J.G. (1992). Amino acid substitution matrices from protein blocks. Proc. Natl. Acad. Sci. U. S. A. 89, 10915–10919.

25. El-Gebali, S., Mistry, J., Bateman, A., Eddy, S.R., Luciani, A., Potter, S.C., Qureshi, M., Richardson, L.J., Salazar, G.A., Smart, A., et al. (2019). The Pfam protein families database in 2019. Nucleic Acids Res. 47, D427–D432.

26. Weile, J., Sun, S., Cote, A.G., Knapp, J., Verby, M., Mellor, J.C., Wu, Y., Pons, C., Wong, C., Lieshout, N., et al. (2017). A framework for exhaustively mapping functional missense variants. Mol. Syst. Biol. 13, 957.

27. Weile, J., Kishore, N., Sun, S., Maaieh, R., Verby, M., Li, R., Fotiadou, I., Kitaygorodsky, J., Wu, Y., Holenstein, A., et al. (2021). Shifting landscapes of human MTHFR missense-variant effects. Am. J. Hum. Genet. 108, 1283–1300.

28. Sun, S., Weile, J., Verby, M., Wu, Y., Wang, Y., Cote, A.G., Fotiadou, I., Kitaygorodsky, J., Vidal, M., Rine, J., et al. (2020). A proactive genotype-to-patient-phenotype map for cystathionine beta-synthase. Genome Med. 12, 1–18.

29. Findlay, G.M., Daza, R.M., Martin, B., Zhang, M.D., Leith, A.P., Gasperini, M., Janizek, J.D., Huang, X., Starita, L.M., and Shendure, J. (2018). Accurate classification of BRCA1 variants with saturation genome editing. Nature 562, 217–222.

30. Matreyek, K.A., Starita, L.M., Stephany, J.J., Martin, B., Chiasson, M.A., Gray, V.E., Kircher, M., Khechaduri, A., Dines, J.N., Hause, R.J., et al. (2018). Multiplex assessment of protein variant abundance by massively parallel sequencing. Nat. Genet. 50, 874–882.

31. Samocha, K., Kosmicki, J., Karczewski, K., O'Donnell-Luria, A., Pierce-Hoffman, E., MacArthur, D., Neale, B., and Daly, M. (2017). Regional missense constraint improves variant deleteriousness prediction. BioRxiv 148353.

32. Choi, Y., Sims, G.E., Murphy, S., Miller, J.R., and Chan, A.P. (2012). Predicting the Functional Effect of Amino Acid Substitutions and Indels. PLoS One 7.

33. Jagadeesh, K.A., Wenger, A.M., Berger, M.J., Guturu, H., Stenson, P.D., Cooper, D.N., Bernstein, J.A., and Bejerano, G. (2016). M-CAP eliminates a majority of variants of uncertain significance in clinical exomes at high sensitivity. Nat. Genet. 48, 1581–1586.

34. Sundaram, L., Gao, H., Padigepati, S.R., McRae, J.F., Li, Y., Kosmicki, J.A., Fritzilas, N., Hakenberg, J., Dutta, A., Shon, J., et al. (2018). Predicting the clinical impact of human mutation with deep neural networks. Nat. Genet. 50, 1161–1170.

35. Ioannidis, N.M., Rothstein, J.H., Pejaver, V., Middha, S., McDonnell, S.K., Baheti, S., Musolf, A., Li, Q., Holzinger, E., Karyadi, D., et al. (2016). REVEL: An Ensemble Method for Predicting the Pathogenicity of Rare Missense Variants. Am. J. Hum. Genet. 99, 877–885.

36. Kircher, M., Witten, D.M., Jain, P., O'roak, B.J., Cooper, G.M., and Shendure, J. (2014). A general framework for estimating the relative pathogenicity of human genetic variants. Nat. Genet. 46, 310–315.

37. Adzhubei, I.A., Schmidt, S., Peshkin, L., Ramensky, V.E., Gerasimova, A., Bork, P., Kondrashov, A.S., and Sunyaev, S.R. (2010). A method and server for predicting damaging missense mutations. Nat. Methods 7, 248–249.

38. Adzhubei, I., Jordan, D.M., and Sunyaev, S.R. (2013). Predicting functional effect of human missense mutations using PolyPhen-2. Curr. Protoc. Hum. Genet. Chapter 7.

39. Ionita-Laza, I., Mccallum, K., Xu, B., and Buxbaum, J.D. (2016). A spectral approach integrating functional genomic annotations for coding and noncoding variants. Nat. Genet. 48, 214–220.

40. Dong, C., Wei, P., Jian, X., Gibbs, R., Boerwinkle, E., Wang, K., and Liu, X. (2015). Comparison and integration of deleteriousness prediction methods for nonsynonymous SNVs in whole exome sequencing studies. Hum. Mol. Genet. 24, 2125–2137.

41. Schwarz, J.M., Cooper, D.N., Schuelke, M., and Seelow, D. (2014). Mutationtaster2: Mutation prediction for the deep-sequencing age. Nat. Methods 11, 361–362.

42. Shihab, H.A., Gough, J., Cooper, D.N., Day, I.N.M., and Gaunt, T.R. (2013). Predicting the functional consequences of cancer-associated amino acid substitutions. Bioinformatics 29, 1504–1510.

43. Lu, Q., Hu, Y., Sun, J., Cheng, Y., Cheung, K.H., and Zhao, H. (2015). A statistical framework to predict functional non-coding regions in the human genome through integrated analysis of annotation data. Sci. Rep. 5, 1–13.

44. Quang, D., Chen, Y., and Xie, X. (2015). DANN: A deep learning approach for annotating the pathogenicity of genetic variants. Bioinformatics 31, 761–763.

45. Riesselman, A.J., Ingraham, J.B., and Marks, D.S. (2018). Deep generative models of genetic variation capture the effects of mutations. Nat. Methods 15, 816–822.
