# Supplementary material for: Improved pathogenicity prediction for rare human missense variants
Source: Am J Hum Genet. 2021 Sep 21;108(10):1891–906. doi: 10.1016/j.ajhg.2021.08.012 (PMC8546039; doi:10.1016/j.ajhg.2021.08.012)
Supplement: Document S2. Article plus supplemental information [file mmc2.pdf]

Improved pathogenicity prediction for rare human missense variants

Yingzhou Wu,[1,2,3,4] Roujia Li,[1,2,3,4] Song Sun,[1,2,3,4] Jochen Weile,[1,2,3,4] and Frederick P. Roth[1,2,3,4,5,6,*]

The success of personalized genomic medicine depends on our ability to assess the pathogenicity of rare human variants, including the important class of missense variation. There are many challenges in training accurate computational systems, e.g., in finding the balance between quantity, quality, and bias in the variant sets used as training examples and avoiding predictive features that can accentuate the effects of bias. Here, we describe VARITY, which judiciously exploits a larger reservoir of training examples with uncertain accuracy and representativity. To limit circularity and bias, VARITY excludes features informed by variant annotation and protein identity. To provide a rationale for each prediction, we quantified the contribution of features and feature combinations to the pathogenicity inference of each variant. VARITY outperformed all previous computational methods evaluated, identifying at least 10% more pathogenic variants at thresholds achieving high (90% precision) stringency.

Introduction

Fully realizing the clinical potential of human genome sequencing will require the ability to accurately determine the pathogenicity of individual sequence variants. Although genome-wide association (GWA) studies have limited power to associate rare missense variants with disease,[1,2] rare missense changes cannot be ignored: over 99% of the set of observed missense variants are rare (with a global minor allele frequency [MAF] below 0.5%), and 90% are extremely rare (with MAF $< 10^{-6}$). Moreover, if genome sequences were known for all humans alive today, each single-nucleotide variant that is compatible with life would appear an average of ~50 times.[3] Because identifying the subset of rare missense variants that are damaging represents a major unmet challenge for personal genome interpretation, we focus here on rare and extremely rare missense changes.

It has become possible to experimentally assess the functional impact of nearly all possible missense variants for a target protein, yielding "deep mutational scans" or "variant effect maps."[3–13] However, the MaveDB[14] resource currently contains variant effect maps for fewer than 1% of the ~4,000 human disease-associated proteins, and a high-quality fully-comprehensive experimental atlas of functional missense variation could be decades away.

By contrast, computational methods to infer variant pathogenicity can already be applied at genome scale. However, performance of current methods[15–42] has not fully addressed the challenge of variant interpretation. For example, using thresholds such that 90% of pathogenicity predictions are correct, such methods identified only 10%–20% of pathogenic variants.[12] Current guidelines for clinical variant interpretation recommend that all computational methods be (at best) treated as "weak evidence."[43]

To improve computational predictors, we would like to increase the size of the training dataset while also ensuring that the quality of training data is high, i.e., the training examples are accurately labeled and representative of the variants for which pathogenicity inference is most needed (e.g., rare variants). However, there is a tension between these competing objectives. For example, although common variants can be more confidently annotated as truly benign and are therefore likely to offer higher labeling accuracy, there is evidence that common benign variants are not representative of rare benign variants.[16] However, excluding common variants would substantially reduce the number of clinically annotated benign variants available for training computational models (Figure S1). While ClinVar[44] requires clinical interpretations to be based on a rigorous and attributable process, we can be less sure of accuracy for other resources offering pathogenicity annotations. For example, HumsaVar[45] offers annotations solely from literature reports and HGMD[46] seeks to maximize recall while accepting the risk of including false positives. The variants reported in gnomAD[47] have been used as "putatively-benign" training examples by many computational methods,[16,17] despite the potential for contamination with pathogenic variants, especially in genes associated with late-onset diseases. MaveDB[14] provides experimental "variant effect maps" in which the functional impact of many variants has been measured in parallel, but the set of scored variants may not be representative of the spectrum of patient variation.

Here, we describe the VARITY approach for pathogenicity prediction, which has been specifically optimized for rare missense variation. While VARITY uses a meta-prediction

[1]The Donnelly Centre, University of Toronto, Toronto, ON M5S 3E1, Canada; [2]Department of Molecular Genetics, University of Toronto, Toronto, ON M5S 3E1, Canada; [3]Department of Computer Science, University of Toronto, Toronto, ON M5S 2E4, Canada; [4]Lunenfeld-Tanenbaum Research Institute, Sinai Health, Toronto, ON M5G 1X5, Canada; [5]Center for Cancer Systems Biology, Dana Farber Cancer Institute, Boston, MA 02215, USA; [6]Canadian Institute for Advanced Research, Toronto, ON M5G 1Z8, Canada
*Correspondence: fritz.roth@utoronto.ca
https://doi.org/10.1016/j.ajhg.2021.08.012.
© 2021 The Authors. This is an open access article under the CC BY license (http://creativecommons.org/licenses/by/4.0/).

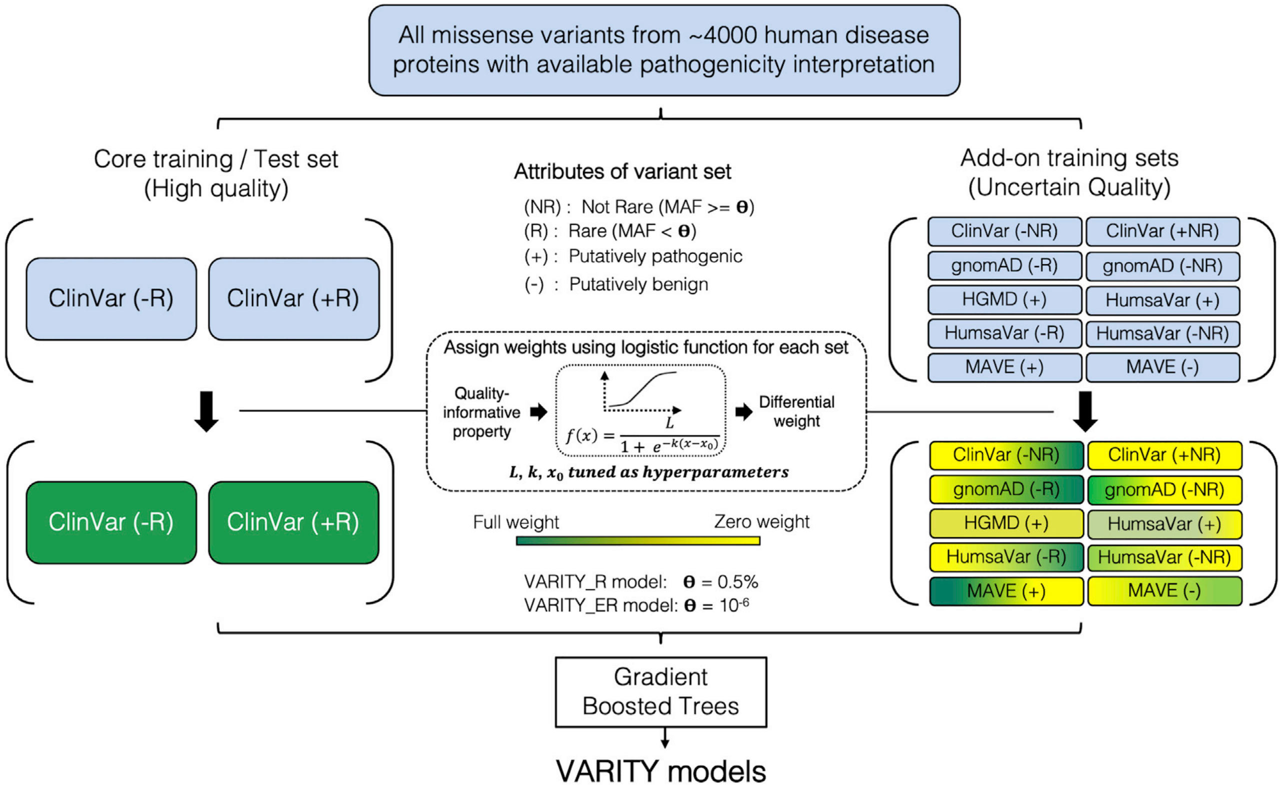

Figure 1. The VARITY framework

VARITY models were trained with an extension of the gradient boosted trees algorithm that allows different weights to be placed on different training examples. The VARITY training data consist of a high-quality core set of variants (also used as test set) and a handful of diverse add-on sets with potentially lower predictive utility. For each training set (core or add-on set), one or more quality-informative properties were each used as an input to a logistic function that calculates a weight for each variant in the set. The parameters of each logistic function were treated as hyperparameters and optimized for performance on the core set of variants via 10-fold cross-validation. We generated two VARITY models: VARITY_R, which included only rare (MAF < 0.5%) ClinVar[44] variants in its core set, and VARITY_ER, which only included extremely rare (MAF $< 10^{-6}$) ClinVar[44] variants in its core set.

strategy, it limits the circularity that can arise in such approaches by excluding any feature that was informed by variant pathogenicity annotation. We also exclude features that may serve as proxies for protein identity because these may lead to predictions that are biased by the fraction of each protein's variants in the training set that are annotated pathogenic (which may be inaccurate in future application settings). VARITY judiciously harnesses a larger set of training examples with uncertain accuracy and representativity and uses differential weighting strategies to ensure that training set expansion improves performance on a high-quality test set (Figure 1). For the task of identifying rare pathogenic variants, we find that VARITY outperforms all other computational approaches examined.

Material and methods

Assembling a resource of labeled variants and their features for human proteins

To assemble a resource of labeled variants and associated features for human proteins, we first retrieved from dbNSFP (V4.0b2)[48] all missense variants for ~18,000 human proteins that had a "reviewed" status in UniProt[45] [download date: 2019-04-08]). We next assembled variant features (summarized in Table S1) within four main categories. First, from dbNSFP[48] we collected scores for existing predictors based on only conservation information learned via unsupervised learning methods, e.g., the SIFT[25,26] method. To avoid circularity in subsequent performance testing, we excluded as features those predictors that had made direct or indirect use of annotated pathogenic and benign variants in training,[16–23] e.g., the PolyPhen2[19,20] method. Where multiple scores for a missense variant were available from a given predictor due to different coding isoforms, the score from the canonical isoform (defined in UniProt[45]) was chosen as the final score. Where multiple scores were available for different nucleotide-level changes yielding the same missense variant, the most deleterious score was chosen as the final score. Second, we defined a set of "delta values" by the difference in various quantitative physicochemical properties (e.g., molecular weight, polarity, or charge) between the reference and substituted amino acid. Third, we assembled features related to protein-protein interaction. For example, "maximum buried area"—defined by the largest solvent-accessible surface area around a given amino acid that is buried by any protein interaction partner—or the "maximum solvation energy change" —defined for a given amino acid by the greatest change in solvation energy provided by any interaction partner (both were estimated by PDBePISA[49]). Fourth, we assembled

structure-related features. These included a set of binary variables indicating secondary structure (obtained from PSIPRED[50]) and accessible surface area (estimated by PDBePISA[49]). In addition, we used substitution matrix (BLOSUM100[15]) scores and whether the amino acid position is within or outside of a Pfam[51] (download date: 2019-04-08) domain. All 43 features and their sources can be found in Table S1. The resource of features for all missense variants from ~18,000 human proteins can be downloaded via the VARITY web portal (see web resources).

For the purpose of training, we first identified a set of ~4,000 human disease-related proteins from all ~18,000 human proteins on the basis of whether any variant (not restricted to missense variants) had been annotated as "pathogenic" or "likely pathogenic" in the ClinVar database[44] or "deleterious" in the HumsaVar database[45] (download date: 2019-04-08). We further included a list of human proteins from multiplexed assays of variant effect (MAVE) studies[6–10] (see Table S2). Only variants from the set of human disease-related proteins were labeled and considered in model training. We assigned every missense variant from this set a label of either positive (putatively pathogenic), negative (putatively benign), or unknown. At this stage, our confidence in these labels might be quite low, but we will later depend on the machine-learning strategy to weight these variants. The labeling criteria depended on the source of information about the variant. For the ClinVar[44] database, regardless of the number of "review stars," variants with "pathogenic," "likely pathogenic," or "pathogenic/likely pathogenic" annotation were labeled as positive. Similarly, variants with "benign," "likely benign," or "benign/likely benign" were labeled as negative for training purposes. All other variants were labeled as unknown. For the HumsaVar[45] database, variants with the "polymorphism" annotation were labeled as negative, variants with "deleterious" annotation were labeled as positive, and all other variants were labeled as unknown. For gnomAD,[47] variants for which at least one homozygous individual had been observed were labeled as negative, and other variants were labeled as unknown. For variants from MAVE studies[6–10] (encompassing 12 proteins, see Table S2), we used functional impact scores rescaled by a previously developed pipeline,[52] such that a score of 0 indicates fitness approximating that of a nonsense variant and a score of 1 indicates fitness approximating that of a synonymous variant. Here, variants with a score above 0.5 were labeled as negative, while those below 0.5 were labeled as Positive. For the HGMD[46] database, all variants were labeled as positive. To handle the minority of cases (~10% of all variants) where a variant appears in multiple databases, we assigned a primary source for that variant by using a somewhat arbitrary ranking of the sources from which to derive the variant label: ClinVar,[44] HGMD,[46] and HumsaVar[45] pathogenicity annotations, fitness scores from variant effect maps, and presence of homozygotes in gnomAD.[47]

Core and add-on training sets

All missense variants labeled as positive or negative were considered as potential training examples. To train and test VARITY models, we assembled a core training set of variants that were of high quality (i.e., accuracy and representativity). We also used various "add-on" training sets that potentially have less predictive utility (e.g., because they are less accurately labeled or less representative of the core set), but these sets were subjected to further weighting. To obtain high-accuracy core sets, we used ClinVar[44] missense variants. To ensure high representativity, we first separated ClinVar[44] missense variants into three subsets: ClinVar_C, ClinVar_MR, and ClinVar_ER with labeled missense variants that were common (MAF $>$ 0.5%), moderately rare ($10^{-6} <$ MAF $<$ 0.5%), and extremely rare (MAF $< 10^{-6}$), respectively. Given evidence that common variants are not representative of rare variants,[16] we excluded ClinVar_C from the core training set of the VARITY model optimized for rare missense variants (VARITY_R; see Figure 1) and treated it as an add-on set. The properties of moderately rare and common variation may differ from those of extremely rare variants. Therefore, to optimize a VARITY model (VARITY_ER; see Figure 1) for extremely rare missense variants, we used ClinVar_ER as the core set and used ClinVar_C and ClinVar_MR as add-on sets.

All labeled missense variants outside of the core set were considered as add-on training examples and were separated into different add-on sets so that each set can be employed separately with distinct effects on training VARITY models. Add-on variants were first grouped into add-on training sets on the basis of source of annotation (the source where the variant label was determined) and label (positive or negative). Each negative-labeled add-on set (e.g., negative-labeled variants from gnomAD[47]) was further split on the basis of rarity (MAF cutoff = 0.5%) except the negative set from MAVE studies in which most scored variants are rare. A full list of core set and add-on sets for both VARITY_R and VARITY_ER models can be found in Table S3.

Performance measures

To evaluate performance of the VARITY models, we used the standard area under the receiver operating characteristic curve (AUROC). However, we also wished to use measures based on precision and recall, which often correspond more closely to the user's needs and intuition. Unfortunately, any measure using precision, such as the area under the precision recall curve (AUPRC), changes depending on the prior (the frequency of positively labeled examples). One strategy to compare AUPRC measures applied to different test sets with varying priors is to force each test set to be balanced, e.g., by down-sampling the most abundant label class. However, this approach destroys information by eliminating test data. Therefore, we developed two measures: area under the balanced precision recall curve (AUBPRC) and recall at 90% balanced precision (RB90P). For these measures, we simply calculate the precision for each test set and then derive the "balanced precision," i.e., the precision that would have been expected had the prior been balanced (equal to 50%):

$$balanced\ pecision = \frac{precision * (1 - prior)}{precision * (1 - prior) + (1 - precision) * prior} \quad \text{(Equation 1)}$$

Proof:

Let Y be a random variable representing the true label of an event (e.g., $Y = 1$ if a variant is pathogenic) and Y' represent the label predicted by a model (e.g., $Y' = 1$ if the model predicts a variant to be pathogenic). Precision and prior of the model can then be written as

$$precision = P(Y = 1|Y' = 1),\ prior = P(Y = 1) \quad \text{(Equation 2)}$$

The following equation holds according to Bayes Rule:

$$\frac{P(Y = 1|Y' = 1)}{P(Y = 0|Y' = 1)} = \frac{P(Y' = 1|Y = 1)}{P(Y' = 1|Y = 0)} * \frac{P(Y = 1)}{P(Y = 0)} \quad \text{(Equation 3)}$$

Using Equation 2 and Equation 3, we can rewrite as:

$$\frac{precision}{1 - precision} = L * \frac{prior}{1 - prior},\ where\ L = \frac{P(Y' = 1|Y = 1)}{P(Y' = 1|Y = 0)}$$
(Equation 4)

and then from Equation 4, we obtain the likelihood ratio

$$L = \frac{(1 - prior) * precision}{prior * (1 - precision)}$$ (Equation 5)

Because we know the likelihood ratio is not affected by the prior (ratio of conditional probability conditioned on prior), therefore we can write down the following, given a balanced prior of 0.5:

$$\frac{balanced\_precision}{1 - balanced\_precision} = L * \frac{0.5}{1 - 0.5}$$ (Equation 6)

By combining Equation 5 and Equation 6, we can solve for *balanced_precision* and obtain Equation 1.

A balanced precision versus recall curve (BPRC) can be drawn by calculation of the balanced precision value corresponding to every precision value, and AUBPRC can be calculated just as AUPRC would be. We note that there are different ways to calculate an AUPRC (or AUBPRC). Here, we calculated AUPRC (and AUBPRC) as the weighted mean of precision (or balanced precision) at different recall thresholds and used the increase in recall from the previous threshold as the weight, which is also called average precision. It can also be shown that AUBPRC can be calculated more directly from AUPRC via

$$AUBPRC = \frac{AUPRC * (1 - Prior)}{AUPRC * (1 - Prior) + (1 - AUPRC) * Prior}$$
(Equation 7)

To derive RB90P, we need to first plot the BPRC and then locate (or interpolate) the recall value at which balanced precision is 90%.

The learning algorithm and weighting of training variants

For VARITY we adopted a variant of the gradient boosted tree (GBT) machine-learning algorithm XGBoost[53] (Python Package V0.90). Briefly, the first stage of the GBT method is to generate an initial prediction of the target variable for each training example. In successive stages, GBT iteratively generates a series of decision trees. After each tree, the current prediction for any given training example is the initial prediction added to the sum of the outputs for all trees generated thus far. Each tree in the series is optimized to yield incremental outputs that, when added to the sum of previous outputs, tend to minimize the loss function (prediction error). The incremental value is determined by approximating both the first and second order gradient of the loss function at the current prediction stage. Optimization of each tree is accomplished by successively selecting features that split training examples into smaller groups (or nodes). The splitting feature is selected such that a single incremental output value assigned to each subgroup can most closely approximate the set of best incremental output values. For each training set (core or add-on set), each of several quality-informative properties was used as input to a logistic function that assigned a weight to each training variant within the set. The parameters of each logistic function were treated as hyperparameters that were optimized for performance (see details in the Bayesian optimization of hyperparameters section below). Subsequently, the weights for all positive training examples were scaled by a factor such that the total of re-scaled weights for positive training examples was equal to the total weight of negative examples.

Moving window analysis to identify quality-informative variant properties

To identify the quality-informative properties used as described above for variant weighting, candidate properties were evaluated by moving window analysis. Candidate informative properties included: allele frequency (for all training sets except the ones from MAVE studies[6–10]), "review stars" (for ClinVar[44] training sets), "number of homozygotes" (for gnomAD[47] training sets), "label confidence," and "mutational accessibility" (the latter two are candidates for MAVE training sets[6–10]). The "label confidence" score for each variant was defined to be either the MAVE score itself (for scores < 0.5) or $1 -$ score (for scores > 0.5). Use of the mutational accessibility property was motivated by the fact that, although variants with more than one nucleotide change in a given codon can be generated by mutagenesis strategies used in variant effect mapping, these rarely occur in humans. The mutational accessibility of each possible amino acid change was the total probability of all possible single-nucleotide change events yielding that amino acid change, where the probability that a single nucleotide change event occurs in each codon was defined via the human codon usage frequency for that codon divided by nine (because nine single-nucleotide edits are possible for any given codon). Amino acid changes requiring more than one single-nucleotide change within the same codon received a mutational accessibility score of 0.

For each proposed candidate property, we carried out moving window analysis to evaluate whether model performance depends on the value of the property. To this end, add-on set examples were first ordered by the property. We then defined N_W moving windows, each containing a fraction F_E of the total X add-on training examples and incrementing the position of successive windows every $I = [X * (1 - F_E)/(N_W - 1)]$ training examples. Where the number of examples is not evenly divisible by I, the final window may contain slightly fewer than $X * F_E$ examples. Here, we used $N_W = 100$ and $F_E = 0.5$.

To evaluate the data quality of each window, we estimated model performance (AUBPRC) on the core set by using 10-fold cross-validation via XGBoost[53] (with default algorithm level hyperparameters) where the training examples in each fold were supplemented by add-on examples in that moving window. To assess whether a property was informative, we calculated correlation between moving window performance and window number for each candidate informative property and also for each of ten random orderings of add-on examples. From this, we calculated a *Z* score for the observed correlation relative to the distribution of randomly ordered correlations and considered a property to be informative if it exhibited a *Z* score above 1 or less than -1. The negative and positive sign of a *Z* score indicates whether there was a negative or positive correlation between performance of moving windows and the property. Although moving window analysis combined one or two add-on sets at a time with the core set, we note that add-on sets may provide a contribution that is greater or lower than that observed here when they are used in conjunction with many other training sets during hyperparameter tuning. Therefore, all training sets and their associated informative properties (Table S3) were included for the weighting process that was optimized during hyperparameter tuning, even for *Z* scores exhibiting modest departures from zero.

Bayesian optimization of hyperparameters

Two types of hyperparameters were employed for VARITY models. First, there were algorithm-level hyperparameters such as number of trees, tree depth, and learning rate (see Table S4 for a full list). Second, there were hyperparameters in the logistic functions that were used for assigning weights to different training sets (see Table S3 for full list). There are three parameters in each logistic function, namely the maximum weight L, the midpoint x_0, and the growth rate k. Each logistic function takes one quality informative property as input. For the training set that has more than one associated quality-informative property, the weights were assigned as the product of multiple logistic functions.

All hyperparameters were optimized simultaneously by Bayesian optimization with HyperOpt[54–56] (Python package V0.2.2). This process involves random initialization of hyperparameter values (with uniform prior) followed by iteratively repeated trials (here we used 300 trials) with alternative hyperparameter values suggested on the basis of an expected improvement measure calculated via a probability distribution estimated from the performance of previously tried hyperparameter settings. For each trial, the hyperparameter set was evaluated via model performance on the core training set estimated in 10-fold cross-validation where the core training examples in each fold were supplemented with add-on training sets examples and all training examples were weighted with hyperparameter values suggested on the current trial. The metric used here for model performance (the objective function for hyperparameter optimization) was the mean AUBPRC on validation sets (average over ten validation sets).

To limit model overfitting that can arise from repeated interrogation of the evaluation set with different hyperparameter settings, we used the following procedure to automatically determine the final hyperparameter values: (1) re-order all trials from HyperOpt[54–56] by mean AUBPRC on training sets (averaged over ten training sets) from low to high, (2) calculate a moving window (we used window size 30 for total 300 trials) average of mean AUBPRC on validation sets, (3) define an "early stopping" point at the first moving window (the "fittest" region) for which mean AUBPRC on validation sets begins to descend, and (4) select as final the hyperparameters from the trial within this "fittest" region that achieved the highest mean AUBPRC on validation sets.

Performance evaluation with independent test sets and nested cross-validation

To obtain an independent test dataset based on *de novo* variation in neurodevelopmental disorders, we downloaded all *de novo* variants from denovo-db 1.61[57] (download date 2020-10-20) that corresponded to neurodevelopmental disorder case/control studies. We extracted the subset of rare missense variants (MAF $<$ 0.5%) from 253 candidate neurodevelopmental disease genes for which variation was enriched in neurodevelopmental disease patients.[58] For the purpose of performance evaluation, the variants that appeared in "control" and "case" populations were labeled as negative (putatively benign) and positive (putatively pathogenic), respectively. Because a subset of variants annotated in HGMD[46] had been used to train computational methods against which we wished to compare our results, variants annotated in HGMD[46] (version 2020) were removed, as were variants used in VARITY_R and VARITY_ER model training. There are in total 367 *de novo* missense variants (321 putatively pathogenic and 46 putatively benign) left for comparison. Considering only variants scored by each of the 23 computational methods to be compared, the final validation dataset contained 215 *de novo* missense variants (188 putatively pathogenic and 27 putatively benign). For the validation analysis with published variant effect maps, we assembled map scores for a subset of six proteins associated with human genetic disease: CALM1 (MIM: 114180), TPK1 (MIM: 606370), CBS (MIM: 613381), PTEN (MIM: 601728), BRCA1 (MIM: 113705), and VKORC1 (MIM: 608547).

To evaluate the generalization performance of VARITY models more broadly, we adopted 10-fold nested cross-validation strategy. For each outer loop, the hyperparameters were determined via Bayesian optimization[54–56] based on inner-loop 10-fold cross-validation. The optimized hyperparameters were then used for training based on all data in the outer-loop training set. Performance on VARITY models (and other computational approaches) were assessed on the ten held-out outer-loop validation sets (Figure S2).

To compare the performance of VARITY models to previously developed pathogenicity predictors, we collected the scores from 25 predictors. For 22 of these, scores were collected from dbNSFP (V4.0b2)[48], while scores for two predictors (MPC[41] and EVMutation[27]) were assembled from links provided by the corresponding papers and scores for DeepSequence[42] were collected from an independent benchmarking study of 11 proteins:[54] UBE2I (MIM: 601661), TPMT (MIM: 187680), TPK1 (MIM: 606370), TP53 (MIM: 191170), SUMO1 (MIM: 601912), PTEN (MIM: 601728), MAPK1 (MIM: 176948), HRAS (MIM: 190020), CALM1 (MIM: 114180), BRCA1 (MIM: 113705), and ADRB2 (MIM: 109690).

Feature contribution to output score and model performance

For each variant, the VARITY model provides a log(odds) ("lod") score (before subsequent transformation to the final score via a sigmoid transformation), which is more positive for variants that are inferred to be pathogenic and more negative for variants that are inferred to be benign. To provide intuition about which features of a given variant drove its score, we defined an "output contribution" score by using Shapley additive explanation values[59,60] retrieved from the XGBoost[53] output (with option "predict_pred_interactions" turned on).

It can be useful to estimate the contribution of individual feature groups or pairs of feature groups to a model's performance (as opposed to a model's output values). We therefore defined a "performance contribution" score for all features for each variant used in training. For a positive (putatively pathogenic) variant used in training, performance contribution was the output contribution multiplied by the weight given to the variant during hyperparameter tuning. For a negative (putatively benign) variant used in training, each feature performance contribution is obtained by negating the output contribution and again multiplying by the weight used for that variant. Thus, output contributions moving in the right direction are counted as beneficial to model performance.

Results

Developing a machine-learning and data-weighting strategy

We first assembled a large resource of input features that are potentially informative about variant function for all

possible missense variants in ~18,000 human proteins. To limit the circularity that may inflate the model performance and to limit the effects of training set bias[61], we excluded features informed by variant annotation (e.g., scores predicted by supervised models) or protein identity (Table S1). For model training and performance evaluation, we next identified from ~4,000 human disease proteins a "core" set of variants with high quality annotation from ClinVar[44]. This set was limited to rare variants (MAF < 0.5%) for the VARITY_R model. To assess sensitivity of our results to this threshold, we also defined a core set of extremely rare variants (MAF < 10^{-6}) to train and test a VARITY_ER model. For the core set, we labeled variants as "positive" (putatively pathogenic) or "negative" (putatively benign) on the basis of pathogenicity interpretations. We then collected various "add-on" variants, employing potentially less-reliable information about whether non-core variants should be labeled as positive or negative (see material and methods). Add-on variants were drawn from gnomAD,[47] HGMD,[46] HumsaVar,[45] ClinVar[44] (excluding the core set variants), and a handful of multiplexed assays of variant effect (MAVE) studies[6–10] (Table S2). Add-on variants were grouped into add-on training sets based on source (e.g., gnomAD[47]), label (e.g., "negative"), and rarity (e.g., "MAF > 0.5%") so that each add-on set can be employed separately with distinct effects on training VARITY models (see material and methods; Table S3).

Although VARITY employs a "workhorse" machine-learning method (the gradient boosted tree algorithm XGBoost[53]) (see material and methods), it is distinct from previous machine-learning strategies applied to variant impact prediction. Distinct *a priori* weights were assigned to different training examples, and these weights were considered in the loss function used for tree optimization. For each training set (core or add-on set), one or more quality-informative properties were used as inputs to a logistic function that assigned weights to each variant. The parameters of each logistic function were optimized as hyperparameters (see material and methods; Figure 1).

Quality-informative properties of each training set

To identify properties related to the utility of different add-on sets for inferring pathogenicity, we ordered the examples in an add-on set (or logically related pairs of add-on sets) by candidate property and carried out a moving window analysis (see material and methods), To evaluate the predictive utility of each window, we estimated the model performance by using 10-fold cross-validation on the core training set where the training examples in each fold were supplemented by all of the add-on examples in that moving window. When a candidate property correlated with moving window predictive utility in an add-on set, this property was identified as an informative property for the add-on set. For example, a moving window analysis of putatively benign variants from gnomAD[47] showed variants with both lower and higher allele frequency to be less useful in training VARITY_R (Figure 2). This agreed with our expectation that variants with lower allele frequency are more likely to be "contaminated" with damaging variants and that common variants might not be representative of the rare variants used for testing. Allele frequency was similarly identified as a quality-informative property for negative-labeled ClinVar[44] and HumsaVar[45] variants. For several positive-labeled (putatively pathogenic) add-on sets, variants with higher allele frequency were found to be less useful. We also identified both label confidence and mutational accessibility properties (see material and methods for definitions) as quality-informative properties for variants with MAVE scores (Figure S3; Table S3). Although the core training set was considered high quality, we considered ClinVar[44] review stars as a quality-informative property to further weight these training variants.

Bayesian optimization on hyperparameters

The parameters of the variant-weighting logistic function used for each training set were treated as hyperparameters subject to optimization, as were algorithm-level hyperparameters such as learning rate, number of trees, and the maximum depth of a single tree (Table S3 and Table S4). All hyperparameters were tuned simultaneously with Bayesian optimization,[54–56] which consists of many consecutive trials, each making use of a different set of hyperparameter values suggested by the previous trials. For each trial, the hyperparameter set was evaluated via VARITY model performance on the core set examples in 10-fold cross-validation where the core training examples in each fold were supplemented with add-on training sets examples and all training examples were weighted with hyperparameter values suggested on the current trial. To avoid overfitting to the validation set during hyperparameter tuning, we selected a hyperparameter set by using a scheme akin to "early stopping" (see material and methods; Figure S4). With thus-optimized hyperparameters, all 34,087 core set variants were given high weight ($\geq$70%). While nearly all (123,591 of 123,621) add-on set variants were given non-zero weights for VARITY_R, only 1.8% of add-on variants received $\geq$70% weight (see Figure 3 for the optimized weights of gnomAD[47] add-on sets and Figure S5 for all training sets). The effective number of training examples (the sum of weights of all core and add-on set examples) for VARITY_R was 41,898 (24,842 positive examples and 17,056 negative examples) as compared with the 157,708 total of core and add-on examples. A subsequent reweighting step equalized the effective number of positive and negative examples.

An alternative VARITY_ER model optimized for extremely rare variants

Although the threshold that we used to define rare variants (MAF < 0.5%) in the VARITY_R model is commonly used,[1] we considered the possibility that moderately rare variants (10^{-6} < MAF < 0.5%) may not be representative of extremely rare variants (MAF < 10^{-6}, for which no allele

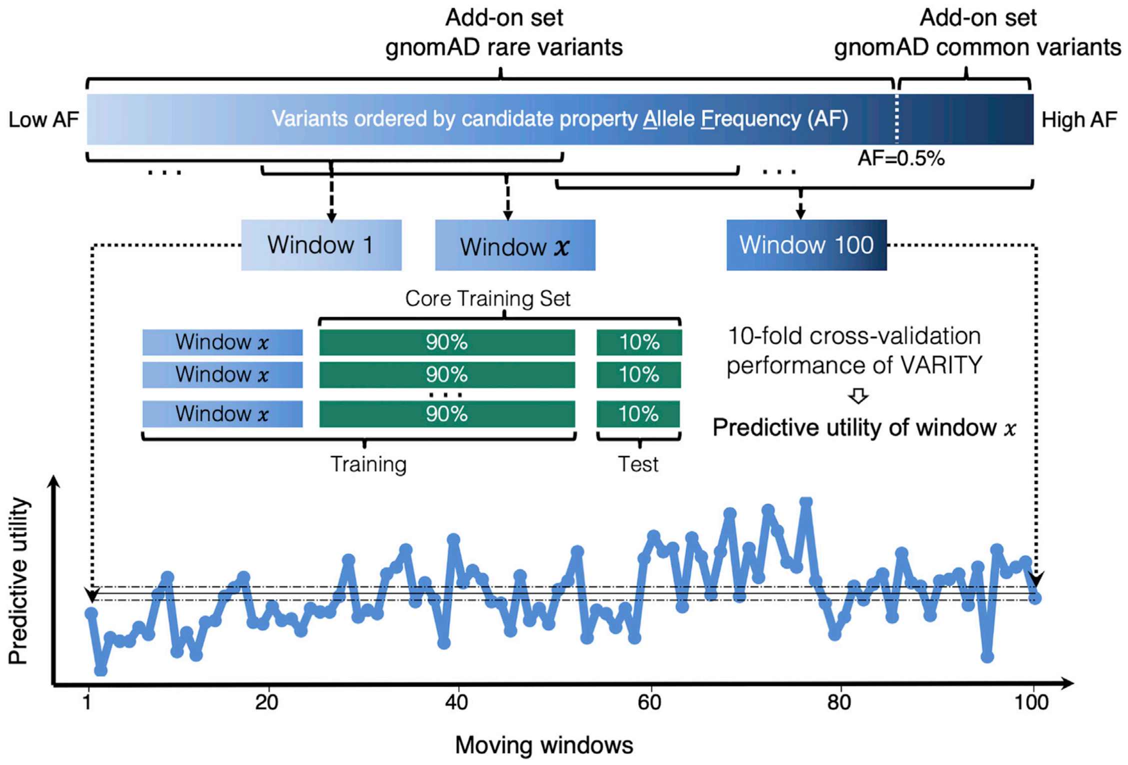

Figure 2. Identifying properties informative of predictive utility with moving window analysis
Here, we illustrate evaluation of whether the allele frequency property can identify less useful subsets of two add-on variant sets (common and rare gnomAD[47] variants). Variants from both common and rare add-on gnomAD[47] sets were ordered from low to high allele frequency, and a series of moving windows (each capturing the same number of examples) was generated. To evaluate the predictive utility of each window, we estimated the model performance on the core set by using 10-fold cross validation where the training examples in each fold were supplemented by examples in that moving window. One solid and two dashed horizontal black lines indicate the mean ± standard error of the predictive utility of all moving windows. Here, moving window analysis showed that allele frequency correlated with moving window predictive utility, and lower and higher allele frequency gnomAD[47] variants exhibited less utility as a source of negative variants for VARITY_R performance.

count was detected in gnomAD[47]). We therefore developed an alternative VARITY_ER model by using only extremely rare variants from ClinVar[44] as the core set (but including moderately rare ClinVar[44] variants as add-on variants). Like VARITY_R, VARITY_ER modeling proceeded via moving window analysis and hyperparameter tuning, and all 18,792 core set variants and 133,848 out of 138,916 add-on set variants received non-zero weights. The effective number of training examples was 24,074 for VARITY_ER, well below the total of 157,708 training examples considered (Figure S6, Figure S7, and Figure S8).

Leave-one-variant-out (LOO) prediction for ∼18,000 human proteins

The final VARITY_R and VARITY_ER models were each trained with both weighted core and add-on sets with optimized hyperparameters and were each used for inference of functional impact for all possible missense variants for each of ∼18,000 human proteins. We observed that cross-validation performance for VARITY_R and VARITY_ER models was only slightly higher for the training set than for the validation sets using the optimized hyperparameter settings (See Figure S4 and Figure S7), suggesting that overfitting, if any, was modest. However, to avoid inflating performance estimates as a result of overfitting, we applied a leave-one-variant-out (LOO) strategy such that each variant used (i.e., having non-zero weight) in training was excluded in turn from the VARITY_R (or VARITY_ER) model used to score that variant, yielding a set of VARITY_R_LOO (or VARITY_ER_LOO) scores.

Extracting intuition from VARITY models

To better understand the rationale for each VARITY score, we used Shapley additive explanation values[59,60] to assess the contributions made by features and their pairwise combinations. For each variant, contributions were estimated both to VARITY score and, if the variant was used in training, to model performance (see material and methods). For each feature group (Table S1), we estimated the total contribution (including independent and pairwise contribution) to model performance as weighted average of the contribution from all training examples by using weight of each training example that was optimized during hyperparameter tuning (Figure 4). Contributions to model performance at the level of individual features and feature pairs were also evaluated (Figure S9).

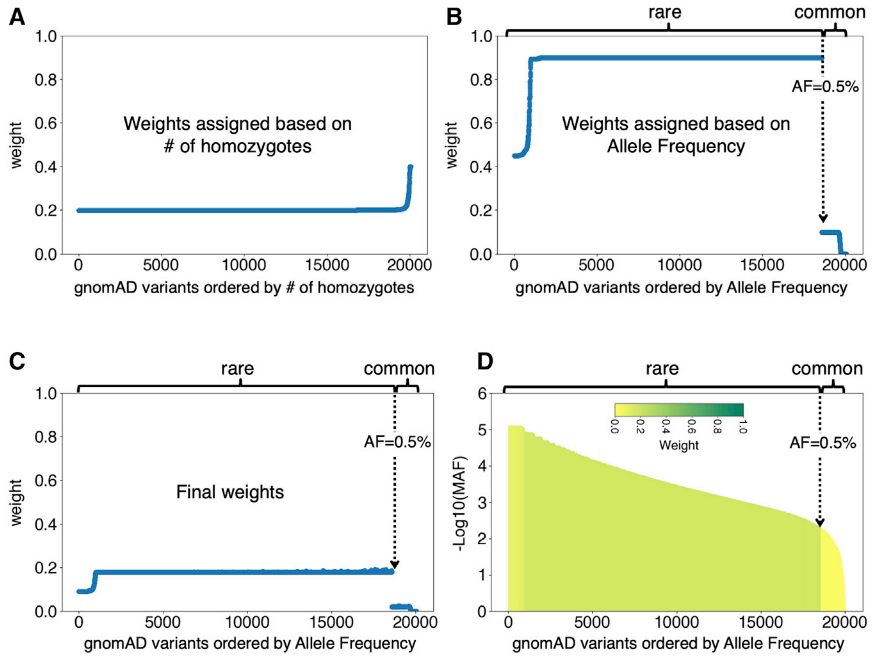

Figure 3. Assigning weights to variants in training add-on sets
Here, we illustrate weight assignment to variants in common and rare gnomAD[47] add-sets of negative (putatively benign) training examples based on the allele frequency and number of homozygotes observed in gnomAD.
(A) Weights of all gnomAD[47] variants (the union of the rare and common gnomAD[47] add-on sets) assigned via logistic function that takes number of homozygotes as an input.
(B) Weights of variants from either rare or common gnomAD[47] add-on set assigned via logistic function that takes allele frequency as an input.
(C) The final weights of gnomAD[47] variants by taking the product of the weights assigned by (A) and (B) for each variant.
(D) Another view of the final weights of gnomAD[47] variants with −log10(allele frequency) as the y axis and showing the final weights by color.

For the VARITY_R model, we found that conservation-based features were the most important for model performance, which is perhaps unsurprising given that they are foundational to most computational predictors of variant function. Among these, Provean[24], SIFT,[25,26] EVMutation,[27] and LRT[29] provided the greatest contribution. The next most important feature group was "delta AA properties," a set of features capturing differences in physicochemical properties between the missense and wild-type amino acid. Among these, the "delta cyclic property" (capturing whether a proline residue has been substituted for a non-proline residue or vice versa) was the most important. The "delta sulfur property" (capturing whether a cysteine or methionine residue has been substituted for a non-sulfur-containing residue or vice versa) and the "delta hydropathy index" were also very helpful. Next were features related to "surface-accessible area," representing the extent to which the amino acid is exposed to solvent, and "PPI," a group of features modeling the extent to which the environment of an amino acid changes upon protein-protein interaction (see material and methods).

Features and feature groups will of course combine to contribute to VARITY models in a non-additive way. For example, the "IN/OUT Pfam domain" feature (capturing whether an amino acid position falls within a conserved protein domain), which is derived from multiple sequence alignments, contributed to the performance of the "conservation scores" feature group. It is therefore understandable that this feature group masks the predictive value of "IN/OUT Pfam domain" (as indicated by negative pairwise performance contribution values for VARITY_R in Figure 4). A negative pairwise performance contribution for individual features was also observed among nine conservation scores. Because these conservation scores are similar, partial redundancy with one another was to be expected (see Figure S9). We also have observed that the combination of "conservation scores" with other feature groups such as "delta AA properties" and "PPI" provides a positive (synergistically favorable) pairwise feature contribution to model performance. Features contributing to each VARITY_ER model prediction score were also identified, and the performance contributions of individual and grouped features was again analyzed with the weighted training examples, yielding similar results (Figure S10 and Figure S11).

Assessing VARITY with *de novo* variants in neurodevelopmental disorder studies

To assess VARITY, we examined missense variants from neurodevelopmental case/control studies, focusing on 253 genes enriched for *de novo* variation in neurodevelopmental disease patients.[57,58] For performance comparison, we considered all rare *de novo* variants in these genes that had not been used in training by VARITY and were not annotated by HGMD[46] (see material and methods). After retaining only variants that had MAF < 0.5% and that had been scored by all 23 of the methods to be compared, there were 188 variants in the case group and 27 in the control group.

We wished to judge performance using both receiver operating characteristics (ROC) and precision versus recall analysis. Precision versus recall analysis has the advantage of being more intuitively related to the needs of most users. However, precision varies as a function of the prior probability, and this prior is by default determined by the fraction of test examples with a Positive (putatively pathogenic) label. Moreover, this default prior may not have any relationship with the prior probability that is most relevant to a clinical geneticist using our output. To enable

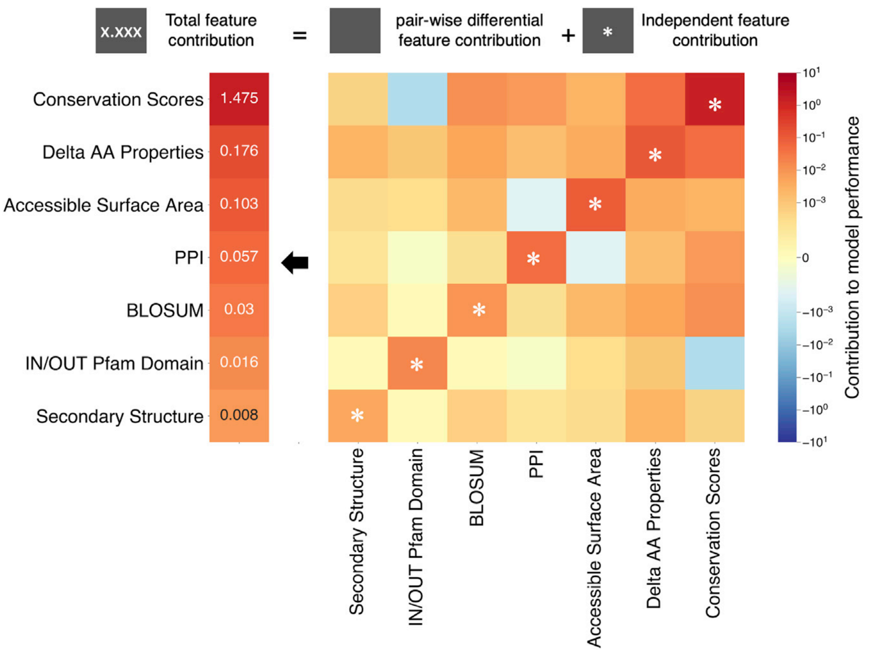

Figure 4. Feature group contribution to model performance for VARITY_R model
The contribution of each feature group to VARITY_R model performance was averaged (weighted) across all training examples via the weight of each training example as optimized during hyperparameter tuning. The first column (left) indicates the total contribution to model performance of each feature group. For each feature group, the total contribution can be decomposed into the individual feature contribution (matrix cell with a star symbol on the corresponding row) and the differential contribution of that feature when it is combined with each other feature group (matrix cells without a star symbol on the corresponding row). Red and blue color indicates positive and negative contribution to model performance, respectively. A blue-colored cell for pairwise differential feature contribution indicates there is a certain amount of redundancy between two feature groups (e.g., between conservation scores and IN/OUT Pfam domain).

greater intuition and fairer comparisons, we therefore transformed each empirical precision-recall curve to the curve corresponding to a balanced (50% probability of pathogenicity) prior. Thus, our performance measures were as follows: area under the ROC curve (AUROC), area under the balanced precision recall curve (AUBPRC), and recall at 90% balanced precision (R90BP; see material and methods for details). For this *de novo* variant application, we used the case/control status of the patient(s) in which each variant appears as a proxy for pathogenicity.

VARITY_ER numerically outperformed all other methods in terms of AUBPRC. When performance was measured with the subset of variants that had been scored by all methods, AUBPRC performance improvement achieved statistical significance ($p < 0.05$) relative to every method except MPC[41] ($p = 0.125$) and M-CAP[17] ($p = 0.099$). Moreover, when we used larger variant sets that had been scored by MPC[41] (285 variants in case group and 38 variants in control group) or M-CAP[17] (320 variants in case group and 45 variants in control group), VARITY_ER's performance improvement was statistically significant ($p =$ 0.048 and 0.029 for MPC[41] and M-CAP[17], respectively). When the score threshold for every method was tuned to achieve 90% balanced precision, VARITY_ER recovered 10% more (presumed pathogenic) variants from the case group than the closest non-VARITY method. VARITY_ER outperformed VARITY_R with a 2% greater AUBPRC ($p =$ 0.122), which may be explained by the fact that most *de novo* missense variants are extremely rare. However, VARITY_R also numerically outperformed all other methods (see Figure 5 and Table S5 for AUBPRC comparison; Figure S12 and Table S6 for AUROC comparison; Table S7 for individual comparisons with MPC[41] and M-CAP[17]).

Assessing VARITY with experimental variant effect maps

As another form of independent validation, we wished to assess agreement between impact scores and systematic experimental assessments of variant function. We therefore evaluated the ability of scores from each computational method to predict the quantitative scores from each of six recently published missense variant effect maps on human disease related genes.[6–11] Because five out of six maps (all except *VKORC1* map) had been indirectly used in VARITY training, we included leave-one-variant-out scores (VARITY_R_LOO and VARITY_ER_LOO) for comparison. VARITY_ER_LOO showed best correspondence in terms of Pearson correlation coefficient (PCC) with variant effect map scores ($PCC = 0.405$), significantly outperforming all other non-VARITY predictors ($p < 0.05$; Figure 6 and Table S8 for PCC results; Table S9 for Spearman's rank correlation coefficient [SRC] results).

More broadly assessing VARITY performance via nested cross-validation

To further compare generalization performance of VARITY models with previously developed computational predictors of missense variant effects, we used the high-quality core sets from ClinVar.[44] To avoid possible circularity from model training and hyperparameter tuning, we adopted a nested cross-validation strategy (see material and methods).

VARITY_R significantly outperformed all 23 methods on rare variants from ClinVar[44] (MAF < 0.5%; VARITY_R core sets) by all criteria (i.e., AUBPRC, R90BP, and AUROC; $p <$ 0.05 via one-sided paired t test), recovering 13% more pathogenic rare missense variants at 90% balanced precision than the next-best method, REVEL[16] (Figure 7 and Table S10 for AUBPRC comparison; Figure S13 and Table S11

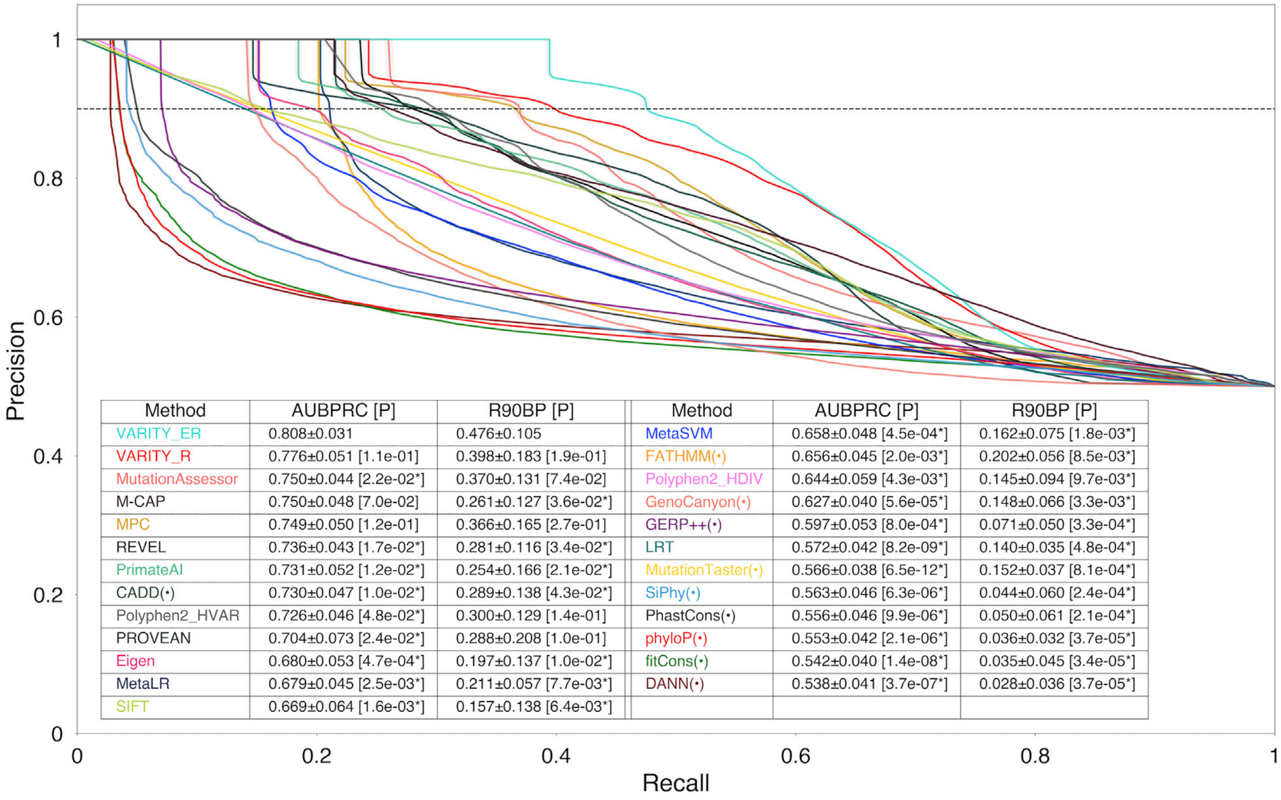

Method	AUBPRC [P]	R90BP [P]	Method	AUBPRC [P]	R90BP [P]
VARITY_ER	0.808±0.031	0.476±0.105	MetaSVM	0.658±0.048 [4.5e-04*]	0.162±0.075 [1.8e-03*]
VARITY_R	0.776±0.051 [1.1e-01]	0.398±0.183 [1.9e-01]	FATHMM(•)	0.656±0.045 [2.0e-03*]	0.202±0.056 [8.5e-03*]
MutationAssessor	0.750±0.044 [2.2e-02*]	0.370±0.131 [7.4e-02]	Polyphen2_HDIV	0.644±0.059 [4.3e-03*]	0.145±0.094 [9.7e-03*]
M-CAP	0.750±0.048 [7.0e-02]	0.261±0.127 [3.6e-02*]	GenoCanyon(•)	0.627±0.040 [5.6e-05*]	0.148±0.066 [3.3e-03*]
MPC	0.749±0.050 [1.2e-01]	0.366±0.165 [2.7e-01]	GERP++(•)	0.597±0.053 [8.0e-04*]	0.071±0.050 [3.3e-04*]
REVEL	0.736±0.043 [1.7e-02*]	0.281±0.116 [3.4e-02*]	LRT	0.572±0.042 [8.2e-09*]	0.140±0.035 [4.8e-04*]
PrimateAI	0.731±0.052 [1.2e-02*]	0.254±0.166 [2.1e-02*]	MutationTaster(•)	0.566±0.038 [6.5e-12*]	0.152±0.037 [8.1e-04*]
CADD(•)	0.730±0.047 [1.0e-02*]	0.289±0.138 [4.3e-02*]	SiPhy(•)	0.563±0.046 [6.3e-06*]	0.044±0.060 [2.4e-04*]
Polyphen2_HVAR	0.726±0.046 [4.8e-02*]	0.300±0.129 [1.4e-01]	PhastCons(•)	0.556±0.046 [9.9e-06*]	0.050±0.061 [2.1e-04*]
PROVEAN	0.704±0.073 [2.4e-02*]	0.288±0.208 [1.0e-01]	phyloP(•)	0.553±0.042 [2.1e-06*]	0.036±0.032 [3.7e-05*]
Eigen	0.680±0.053 [4.7e-04*]	0.197±0.137 [1.0e-02*]	fitCons(•)	0.542±0.040 [1.4e-08*]	0.035±0.045 [3.4e-05*]
MetaLR	0.679±0.045 [2.5e-03*]	0.211±0.057 [7.7e-03*]	DANN(•)	0.538±0.041 [3.7e-07*]	0.028±0.036 [3.7e-05*]
SIFT	0.669±0.064 [1.6e-03*]	0.157±0.138 [6.4e-03*]			

Figure 5. Assessing VARITY balanced precision recall performance for *de novo* variants in neurodevelopmental disorder studies
Here, we show balanced precision versus recall curves for VARITY_ER, VARITY_R, and 23 other variant pathogenicity predictors. Predictors designed specifically for nucleotide variants are indicated with a "(•)." Here, the test set was a total 215 rare *de novo* variants (188 positive and 27 negative examples, see material and methods). Recall was averaged over 2,000 bootstrapped test sets with standard error indicated by the surrounding gray region. As overall performance measures, AUBPRC and R90BP (the black dotted line) and their standard errors are shown. Statistical significance relative to VARITY_ER applied a one-sided *Z* test based on 2,000 bootstrapped test sets (p values in brackets were indicated with a "*" where $p < 0.05$). Other test statistics, such as 95% confidence interval and effect size, can be found in Table S5. When individual comparison used a larger variant set that had been scored by MPC[41] (285 variants in case group and 38 variants in control group) or M-CAP[17] (320 variants in case group and 45 variants in control group), VARITY_ER's improved AUBPRC performance was statistically significantly ($p = 0.048$ and 0.029 for MPC[41] and M-CAP,[17] respectively; Table S7).

for ROC comparison). EVMutation[27] and DeepSequence[42] had few scores available for core set variants and, because we limited the overall comparison to variants assessed by all methods, their inclusion would have dramatically reduced the space of test variants. However, direct comparison of VARITY_R with each method with the intersection of test variants scored by each respective method showed that VARITY_R significantly outperformed both methods (Table S12). Methods designed to evaluate amino acid substitutions generally outperformed methods that were designed for nucleotide-level changes (see predictors marked with "(•)" in Figure 7). Indeed, among nucleotide-level methods, only CADD[18] (which may have benefited from amino-acid-level PolyPhen2[19,20] scores as training features) was able to outperform an amino-acid-level method.

To evaluate the extent to which VARITY's success depended on the variant-weighting scheme, we retrained a "VARITY_R_unweighted" model, giving equal weight to all core and add-on variants. This reduced AUBPRC performance from 92% to 90.9% ($p = 6.5 \times 10^{-5}$; Student's t test comparing the two sets of outer-loop validation performance values). We expected that performance for the no-variant-weighting version of VARITY would fall further as more add-on training examples with uncertain quality are included. To evaluate this, we included additional gnomAD[47] variants that were originally excluded from the putatively-benign add-on training set because they had never been seen in a homozygous individual. After including these variants in training, the nested cross-validation AUBPRC performance of VARITY_R_unweighted was reduced further from 90.9% to 89.9% ($p = 2.1 \times 10^{-4}$).

We also evaluated VARITY_ER performance by using the core set of extremely rare variants from ClinVar[44] (MAF < 10^{-6}; VARITY_ER core sets), again with 10-fold nested cross-validation. VARITY_ER outperformed all 23 methods by all criteria ($p < 0.05$ via one-sided paired t test), recovering 12% more pathogenic rare missense variants at 90% balanced precision than the next-best method REVEL[16] (Figure S14 and Table S13 for AUBPRC comparison; Figure S15 and Table S14 for ROC comparison). Although EVMutation[27] and DeepSequence[42] were again excluded, VARITY_ER outperformed EVMutation[27] in a

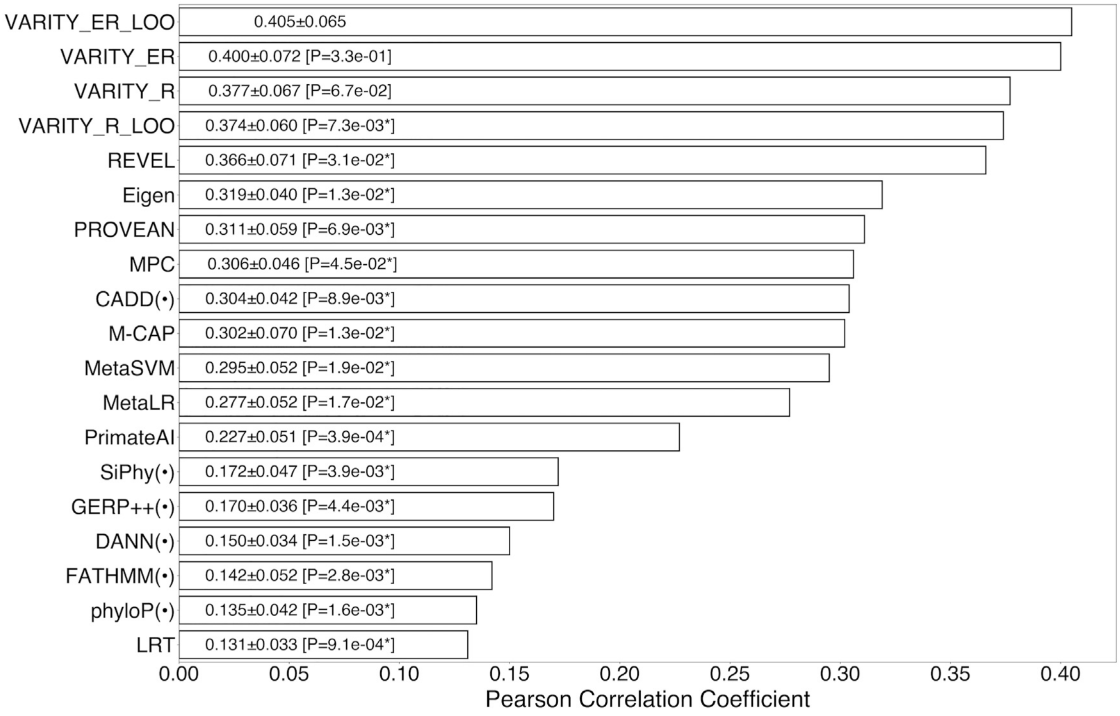

Figure 6. Performance of VARITY_R model and other predictors on variant effect maps
Evaluation of VARITY models (VARITY_R and VARITY_ER) and 19 other computational predictors according to correlation (Pearson correlation coefficient or PCC) with variant function scores of rare (MAF < 0.5%) missense variants from six experimental variant effect maps.
SIFT,[25,26] PolyPhen2_HVAR,[19,20] PolyPhen2_HDIV,[19,20] and MutationAccessor[33] were not included because we did not have their scores for all six variant effect maps. Because some of the variant effect maps were indirectly used in VARITY training, we included VARITY_R_LOO and VARITY_ER_LOO for comparison. VARITY_ER_LOO had the overall best performance and was significantly better than all other models. Standard error of PCC was derived from the set of PCC values from each variant effect map. Statistical significance of each predictor's performance was evaluated relative to VARITY_ER_LOO via a one-sided paired t test with 5 degrees of freedom (p values in brackets were indicated with a "*" where $p < 0.05$). For compactness, four methods that have correlation less than 0.1 are not shown (see Table S8). Other information, such as 95% confidence intervals and differences in PCC values, is in Table S8. The PCC comparison for each individual variant effect map is also in Table S8.

pairwise comparison based on extremely rare core set variants with available EVMutation[27] scores (Table S15). Only 16 extremely rare benign core set variants had DeepSequence[42] scores, leaving too few variants in each held-out subset during 10-fold nested cross-validation to accurately evaluate performance.

Discussion

Evidence to evaluate variant pathogenicity is typically less available for rare as opposed to common variants. Detecting population-level correlation between a trait and a genotype has reduced power at lower minor allele frequencies and is futile for extremely rare variants. Experimental functional evidence is also less available for lower-allele-frequency variants. For these reasons, and because the majority of unique clinically observed variants are rare, there is a particularly strong motivation to improve computational methods for inferring the pathogenicity of rare variants.

Here, we developed predictors of variant pathogenicity that were specifically optimized for performance on rare and extremely rare missense variants. Given previous evidence that common variants are not representative of rare variants in the context of training pathogenicity models,[16] perhaps the primary advantage of VARITY models is that rare variants have been given greater weight in training. Other key advantages are that VARITY makes use of large sets of potentially biased or error-prone training data while optimizing the potential for these add-on variant sets to increase performance. VARITY models benefitted not only from the process of filtering and weighting add-on examples but also from the use of features that have, either individually or in combination, not been used previously. To limit protein-specific bias and circularity, VARITY models also excluded features that are informative about protein identity or that may have been informed by previous pathogenicity annotations.

VARITY models performed well when evaluated via an independent set of *de novo* missense variants collected from neurodevelopmental case-control studies. Here, we only considered the subset of *de novo* missense variants that were in genes previously reported as being enriched for *de novo* missense and/or likely-gene-disruptive variants in neurodevelopmental disease cases. Because this certainly excluded some disease-relevant genes/variants,

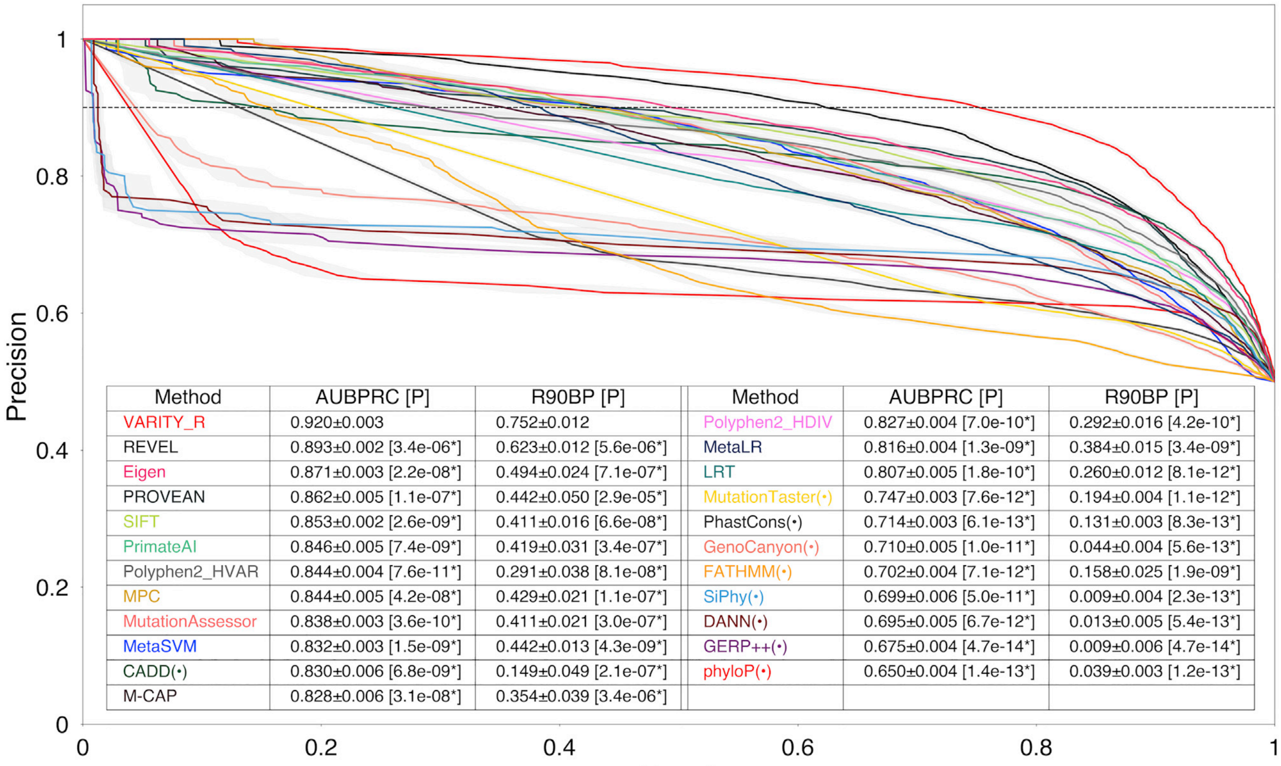

Method	AUBPRC [P]	R90BP [P]	Method	AUBPRC [P]	R90BP [P]
VARITY_R	0.920±0.003	0.752±0.012	Polyphen2_HDIV	0.827±0.004 [7.0e-10*]	0.292±0.016 [4.2e-10*]
REVEL	0.893±0.002 [3.4e-06*]	0.623±0.012 [5.6e-06*]	MetaLR	0.816±0.004 [1.3e-09*]	0.384±0.015 [3.4e-09*]
Eigen	0.871±0.003 [2.2e-08*]	0.494±0.024 [7.1e-07*]	LRT	0.807±0.005 [1.8e-10*]	0.260±0.012 [8.1e-12*]
PROVEAN	0.862±0.005 [1.1e-07*]	0.442±0.050 [2.9e-05*]	MutationTaster(•)	0.747±0.003 [7.6e-12*]	0.194±0.004 [1.1e-12*]
SIFT	0.853±0.002 [2.6e-09*]	0.411±0.016 [6.6e-08*]	PhastCons(•)	0.714±0.003 [6.1e-13*]	0.131±0.003 [8.3e-13*]
PrimateAI	0.846±0.005 [7.4e-09*]	0.419±0.031 [3.4e-07*]	GenoCanyon(•)	0.710±0.005 [1.0e-11*]	0.044±0.004 [5.6e-13*]
Polyphen2_HVAR	0.844±0.004 [7.6e-11*]	0.291±0.038 [8.1e-08*]	FATHMM(•)	0.702±0.004 [7.1e-12*]	0.158±0.025 [1.9e-09*]
MPC	0.844±0.005 [4.2e-08*]	0.429±0.021 [1.1e-07*]	SiPhy(•)	0.699±0.006 [5.0e-11*]	0.009±0.004 [2.3e-13*]
MutationAssessor	0.838±0.003 [3.6e-10*]	0.411±0.021 [3.0e-07*]	DANN(•)	0.695±0.005 [6.7e-12*]	0.013±0.005 [5.4e-13*]
MetaSVM	0.832±0.003 [1.5e-09*]	0.442±0.013 [4.3e-09*]	GERP++(•)	0.675±0.004 [4.7e-14*]	0.009±0.006 [4.7e-14*]
CADD(•)	0.830±0.006 [6.8e-09*]	0.149±0.049 [2.1e-07*]	phyloP(•)	0.650±0.004 [1.4e-13*]	0.039±0.003 [1.2e-13*]
M-CAP	0.828±0.006 [3.1e-08*]	0.354±0.039 [3.4e-06*]			

Figure 7. Comparing balanced precision recall performance of VARITY_R with other predictors in predicting a high-quality "core" variant set (MAF < 0.5%)
We compare balanced precision versus recall performance for VARITY_R (with nested cross-validation) with 23 other variant pathogenicity predictors. For compactness, one predictor with AUBPRC < 0.6 is not shown (See Table S10). Predictors that had been designed specifically for nucleotide variants are indicated with a "(●)." The test set was 9,719 variants (5,912 positive and 3,807 negative examples) from the core set, after removing variants annotated by HGMD[46] and retaining only variants that had been scored by all methods. Recall was averaged over all ten outer-loop folds, and the standard error is indicated by the surrounding gray region. As overall performance measures, AUBPRC and R90BP (the black dotted line) and their standard errors are shown. Statistical significance of performance relative to VARITY_R used a one-sided paired t test with 9 degrees of freedom (p values in brackets were indicated with a "*" where $p < 0.05$). Other test statistics, such as 95% confidence interval and effect size, are in Table S10.

it would be interesting in future studies to expand the gene/variant set based on more permissive identification of relevant genes/variants. VARITY models might also be used for identification of candidate causal variants in other genes, e.g., in patients for whom a predicted-damaging variant has not been observed in an already-known disease gene.

More broadly, we used nested cross-validation to estimate the generalization performance of VARITY models on the core set of ClinVar[44] variants. We note that the set of ClinVar[44] variants is influenced by historical ascertainment biases, in that they are enriched in disease genes discovered earlier, and some annotations were most likely influenced by early computational methods, such as BLOSUM[15] and SIFT.[25,26] Therefore, although ClinVar[44] variants enable useful measures of relative performance, it is difficult to estimate performance on an absolute scale for variants that will be observed in the future. Although the *de novo* variant evaluation set was free from these biases, it was necessarily limited in scope so that VARITY's generalization performance should in the future also be evaluated with additional independent test sets.

We provided information about which features and feature combinations were most useful in training VARITY models and feature contributions for the prediction made for each variant. We also note that the quantitative VARITY scores themselves can provide some intuition, e.g., there is a clear trend relating VARITY_R scores to the probability of pathogenicity (Figure S16).

The gradient boosted tree method has the advantage that training and testing examples for which some feature values are missing can be used directly without requiring imputation. Nevertheless, predictions may be less accurate for variants that are missing important features such as conservation-based scores and structure associated features. For example, if we remove all structural features from the core set, VARITY_R nested cross-validation performance (via AUBPRC) dropped from 92% to 91.5%.

For VARITY training, we eliminated protein-identifying features to reduce protein-specific biases in our training set. However, we might want to augment VARITY scores in the future by explicitly deriving a prior probability of missense variant pathogenicity (i.e., the probability of pathogenicity before considering the identity of the

missense variant) for each protein or protein region. However, because these priors may vary with the application setting, we suggest that the user should be explicitly informed about these priors and given the option to adjust them.

There are several avenues for future improvement of VARITY models. More training examples could be added, e.g., adding putatively benign variants from primates as PrimateAI[21] has done. One might also develop specialized models for different classes of proteins or variants, e.g., those in predicted membrane-spanning domains. More quality-informative properties could be added to improve add-on set weighting. More features could also be added, e.g., based on knowledge of genetic or protein-protein interactions. We are also currently lacking features related to the typical mode of inheritance for a given gene and associated disease (e.g., dominant or recessive), the typical mechanistic class (e.g., gain or loss of function), or typical penetrance or variable expressivity of variants. Although adding mode-of-inheritance features would be complicated by the fact that this information is often unavailable and can differ for different variants within the same gene, it could improve performance in general.

To our knowledge, no previous computational variant effect prediction method has used the strategy of weighting variants used in training. However, it is not uncommon in machine learning to weight training examples. For example, the AdaBoost ensemble algorithm assigns higher weight to examples misclassified by previous classifiers in each learning iteration.[62] Where multiple annotators have provided possibly noisy labels, the weight and the true label of each example can be learned together with the model via a maximum-likelihood approach.[63] We also note that the modeling approach we describe is just one way to address the problem of model learning with noisy labels, which is the subject of intensive research especially within the deep learning field.[64–68] Given that variant effect interpretation is greatly limited by the availability of high-confidence unbiased training data, this field will be worth watching closely for future inspiration.

Other models tuned for other types of variation could employ this framework by simply changing the core and add-on training examples. For example, a computational model specialized for predicting the pathogenicity of variation in membrane-spanning domains could be trained by limiting the core set to variants from regions predicted to be in the membrane while still using all other variants as add-on training sets subject to differential weighting. Models for predicting deleteriousness of nucleotide-level variation in non-coding regions or spliced regions would require more extensive separate assembly of relevant features and both core and add-on variant sets but could then benefit from the algorithmic framework we describe here.

As the performance of computational variant effect predictors improves, an important unsolved problem is how to translate these gains to have a commensurate impact on clinical variant interpretation. The increasing quantity of systematically collected large-scale functional evidence about missense variation has led to new recommendations for its use in the context of clinical interpretation.[69] We expect that a similarly quantitative evaluation of computational predictions will support a more nuanced use of computational evidence in clinical variant interpretation.

Users may search, browse, and download both VARITY_R and VARITY_ER scores (with associated feature contributions) for any of ~18,000 human proteins via the VARITY web portal (see web resources).

Data and code availability

All weighted core and add-on training data for the VARITY_R model can be found at http://varity.varianteffect.org/downloads/VARITY_R_training.csv and, for the VARITY_ER model, at http://varity.varianteffect.org/downloads/VARITY_ER_training.csv. Additional validation data (for *de novo* missense variants in neurodevelopmental disorder case/control studies and variants for six human-disease-related variant-effect maps) can be found at http://varity.varianteffect.org/downloads/VARITY_validation.zip. All sources for features used in training are summarized in Tables S1 and S2, and sources for labeling training variants can be found in Table S3. All data are publicly available from the sources listed, except for (1) three MAVE datasets for *NCS1* (MIM: 603315; MaveDB: [urn:mavedb:00000065-a-1]), *GDI1* (MIM: 300104; MaveDB: [urn:mavedb:00000066-a-1]), and *TECR* (MIM: 610057; MaveDB: [urn:mavedb:00000067-a-1]), which are currently unpublished but have been deposited to MaveDB[14] (and will be made available no later than 6 months after publication of this study), and (2) HGMD,[46] which was obtained under a commercial license that is generally available from QIAGEN/BioBase. All python code for training VARITY models is available at https://github.com/joewuca/varity.

Supplemental information

Supplemental information can be found online at https://doi.org/10.1016/j.ajhg.2021.08.012.

Acknowledgments

We thank Thomas Hu for computing cluster support. This work was supported by a Canadian Institutes of Health Research Foundation grant (F.P.R.), by the National Human Genome Research Institute of the National Institutes of Health Center of Excellence in Genomic Science Initiative (NIH grants P50HG004233 and RM1HG010461), by the Canada Excellence Research Chairs Program (F.P.R.), and by the One Brave Idea Initiative (jointly funded by the American Heart Association, Verily Life Sciences, and Astra-Zeneca).

Declaration of interests

F.P.R. is a scientific advisor holding shares in Constantiam Biosciences and BioSymetrics and a Ranomics shareholder. S.S. is currently employed by Sanofi Pasteur (Canada). The authors declare no other competing interests.

Received: May 24, 2021
Accepted: August 18, 2021
Published: September 21, 2021

Web resources

ClinVar, https://www.ncbi.nlm.nih.gov/clinvar/
dbNSFP, https://sites.google.com/site/jpopgen/dbNSFP
gnomAD, https://gnomad.broadinstitute.org/
HGMD, http://www.hgmd.cf.ac.uk/
HumsaVar, https://www.uniprot.org/docs/humsavar
MaveDB, https://www.mavedb.org/
VARITY, http://varity.varianteffect.org/

References

1. Manolio, T.A., Collins, F.S., Cox, N.J., Goldstein, D.B., Hindorff, L.A., Hunter, D.J., McCarthy, M.I., Ramos, E.M., Cardon, L.R., Chakravarti, A., et al. (2009). Finding the missing heritability of complex diseases. Nature *461*, 747–753.
2. Tam, V., Patel, N., Turcotte, M., Bossé, Y., Paré, G., and Meyre, D. (2019). Benefits and limitations of genome-wide association studies. Nat. Rev. Genet. *20*, 467–484.
3. Weile, J., and Roth, F.P. (2018). Multiplexed assays of variant effects contribute to a growing genotype-phenotype atlas. Hum. Genet. *137*, 665–678.
4. Starita, L.M., Ahituv, N., Dunham, M.J., Kitzman, J.O., Roth, F.P., Seelig, G., Shendure, J., and Fowler, D.M. (2017). Variant Interpretation: Functional Assays to the Rescue. Am. J. Hum. Genet. *101*, 315–325.
5. Fowler, D.M., and Fields, S. (2014). Deep mutational scanning: a new style of protein science. Nat. Methods *11*, 801–807.
6. Weile, J., Sun, S., Cote, A.G., Knapp, J., Verby, M., Mellor, J.C., Wu, Y., Pons, C., Wong, C., van Lieshout, N., et al. (2017). A framework for exhaustively mapping functional missense variants. Mol. Syst. Biol. *13*, 957.
7. Starita, L.M., Young, D.L., Islam, M., Kitzman, J.O., Gullingsrud, J., Hause, R.J., Fowler, D.M., Parvin, J.D., Shendure, J., and Fields, S. (2015). Massively parallel functional analysis of BRCA1 RING domain variants. Genetics *200*, 413–422.
8. Findlay, G.M., Daza, R.M., Martin, B., Zhang, M.D., Leith, A.P., Gasperini, M., Janizek, J.D., Huang, X., Starita, L.M., and Shendure, J. (2018). Accurate classification of BRCA1 variants with saturation genome editing. Nature *562*, 217–222.
9. Matreyek, K.A., Starita, L.M., Stephany, J.J., Martin, B., Chiasson, M.A., Gray, V.E., Kircher, M., Khechaduri, A., Dines, J.N., Hause, R.J., et al. (2018). Multiplex assessment of protein variant abundance by massively parallel sequencing. Nat. Genet. *50*, 874–882.
10. Sun, S., Weile, J., Verby, M., Wu, Y., Wang, Y., Cote, A.G., Fotiadou, I., Kitaygorodsky, J., Vidal, M., Rine, J., et al. (2020). A proactive genotype-to-patient-phenotype map for cystathionine beta-synthase. Genome Med. *12*, 13.
11. Chiasson, M.A., Rollins, N.J., Stephany, J.J., Sitko, K.A., Matreyek, K.A., Verby, M., Sun, S., Roth, F.P., DeSloover, D., Marks, D.S., et al. (2020). Multiplexed measurement of variant abundance and activity reveals VKOR topology, active site and human variant impact. eLife *9*, 1–25.
12. Sun, S., Yang, F., Tan, G., Costanzo, M., Oughtred, R., Hirschman, J., Theesfeld, C.L., Bansal, P., Sahni, N., Yi, S., et al. (2016). An extended set of yeast-based functional assays accurately identifies human disease mutations. Genome Res. *26*, 670–680.
13. Weile, J., Kishore, N., Sun, S., Maaieh, R., Verby, M., Li, R., Fotiadou, I., Kitaygorodsky, J., Wu, Y., Holenstein, A., et al. (2021). Shifting landscapes of human MTHFR missense-variant effects. Am. J. Hum. Genet. *108*, 1283–1300.
14. Esposito, D., Weile, J., Shendure, J., Starita, L.M., Papenfuss, A.T., Roth, F.P., Fowler, D.M., and Rubin, A.F. (2019). MaveDB: an open-source platform to distribute and interpret data from multiplexed assays of variant effect. Genome Biol. *20*, 223.
15. Henikoff, S., and Henikoff, J.G. (1992). Amino acid substitution matrices from protein blocks. Proc. Natl. Acad. Sci. USA *89*, 10915–10919.
16. Ioannidis, N.M., Rothstein, J.H., Pejaver, V., Middha, S., McDonnell, S.K., Baheti, S., Musolf, A., Li, Q., Holzinger, E., Karyadi, D., et al. (2016). REVEL: An Ensemble Method for Predicting the Pathogenicity of Rare Missense Variants. Am. J. Hum. Genet. *99*, 877–885.
17. Jagadeesh, K.A., Wenger, A.M., Berger, M.J., Guturu, H., Stenson, P.D., Cooper, D.N., Bernstein, J.A., and Bejerano, G. (2016). M-CAP eliminates a majority of variants of uncertain significance in clinical exomes at high sensitivity. Nat. Genet. *48*, 1581–1586.
18. Kircher, M., Witten, D.M., Jain, P., O'Roak, B.J., Cooper, G.M., and Shendure, J. (2014). A general framework for estimating the relative pathogenicity of human genetic variants. Nat. Genet. *46*, 310–315.
19. Adzhubei, I.A., Schmidt, S., Peshkin, L., Ramensky, V.E., Gerasimova, A., Bork, P., Kondrashov, A.S., and Sunyaev, S.R. (2010). A method and server for predicting damaging missense mutations. Nat. Methods *7*, 248–249.
20. Adzhubei, I., Jordan, D.M., and Sunyaev, S.R. (2013). Predicting functional effect of human missense mutations using PolyPhen-2, Chapter 7 (Curr. Protoc. Hum. Genet).
21. Sundaram, L., Gao, H., Padigepati, S.R., McRae, J.F., Li, Y., Kosmicki, J.A., Fritzilas, N., Hakenberg, J., Dutta, A., Shon, J., et al. (2018). Predicting the clinical impact of human mutation with deep neural networks. Nat. Genet. *50*, 1161–1170.
22. Gray, V.E., Hause, R.J., Luebeck, J., Shendure, J., and Fowler, D.M. (2018). Quantitative Missense Variant Effect Prediction Using Large-Scale Mutagenesis Data. Cell Syst. *6*, 116–124.e3.
23. Ionita-Laza, I., McCallum, K., Xu, B., and Buxbaum, J.D. (2016). A spectral approach integrating functional genomic annotations for coding and noncoding variants. Nat. Genet. *48*, 214–220.
24. Choi, Y., Sims, G.E., Murphy, S., Miller, J.R., and Chan, A.P. (2012). Predicting the functional effect of amino acid substitutions and indels. PLoS ONE *7*, e46688.
25. Kumar, P., Henikoff, S., and Ng, P.C. (2009). Predicting the effects of coding non-synonymous variants on protein function using the SIFT algorithm. Nat. Protoc. *4*, 1073–1081.
26. Vaser, R., Adusumalli, S., Leng, S.N., Sikic, M., and Ng, P.C. (2016). SIFT missense predictions for genomes. Nat. Protoc. *11*, 1–9.
27. Hopf, T.A., Ingraham, J.B., Poelwijk, F.J., Schärfe, C.P.I., Springer, M., Sander, C., and Marks, D.S. (2017). Mutation effects predicted from sequence co-variation. Nat. Biotechnol. *35*, 128–135.
28. Gulko, B., Hubisz, M.J., Gronau, I., and Siepel, A. (2015). A method for calculating probabilities of fitness consequences for point mutations across the human genome. Nat. Genet. *47*, 276–283.
29. Chun, S., and Fay, J.C. (2009). Identification of deleterious mutations within three human genomes. Genome Res. *19*, 1553–1561.

30. Davydov, E.V., Goode, D.L., Sirota, M., Cooper, G.M., Sidow, A., and Batzoglou, S. (2010). Identifying a high fraction of the human genome to be under selective constraint using GERP++. PLoS Comput. Biol. *6*, e1001025.
31. Pollard, K.S., Hubisz, M.J., Rosenbloom, K.R., and Siepel, A. (2010). Detection of nonneutral substitution rates on mammalian phylogenies. Genome Res. *20*, 110–121.
32. Siepel, A., Bejerano, G., Pedersen, J.S., Hinrichs, A.S., Hou, M., Rosenbloom, K., Clawson, H., Spieth, J., Hillier, L.W., Richards, S., et al. (2005). Evolutionarily conserved elements in vertebrate, insect, worm, and yeast genomes. Genome Res. *15*, 1034–1050.
33. Garber, M., Guttman, M., Clamp, M., Zody, M.C., Friedman, N., and Xie, X. (2009). Identifying novel constrained elements by exploiting biased substitution patterns. Bioinformatics *25*, i54–i62.
34. Reva, B., Antipin, Y., and Sander, C. (2011). Predicting the functional impact of protein mutations: application to cancer genomics. Nucleic Acids Res. *39*, e118.
35. Dong, C., Wei, P., Jian, X., Gibbs, R., Boerwinkle, E., Wang, K., and Liu, X. (2015). Comparison and integration of deleteriousness prediction methods for nonsynonymous SNVs in whole exome sequencing studies. Hum. Mol. Genet. *24*, 2125–2137.
36. Quang, D., Chen, Y., and Xie, X. (2015). DANN: a deep learning approach for annotating the pathogenicity of genetic variants. Bioinformatics *31*, 761–763.
37. Schwarz, J.M., Cooper, D.N., Schuelke, M., and Seelow, D. (2014). MutationTaster2: mutation prediction for the deep-sequencing age. Nat. Methods *11*, 361–362.
38. Lu, Q., Hu, Y., Sun, J., Cheng, Y., Cheung, K.H., and Zhao, H. (2015). A statistical framework to predict functional non-coding regions in the human genome through integrated analysis of annotation data. Sci. Rep. *5*, 10576.
39. Shihab, H.A., Gough, J., Cooper, D.N., Day, I.N.M., and Gaunt, T.R. (2013). Predicting the functional consequences of cancer-associated amino acid substitutions. Bioinformatics *29*, 1504–1510.
40. Wong, K.C., and Zhang, Z. (2014). SNPdryad: predicting deleterious non-synonymous human SNPs using only orthologous protein sequences. Bioinformatics *30*, 1112–1119.
41. Samocha, K., Kosmicki, J., Karczewski, K., O'Donnell-Luria, A., Pierce-Hoffman, E., MacArthur, D., Neale, B., and Daly, M. (2017). Regional missense constraint improves variant deleteriousness prediction. bioRxiv *10.1101/148353*.
42. Riesselman, A.J., Ingraham, J.B., and Marks, D.S. (2018). Deep generative models of genetic variation capture the effects of mutations. Nat. Methods *15*, 816–822.
43. Richards, S., Aziz, N., Bale, S., Bick, D., Das, S., Gastier-Foster, J., Grody, W.W., Hegde, M., Lyon, E., Spector, E., et al.; ACMG Laboratory Quality Assurance Committee (2015). Standards and guidelines for the interpretation of sequence variants: a joint consensus recommendation of the American College of Medical Genetics and Genomics and the Association for Molecular Pathology. Genet. Med. *17*, 405–424.
44. Landrum, M.J., Lee, J.M., Benson, M., Brown, G.R., Chao, C., Chitipiralla, S., Gu, B., Hart, J., Hoffman, D., Jang, W., et al. (2018). ClinVar: improving access to variant interpretations and supporting evidence. Nucleic Acids Res. *46* (D1), D1062–D1067.
45. The UniProt Consortium (2017). UniProt: the universal protein knowledgebase. Nucleic Acids Res. *45* (D1), D158–D169.
46. Stenson, P.D., Mort, M., Ball, E.V., Evans, K., Hayden, M., Heywood, S., Hussain, M., Phillips, A.D., and Cooper, D.N. (2017). The Human Gene Mutation Database: towards a comprehensive repository of inherited mutation data for medical research, genetic diagnosis and next-generation sequencing studies. Hum. Genet. *136*, 665–677.
47. Karczewski, K.J., Francioli, L.C., Tiao, G., Cummings, B.B., Alföldi, J., Wang, Q., Collins, R.L., Laricchia, K.M., Ganna, A., Birnbaum, D.P., et al.; Genome Aggregation Database Consortium (2020). The mutational constraint spectrum quantified from variation in 141,456 humans. Nature *581*, 434–443.
48. Liu, X., Wu, C., Li, C., and Boerwinkle, E. (2016). dbNSFP v3.0: A One-Stop Database of Functional Predictions and Annotations for Human Nonsynonymous and Splice-Site SNVs. Hum. Mutat. *37*, 235–241.
49. Krissinel, E., and Henrick, K. (2007). Inference of macromolecular assemblies from crystalline state. J. Mol. Biol. *372*, 774–797.
50. Buchan, D.W.A., and Jones, D.T. (2019). The PSIPRED Protein Analysis Workbench: 20 years on. Nucleic Acids Res. *47* (W1), W402–W407.
51. El-Gebali, S., Mistry, J., Bateman, A., Eddy, S.R., Luciani, A., Potter, S.C., Qureshi, M., Richardson, L.J., Salazar, G.A., Smart, A., et al. (2019). The Pfam protein families database in 2019. Nucleic Acids Res. *47* (D1), D427–D432.
52. Wu, Y., Weile, J., Cote, A.G., Sun, S., Knapp, J., Verby, M., and Roth, F.P. (2019). A web application and service for imputing and visualizing missense variant effect maps. Bioinformatics *35*, 3191–3193.
53. Chen, T., and Guestrin, C. (2016). XGBoost: A scalable tree boosting system. Proc. ACM SIGKDD Int. Conf. Knowl. Discov. Data Min. *13-17-Augu*, 785–794.
54. Livesey, B.J., and Marsh, J.A. (2020). Using deep mutational scanning to benchmark variant effect predictors and identify disease mutations. Mol. Syst. Biol. *16*, e9380.
55. Bergstra, J., Bardenet, R., Bengio, Y., and Kégl, B. (2011). Algorithms for Hyper-Parameter Optimization. Adv. Neural Inf. Process. Syst. *24*, 2546–2554.
56. Bergstra, J., Komer, B., Eliasmith, C., Yamins, D., and Cox, D.D. (2015). Hyperopt: A Python library for model selection and hyperparameter optimization. Comput. Sci. Discov. *8*, 014008.
57. Turner, T.N., Yi, Q., Krumm, N., Huddleston, J., Hoekzema, K., F Stessman, H.A., Doebley, A.L., Bernier, R.A., Nickerson, D.A., and Eichler, E.E. (2017). denovo-db: a compendium of human de novo variants. Nucleic Acids Res. *45* (D1), D804–D811.
58. Coe, B.P., Stessman, H.A.F., Sulovari, A., Geisheker, M.R., Bakken, T.E., Lake, A.M., Dougherty, J.D., Lein, E.S., Hormozdiari, F., Bernier, R.A., and Eichler, E.E. (2019). Neurodevelopmental disease genes implicated by de novo mutation and copy number variation morbidity. Nat. Genet. *51*, 106–116.
59. Lundberg, S.M., and Lee, S.I. (2017). A unified approach to interpreting model predictions. Adv. Neural Inf. Process. Syst. *2017-Decem*, 4766–4775.
60. Lundberg, S.M., Erion, G., Chen, H., DeGrave, A., Prutkin, J.M., Nair, B., Katz, R., Himmelfarb, J., Bansal, N., and Lee, S.-I. (2020). From local explanations to global understanding with explainable AI for trees. Nat. Mach. Intell. *2*, 56–67.
61. Grimm, D.G., Azencott, C.A., Aicheler, F., Gieraths, U., MacArthur, D.G., Samocha, K.E., Cooper, D.N., Stenson, P.D., Daly, M.J., Smoller, J.W., et al. (2015). The evaluation of tools used to predict the impact of missense variants is hindered by two types of circularity. Hum. Mutat. *36*, 513–523.

62. Freund, Y., and Schapire, R.E. (1997). A Decision-Theoretic Generalization of On-Line Learning and an Application to Boosting. J. Comput. Syst. Sci. *55*, 119–139.
63. Raykar, V.C., Yu, S., Zhao, L.H., Valadez, G.H., Florin, C., Bogoni, L., and Moy, L. (2010). Learning from crowds. J. Mach. Learn. Res. *11*, 1297–1322.
64. Vahdat, A. (2017). Toward Robustness against Label Noise in Training Deep Discriminative Neural Networks. Adv. Neural Inf. Process. Syst *2017-Decem*, 5597–5606.
65. Patrini, G., Rozza, A., Menon, A.K., Nock, R., and Qu, L. (2017). Making deep neural networks robust to label noise: A loss correction approach. Proc. - 30th IEEE Conf. Comput. Vis. Pattern Recognition CVPR 2017 *2017-Janua*, 2233–2241.
66. Tanaka, D., Ikami, D., Yamasaki, T., and Aizawa, K. (2018). Joint Optimization Framework for Learning with Noisy Labels. Proc. IEEE Comput. Soc. Conf. Comput. Vis. Pattern Recognit. *CVPR 2018*, 5552–5560.
67. Litany, O., and Freedman, D. (2018). SOSELETO: A Unified Approach to Transfer Learning and Training with Noisy Labels. arXiv *1805.09622v2*. https://arxiv.org/abs/1803.11364.
68. Yao, J., Wang, J., Tsang, I.W., Zhang, Y., Sun, J., Zhang, C., and Zhang, R. (2018). Deep Learning from Noisy Image Labels with Quality Embedding. IEEE Trans. Image Process. *28*, 1909–1922.
69. Brnich, S.E., Abou Tayoun, A.N., Couch, F.J., Cutting, G.R., Greenblatt, M.S., Heinen, C.D., Kanavy, D.M., Luo, X., McNulty, S.M., Starita, L.M., et al.; Clinical Genome Resource Sequence Variant Interpretation Working Group (2019). Recommendations for application of the functional evidence PS3/BS3 criterion using the ACMG/AMP sequence variant interpretation framework. Genome Med. *12*, 3.

The American Journal of Human Genetics, Volume *108*

Supplemental information

Improved pathogenicity prediction for rare human missense variants

Yingzhou Wu, Roujia Li, Song Sun, Jochen Weile, and Frederick P. Roth

Supplemental Figures

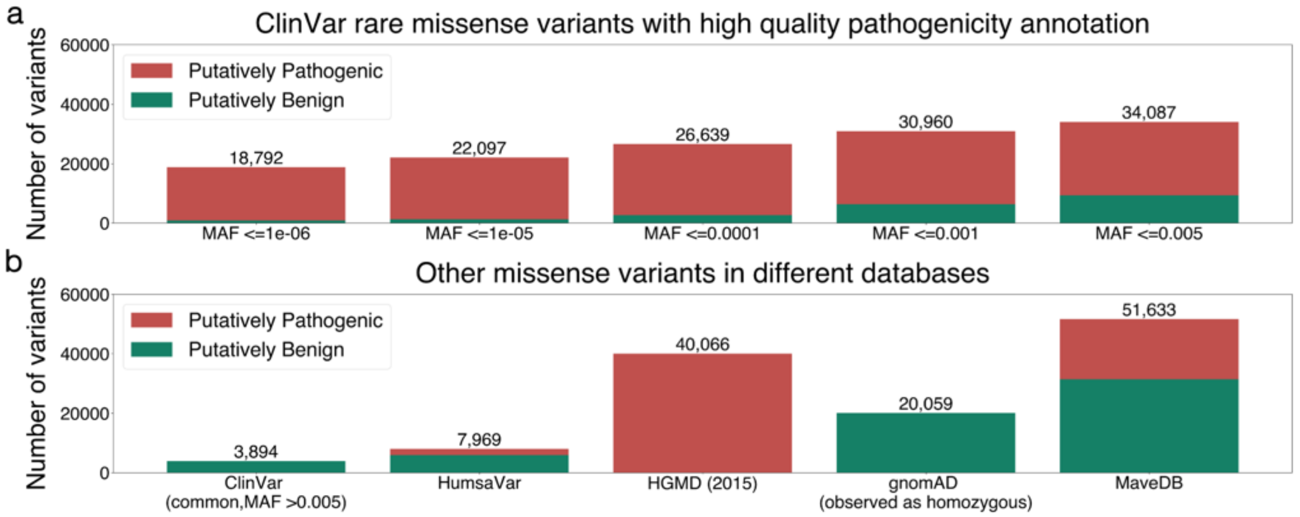

Figure S1: (a) The number of annotated variants from the high-quality ClinVAR[1] database with minor allele frequency (MAF) below the specified thresholds. (b) Other sources of variant training examples with less certain accuracy and representativity.

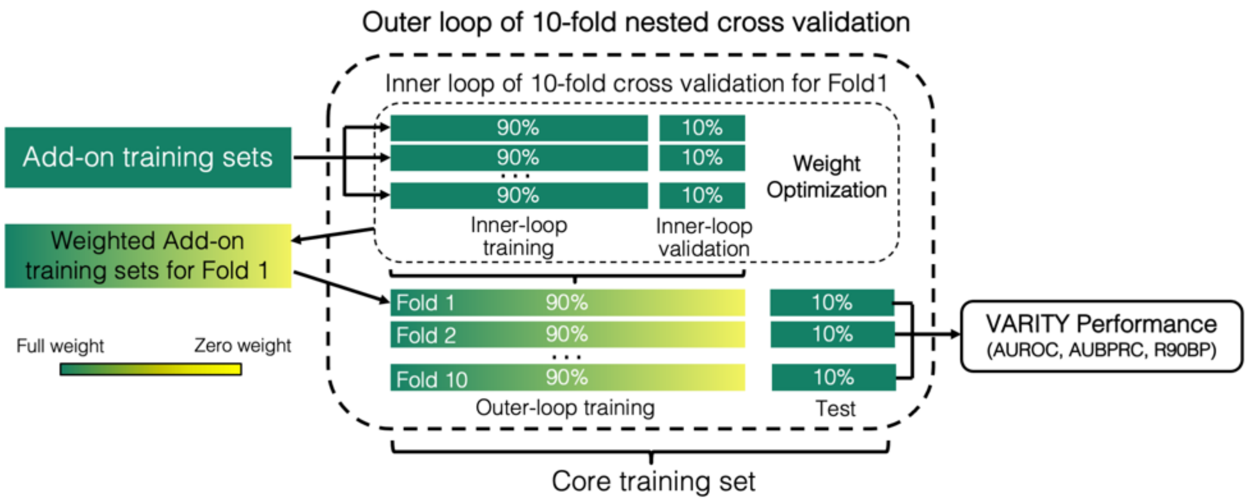

Figure S2: 10-fold nested cross validation. To measure VARITY performance, we used 10-fold nested cross validation, with the first fold of the outer loop ("Fold1") shown here for illustration. In each outer-loop, training sets (as weighted according to hyperparameters learned in the inner loop) were used to train VARITY models and test them using held-out test data. Three metrics—AUROC, AUBPRC and R90BP—were used for performance evaluation (see Methods).

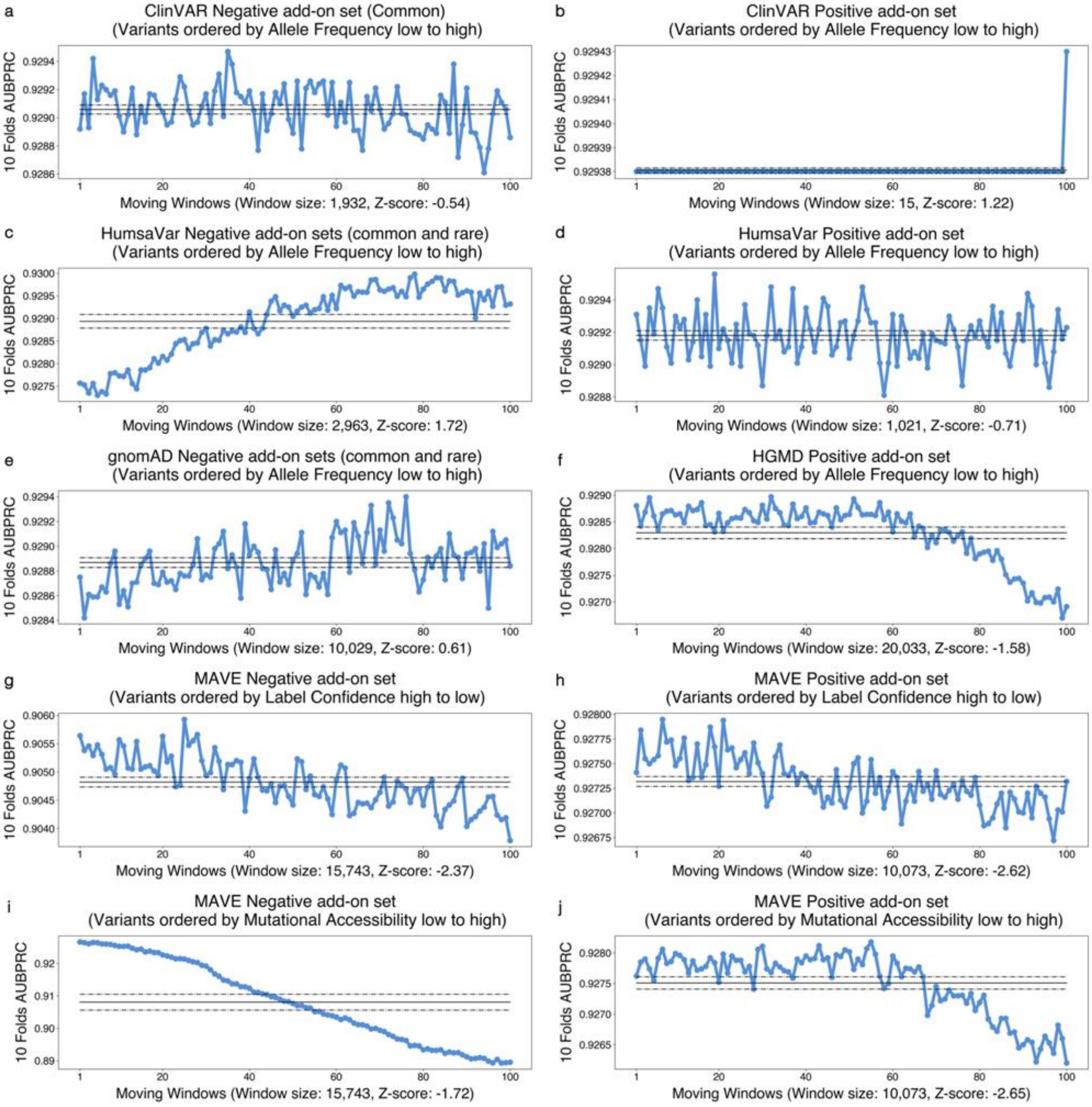

Figure S3: Moving window analysis for VARITY_R. Each panel illustrates assessment of a candidate informative property as a basis for weighting a single add-on set or combination of add-on sets. For Negative variants from HumsaVar[2] and gnomAD[3] (**Panel c and e**), the common (MAF>0.5%) and rare (MAF < 0.5%) add-on sets were combined for moving window analysis. Variants in add-on set(s) were ordered by the candidate informative property and 100 moving windows, each capturing same number of examples, were examined. To evaluate the predictive utility of each window, the model performance on the core set was estimated using 10-fold cross validation where the training examples in each fold was supplemented by examples in that moving window. Solid and dashed black lines indicate the mean ± standard error of VARITY_R performance measures over all moving windows. A Z-score was calculated to estimate direction and significance of the observed trend (see Methods).

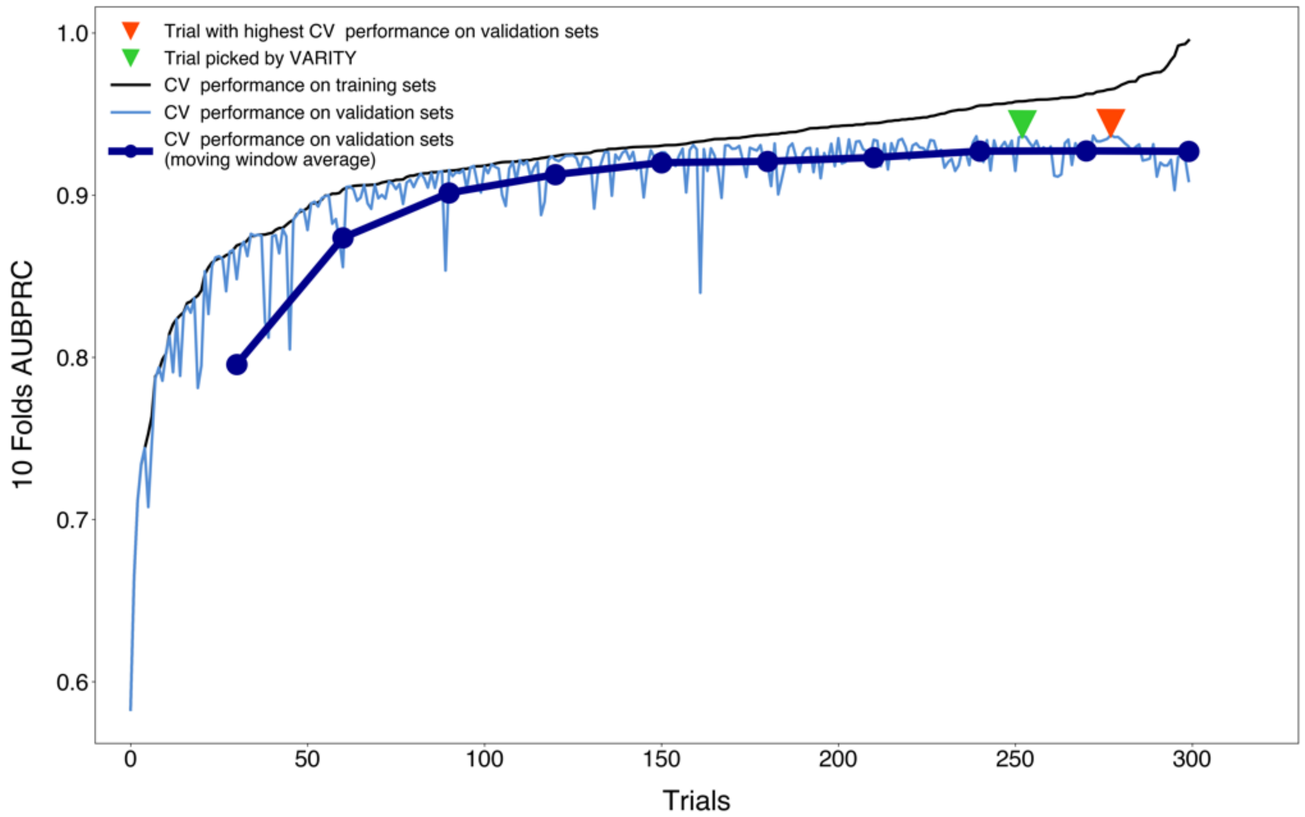

Figure S4: Hyperparameter tuning for the VARITY_R model. To optimize hyperparameters, 300 trials were performed using HyperOpt[4-5], each using a set of hyperparameter values suggested from previous trials (see Methods). To avoid overfitting in hyperparameter optimization, we did not simply choose hyperparameters showing the best numeric performance on validation sets (red triangle). Rather, the best hyperparameters (green triangle) were chosen as follows: 1) all trials were re-ordered from low to high AUBPRC (averaged over 10 training sets; black curve), and the average AUBPRC value from 10 validation sets was also shown (light blue curve); 2) a moving window (30 trials per window) was calculated for the cross-validation performance on validation sets (dark blue curve); 3) the 'fittest' moving window, i.e., the point at which the moving window performance starts to descend due to overfitting, was identified; and 4) Select the trial that performed best in cross-validation from within the fittest moving window (green triangle).

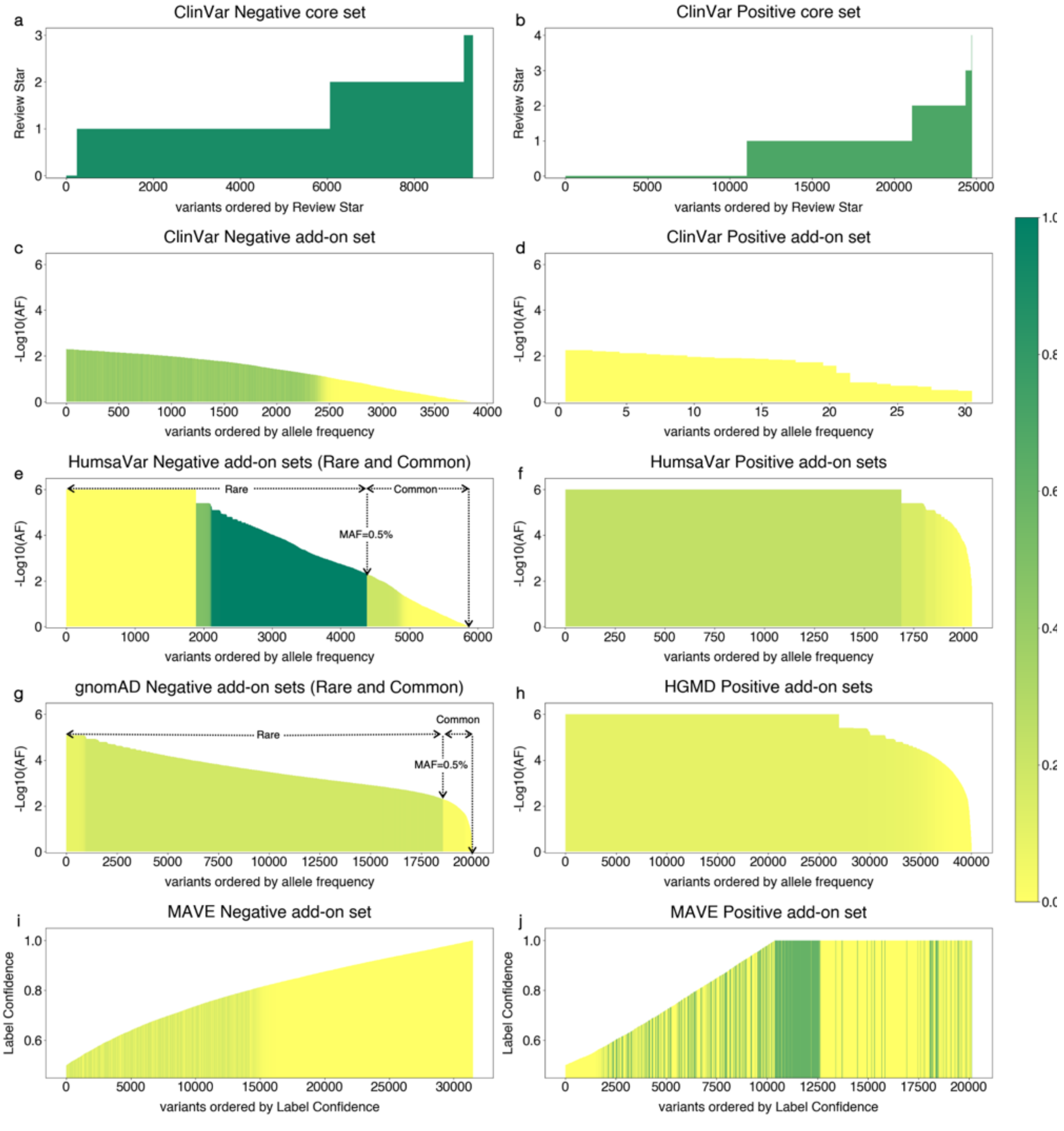

Figure S5: Weighted training sets for VARITY_R. Each plot illustrates the optimized weight with a color that varies between 0 (yellow; lowest weight) and 1 (green; highest weight) for a single core/add-on set or multiple core or add-on sets. For compactness, weighting for common and rare add-on sets of Negative training examples are shown together for HumsaVar[2] and gnomAD[3] (Panels **e** and **g**, respectively), while other Panels correspond to single core or add-on sets. For each plot, the x-axis indicates the rank of each variant ordered by the associated informative property (y-axis). Discontinuities in weight along the x-axis are possible where variant weights were based on multiple quality-informative properties (in which case the overall variant weight is the product of individual weights; see Methods and Table S3).

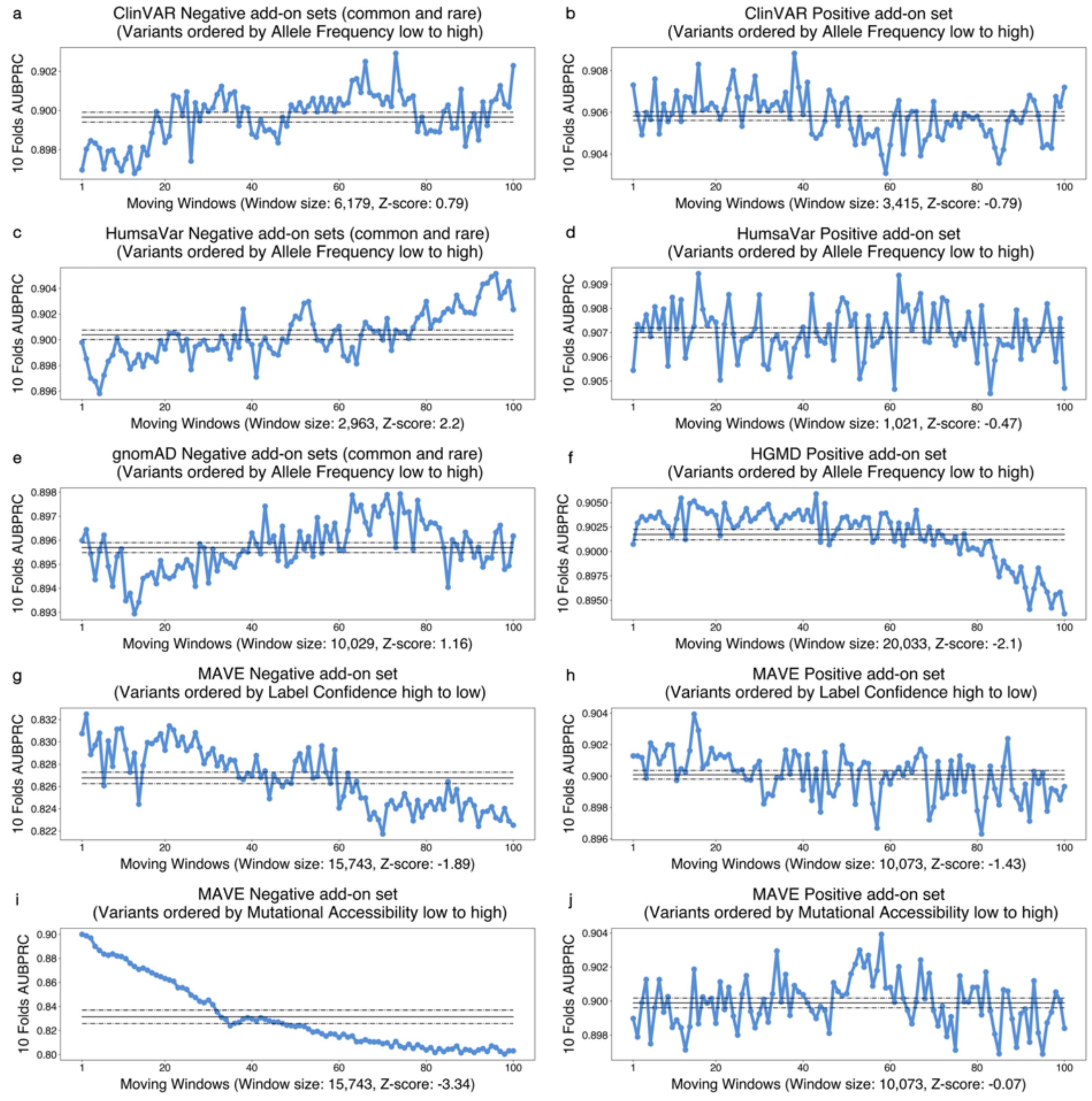

Figure S6: Moving window analysis for VARITY_ER. Each panel illustrates assessment of a candidate informative property as a basis for weighting a single add-on set or combination of add-on sets. For Negative variants from HumsaVar[2] and gnomAD[3] (**Panel c and e**), the common (MAF>=0.5%) and rare (MAF < 0.5%) add-on sets were combined for moving window analysis. Variants in add-on set(s) were ordered by the candidate informative property and 100 moving windows, each capturing same number of examples, were examined. To evaluate the predictive utility of each window, the model performance on the core set was estimated using 10-fold cross validation where the training examples in each fold was supplemented by examples in that moving window. Solid and dashed black lines indicate the mean ± standard error of VARITY_R performance measures over all moving windows. A Z-score was calculated to estimate direction and significance of the observed trend (see Methods).

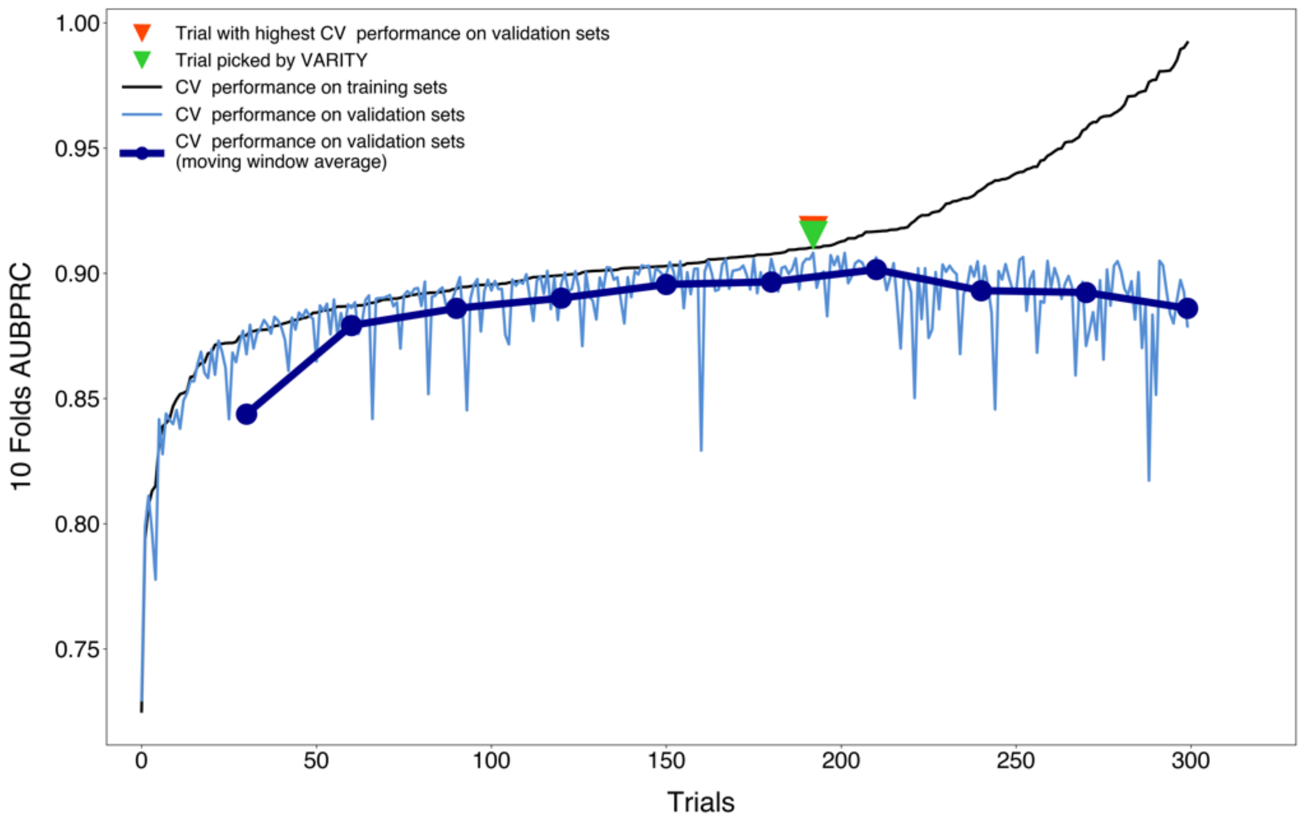

Figure S7: Hyperparameter tuning for the VARITY_ER model. To optimize hyperparameters, 300 trials were performed using HyperOpt[45], each using a set of hyperparameter values suggested from previous trials (see Methods). To avoid overfitting in hyperparameter optimization, we did not simply choose hyperparameters showing the best numeric performance on validation sets (red triangle). Rather, the best hyperparameters (green triangle) were chosen as follows: 1) all trials were re-ordered from low to high AUBPRC (averaged over 10 training sets; black curve), and the average AUBPRC value from 10 validation sets was also shown (light blue curve); 2) a moving window (30 trials per window) was calculated for the cross-validation performance on validation sets (dark blue curve); 3) the 'fittest' moving window, i.e., the point at which the moving window performance starts to descend due to overfitting, was identified; and 4) Select the trial that performed best in cross-validation from within the fittest moving window (green triangle).

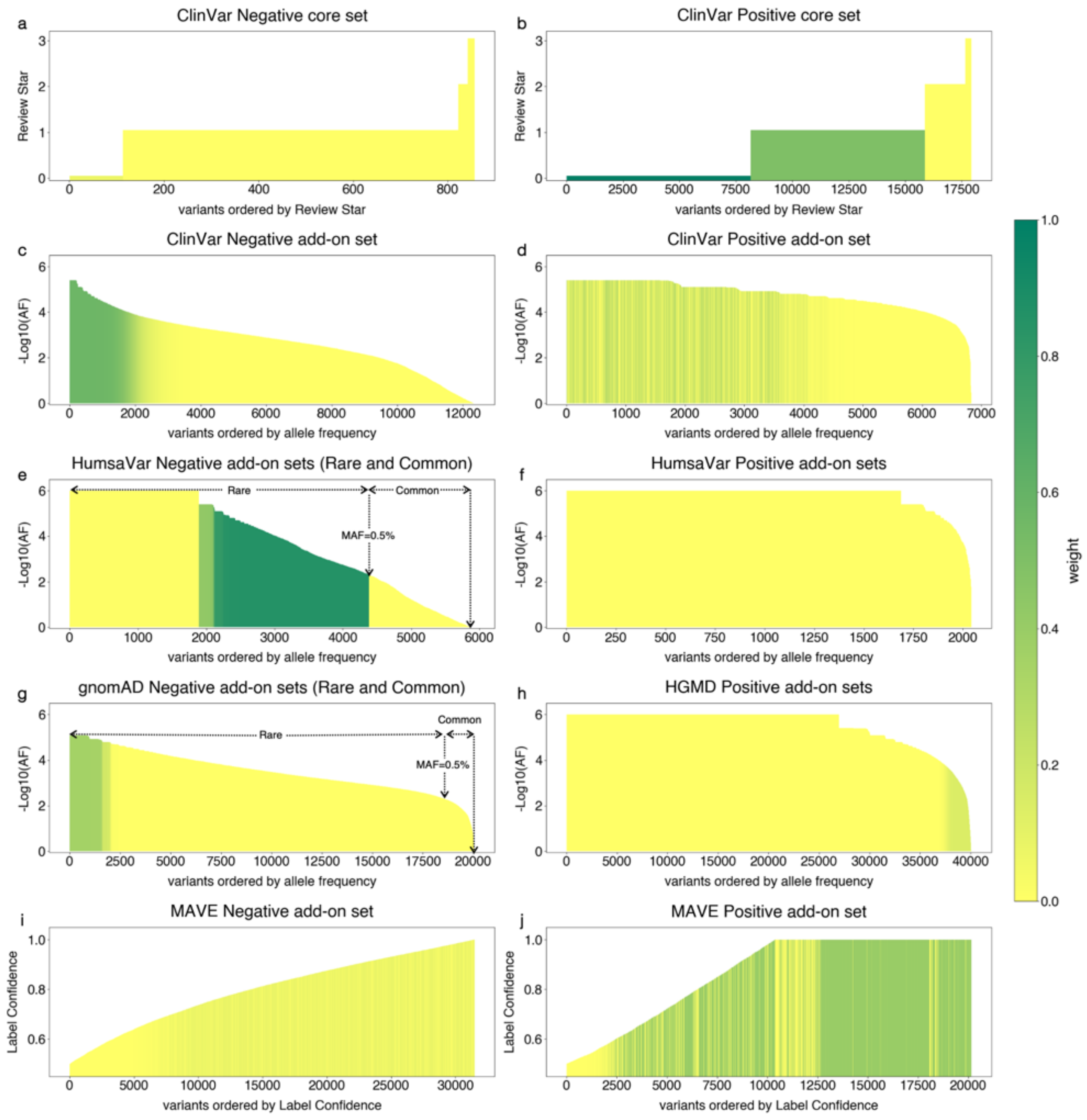

Figure S8: Weighted training sets for VARITY_ER. Each plot illustrates the optimized weight with a color that varies between 0 (yellow; lowest weight) and 1 (green; highest weight) for a single core/add-on set or multiple core or add-on sets. For compactness, weighting for common and rare add-on sets of Negative training examples are shown together for HumsaVar[2] and gnomAD[3] (Panels **e** and **g**, respectively), while other Panels correspond to single core or add-on sets. For each plot, the x-axis indicates the rank of each variant ordered by the associated informative property (y-axis). Discontinuities in weight along the x-axis are possible where variant weights were based on multiple quality-informative properties (in which case the overall variant weight is the product of individual weights; see Methods and Table S3).

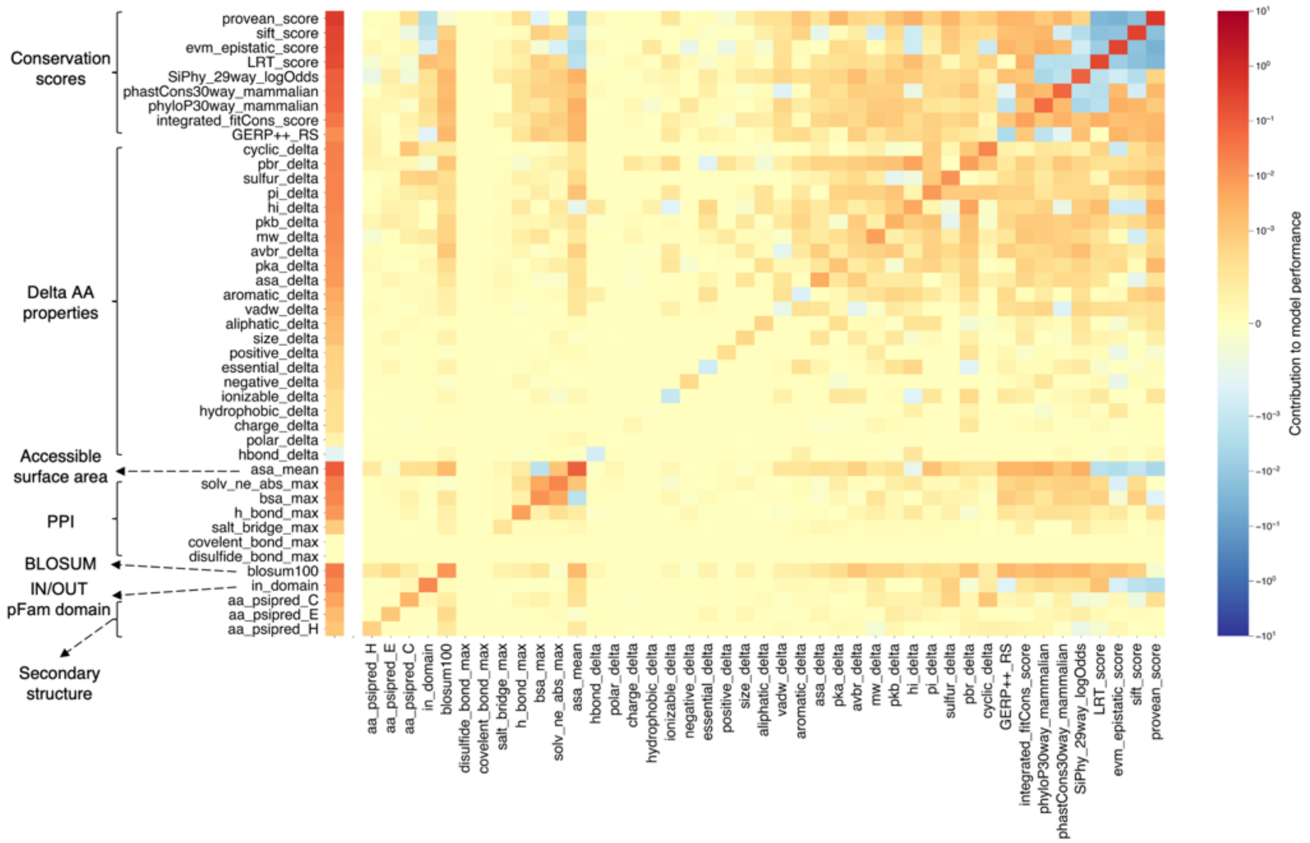

Figure S9: Individual feature contributions to VARITY_R model performance. The contribution of each feature to VARITY_R model performance was combined by weighted averaging across all training examples, using the weight of each training example that was optimized during hyperparameter tuning. The first column (left) indicates the total contribution to model performance of each feature, which consists of a feature-independent contribution (matrix cell on the diagonal on the corresponding row) and pair-wise differential feature contributions (non-diagonal matrix cells on the corresponding row). Red and blue color indicates positive and negative contribution to model performance respectively. A blue colored cell for pair-wise differential contribution indicates there is some redundancy between two features (e.g., between different conservation scores). The description of each feature can be found in Table S1.

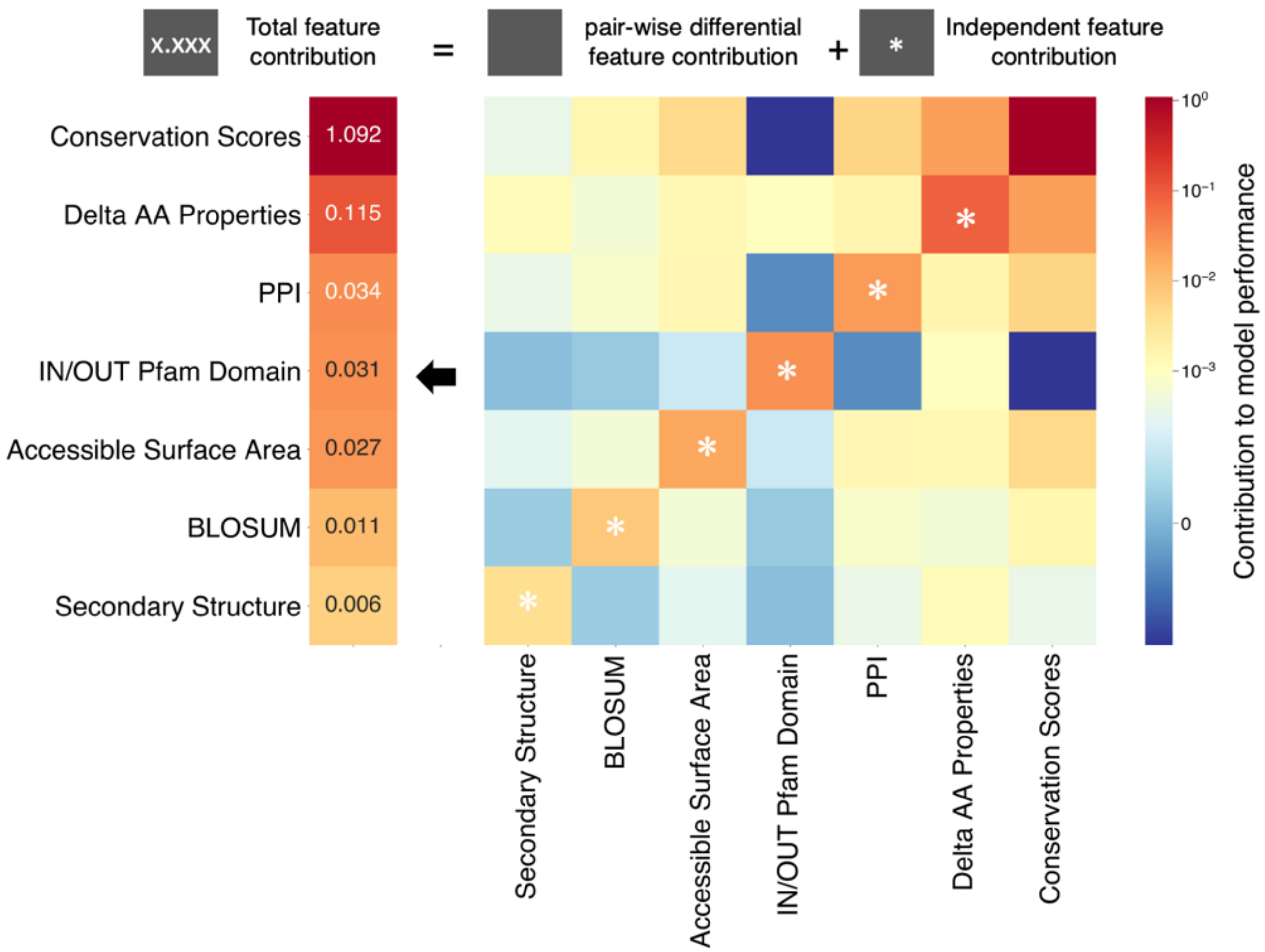

Figure S10: Feature group contributions to VARITY_ER model performance. The contribution of each feature group to VARITY_R model performance was averaged (weighted) across all training examples using the weight of each training example as optimized during hyperparameter tuning. The first column (left) indicates the total contribution to model performance of each feature group. For each feature group, the total contribution can be decomposed into the individual feature contribution (matrix cell with a star symbol on the corresponding row) and the differential contribution of that feature when it is combined with each other feature group (matrix cells without a star symbol on the corresponding row). Red and blue color indicates positive and negative contribution to model performance respectively. A blue colored cell for pair-wise differential feature contribution indicates there is a certain amount of redundancy between two feature groups (e.g., between Conservation Scores and IN/OUT Pfam domain).

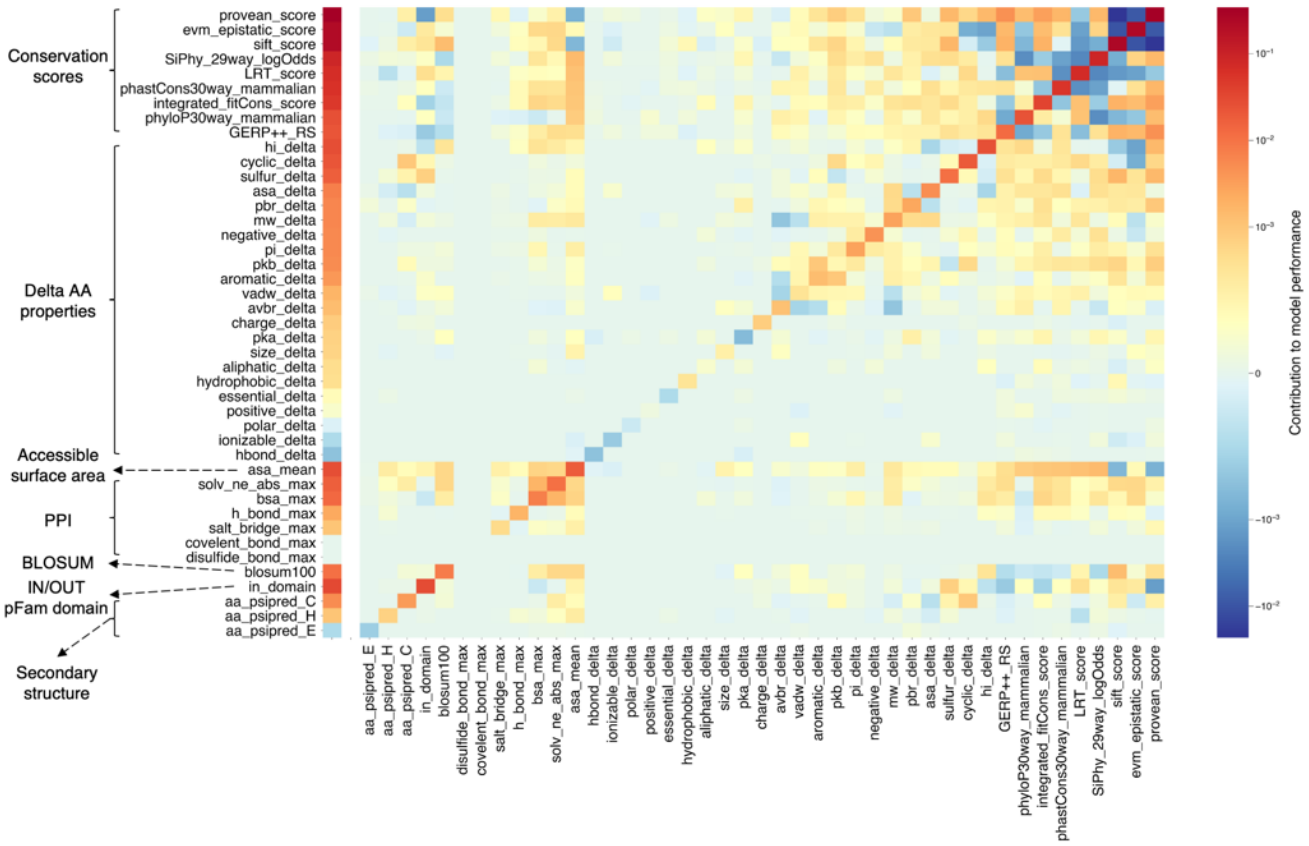

Figure S11: Individual feature contributions to VARITY_ER model performance. The contribution of each feature to VARITY_R model performance was combined by weighted averaging across all training examples, using the weight of each training example that was optimized during hyperparameter tuning. The first column (left) indicates the total contribution to model performance of each feature, which consists of a feature-independent contribution (matrix cell on the diagonal on the corresponding row) and pair-wise differential feature contributions (non-diagonal matrix cells on the corresponding row). Red and blue color indicates positive and negative contribution to model performance respectively. A blue colored cell for pair-wise differential contribution indicates there is some redundancy between two features (e.g., between different conservation scores). The description of each feature can be found in Table S1.

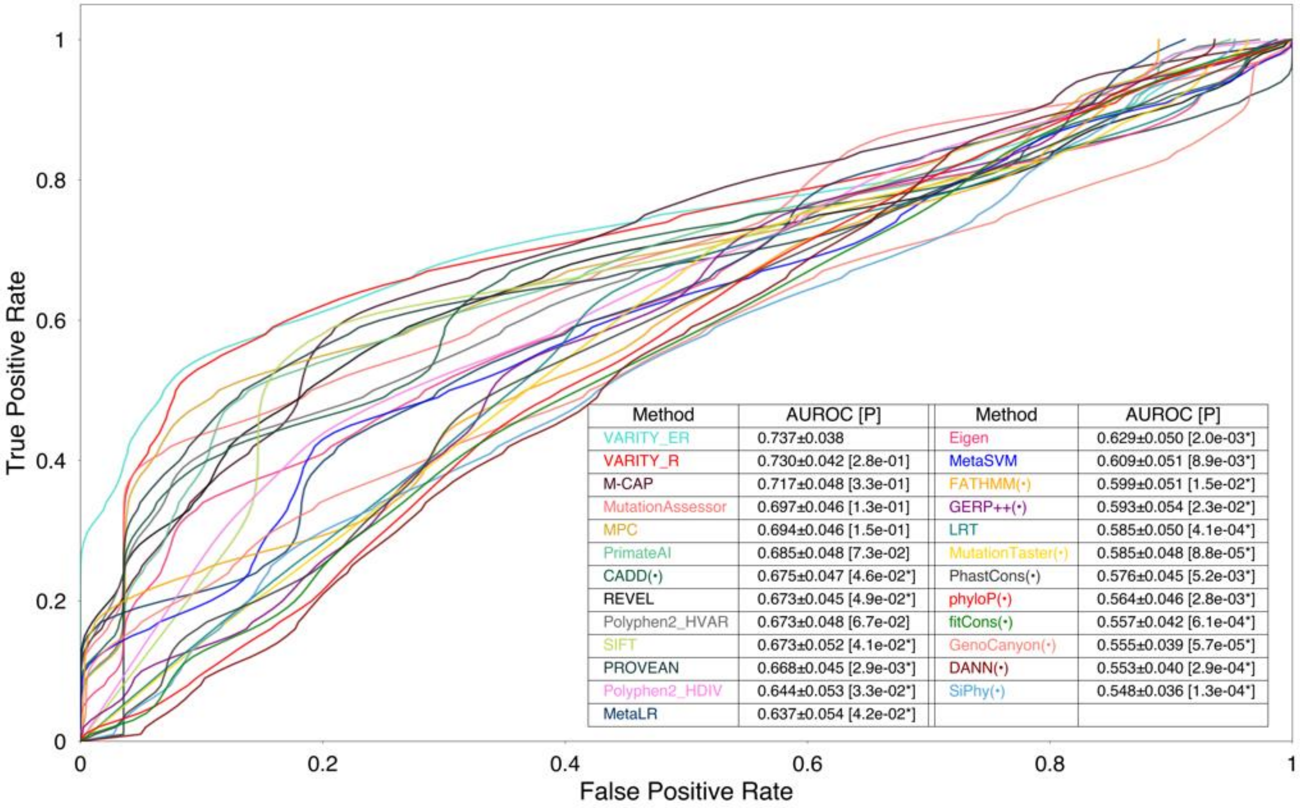

Figure S12: Assessing VARITY ROC performance for *de novo* variants observed in neurodevelopmental disorder studies. Here we show balanced ROC curves for VARITY_ER, VARITY_R and 23 other variant pathogenicity predictors, using a test set of 215 rare *de novo* variants (188 positive and 27 negative examples, see Methods). Recall was averaged over 2,000 bootstrapped test sets with standard error indicated by the surrounding grey region. As overall performance measures, AUROC and their standard errors are shown. Predictors designed specifically for nucleotide variants are indicated with a '(•)'. Statistical significance relative to VARITY_ER was assessed using a one-sided Z test applied to 2,000 bootstrapped test sets (P-values are shown in brackets, with '*' indicating where $P < 0.05$). Other test statistics such as 95% confidence interval and effect size can be found in Table S6.

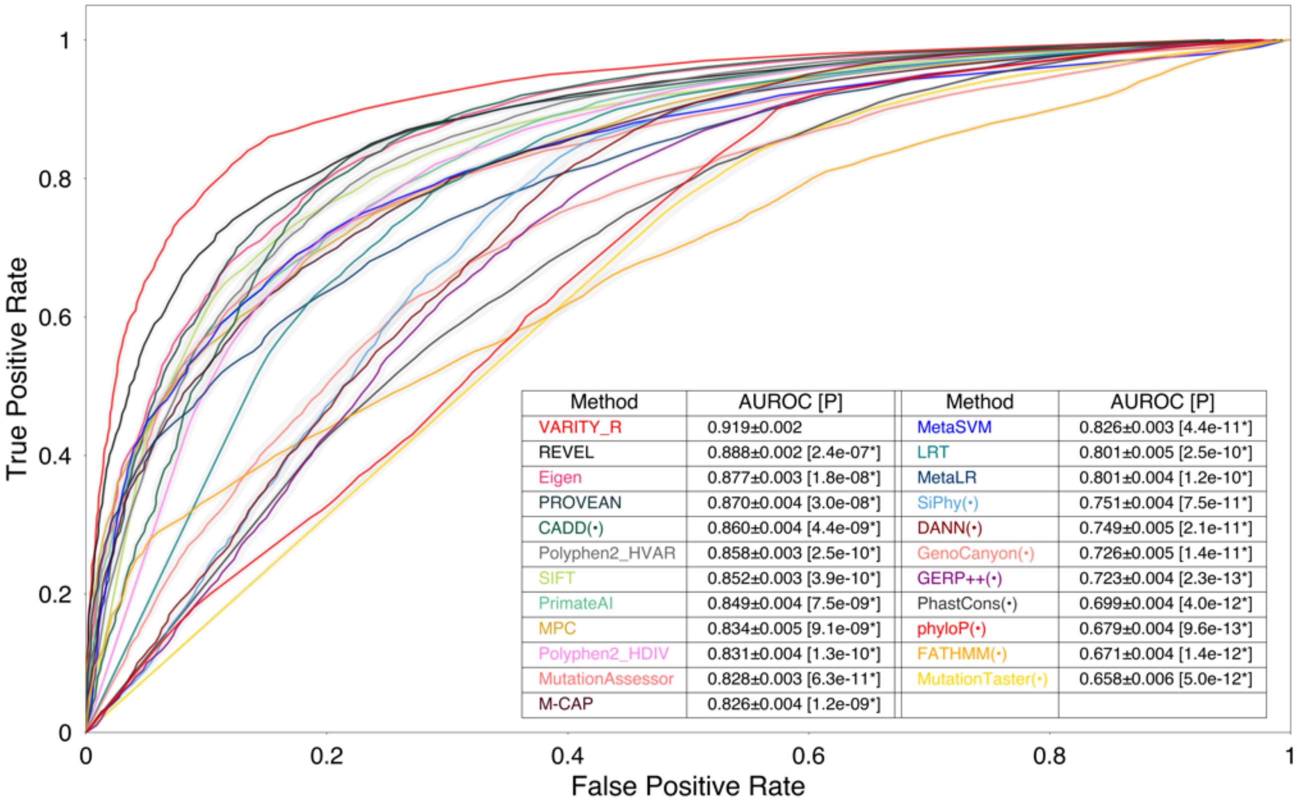

Method	AUROC [P]	Method	AUROC [P]
VARITY_R	0.919±0.002	MetaSVM	0.826±0.003 [4.4e-11*]
REVEL	0.888±0.002 [2.4e-07*]	LRT	0.801±0.005 [2.5e-10*]
Eigen	0.877±0.003 [1.8e-08*]	MetaLR	0.801±0.004 [1.2e-10*]
PROVEAN	0.870±0.004 [3.0e-08*]	SiPhy(•)	0.751±0.004 [7.5e-11*]
CADD(•)	0.860±0.004 [4.4e-09*]	DANN(•)	0.749±0.005 [2.1e-11*]
Polyphen2_HVAR	0.858±0.003 [2.5e-10*]	GenoCanyon(•)	0.726±0.005 [1.4e-11*]
SIFT	0.852±0.003 [3.9e-10*]	GERP++(•)	0.723±0.004 [2.3e-13*]
PrimateAI	0.849±0.004 [7.5e-09*]	PhastCons(•)	0.699±0.004 [4.0e-12*]
MPC	0.834±0.005 [9.1e-09*]	phyloP(•)	0.679±0.004 [9.6e-13*]
Polyphen2_HDIV	0.831±0.004 [1.3e-10*]	FATHMM(•)	0.671±0.004 [1.4e-12*]
MutationAssessor	0.828±0.003 [6.3e-11*]	MutationTaster(•)	0.658±0.006 [5.0e-12*]
M-CAP	0.826±0.004 [1.2e-09*]		

Figure S13: Comparing ROC performance of VARITY_R with other predictors for a high-quality 'core' variant set (MAF < 0.5%). We compare ROC performance for VARITY_R (using nested cross-validation) with other 23 variant pathogenicity predictors. For compactness, a predictor with AUROC smaller than 0.6 (see Table S11) is not shown. Predictors designed specifically for nucleotide variants indicated with a '(•)'. The test set was 9,719 variants (5,912 positive and 3,807 negative examples) from the core training set, after removing variants annotated by HGMD[6] and retaining only variants that had been scored by all methods. At any given false positive rate, true positive rate is averaged over all 10 outer-loop folds and the standard error is indicated by the surrounding grey region. As overall performance measure, AUROC and their standard errors are shown. Statistical significance of performance relative to VARITY_R used a one-sided paired t-test with 9 degrees of freedom (P-values are in brackets, with '*' indicating $P < 0.05$). Other test statistics such as 95% confidence interval and effect size are in Table S11.

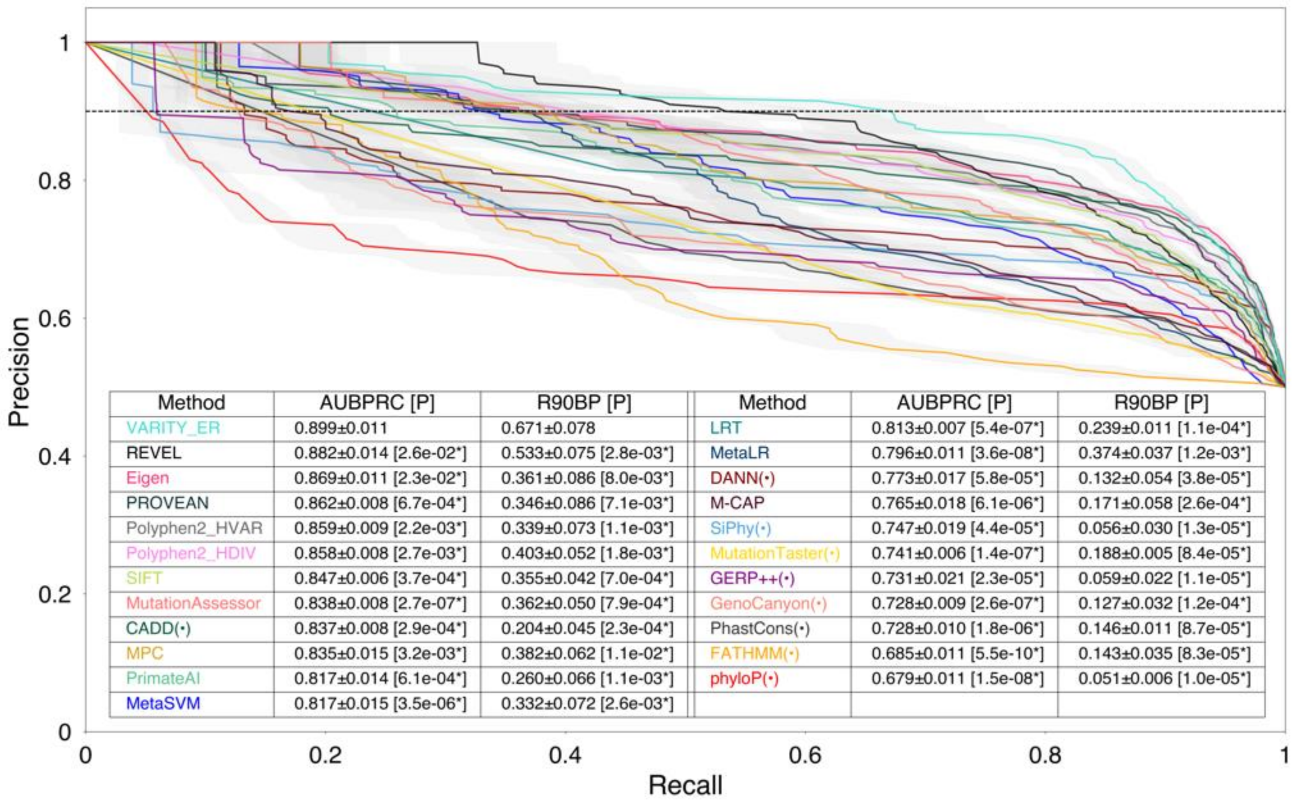

Method	AUBPRC [P]	R90BP [P]	Method	AUBPRC [P]	R90BP [P]
VARITY_ER	0.899±0.011	0.671±0.078	LRT	0.813±0.007 [5.4e-07*]	0.239±0.011 [1.1e-04*]
REVEL	0.882±0.014 [2.6e-02*]	0.533±0.075 [2.8e-03*]	MetaLR	0.796±0.011 [3.6e-08*]	0.374±0.037 [1.2e-03*]
Eigen	0.869±0.011 [2.3e-02*]	0.361±0.086 [8.0e-03*]	DANN(•)	0.773±0.017 [5.8e-05*]	0.132±0.054 [3.8e-05*]
PROVEAN	0.862±0.008 [6.7e-04*]	0.346±0.086 [7.1e-03*]	M-CAP	0.765±0.018 [6.1e-06*]	0.171±0.058 [2.6e-04*]
Polyphen2_HVAR	0.859±0.009 [2.2e-03*]	0.339±0.073 [1.1e-03*]	SiPhy(•)	0.747±0.019 [4.4e-05*]	0.056±0.030 [1.3e-05*]
Polyphen2_HDIV	0.858±0.008 [2.7e-03*]	0.403±0.052 [1.8e-03*]	MutationTaster(•)	0.741±0.006 [1.4e-07*]	0.188±0.005 [8.4e-05*]
SIFT	0.847±0.006 [3.7e-04*]	0.355±0.042 [7.0e-04*]	GERP++(•)	0.731±0.021 [2.3e-05*]	0.059±0.022 [1.1e-05*]
MutationAssessor	0.838±0.008 [2.7e-07*]	0.362±0.050 [7.9e-04*]	GenoCanyon(•)	0.728±0.009 [2.6e-07*]	0.127±0.032 [1.2e-04*]
CADD(•)	0.837±0.008 [2.9e-04*]	0.204±0.045 [2.3e-04*]	PhastCons(•)	0.728±0.010 [1.8e-06*]	0.146±0.011 [8.7e-05*]
MPC	0.835±0.015 [3.2e-03*]	0.382±0.062 [1.1e-02*]	FATHMM(•)	0.685±0.011 [5.5e-10*]	0.143±0.035 [8.3e-05*]
PrimateAI	0.817±0.014 [6.1e-04*]	0.260±0.066 [1.1e-03*]	phyloP(•)	0.679±0.011 [1.5e-08*]	0.051±0.006 [1.0e-05*]
MetaSVM	0.817±0.015 [3.5e-06*]	0.332±0.072 [2.6e-03*]			

Figure S14: Comparing balanced precision recall performance of VARITY_ER with other predictors in predicting a high-quality 'core' variant set (MAF < 10^{-6}). We compare balanced precision recall performance for VARITY_ER (using nested cross-validation) with other 23 variant pathogenicity predictors. For compactness, one predictor with AUBPRC < 0.6 is not shown (see Table S13). Predictors designed specifically for nucleotide variants indicated with a '(•)'. The test set was 5,160 variants (4,675 positive and 485 negative examples) from the core training set, after removing variants annotated by HGMD[6] and retaining only variants that had been scored by all methods. Recall was averaged over all 10 outer-loop folds and the standard error is indicated by the surrounding grey region. As overall performance measures, AUBPRC and R90BP (the black dotted line) and their standard errors are shown. Statistical significance of performance relative to VARITY_ER used a one-sided paired t-test with 9 degrees of freedom (P-values in brackets were indicated with a '*' where P < 0.05). Other test statistics such as 95% confidence interval and effect size are in Table S13.

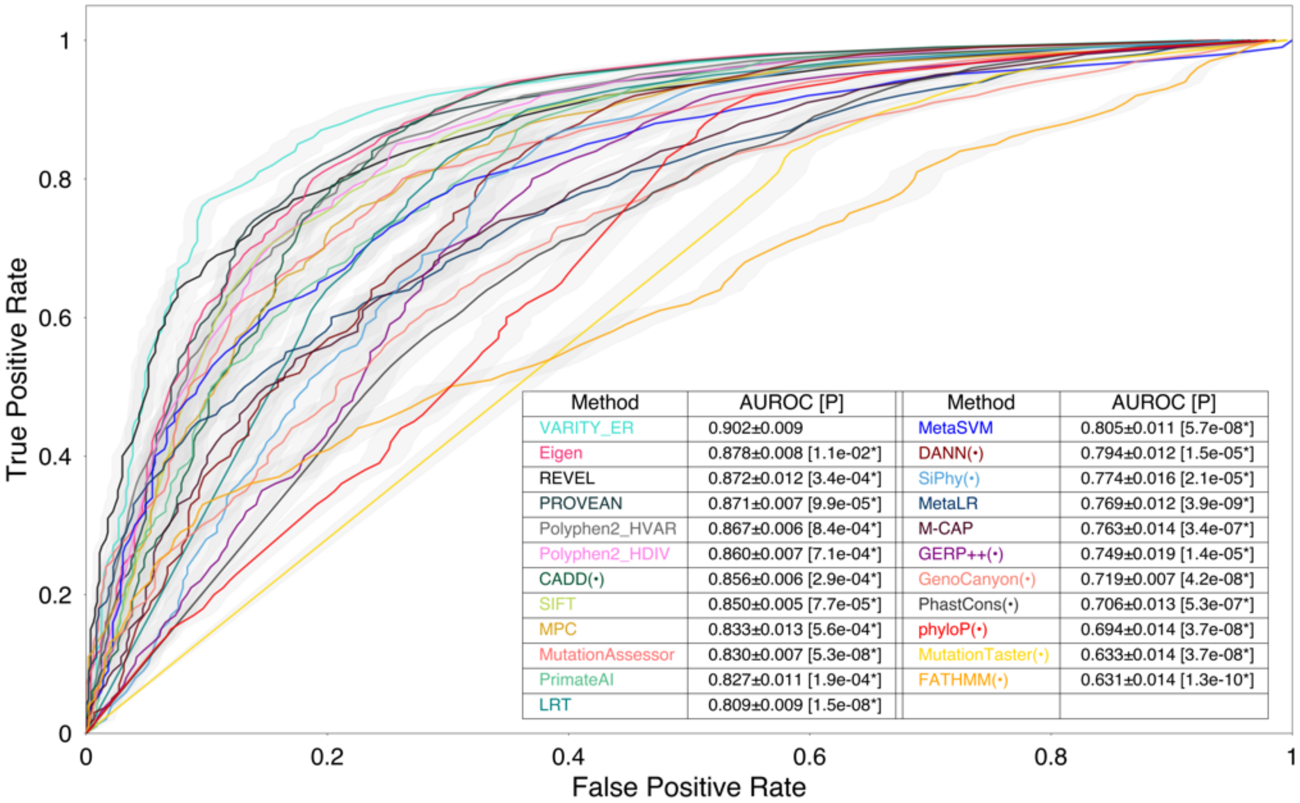

Figure S15: Comparing ROC performance of VARITY_ER with other predictors in predicting a high-quality 'core' variant set (MAF < 10^{-6}). We compare ROC performance for VARITY_ER (using nested cross-validation) with other 23 variant pathogenicity predictors. For compactness, one predictor with AUROC < 0.6 is not shown (see Table S14). Predictors designed specifically for nucleotide variants indicated with a '(•)'. The test set was 5,160 variants (4,675 positive and 485 negative examples from the core training set, after removing variants annotated by HGMD[6] and retaining only variants that had been scored by all methods. At any given false positive rate, true positive rate is averaged over all 10 outer-loop folds and the standard error is indicated by the surrounding grey region. As overall performance measure, AUROC and their standard errors are shown. Statistical significance of performance relative to VARITY_R used a one-sided paired t-test with 9 degrees of freedom (P-values in brackets were indicated with a '*' where P < 0.05). Other test statistics such as 95% confidence interval and effect size are in Table S14.

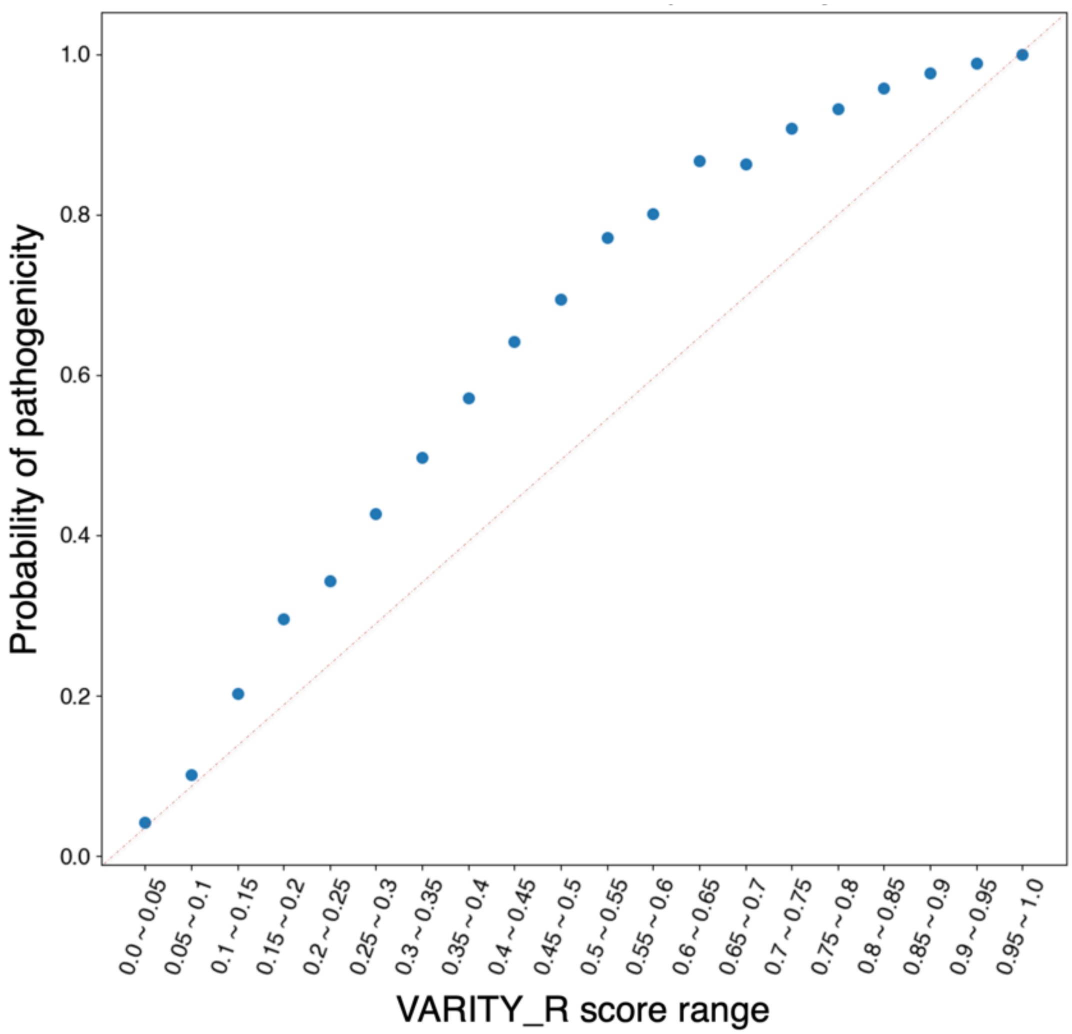

Figure S16: The relationship between VARITY_R score and probability of pathogenicity. VARITY_R scores on the 38,047 labelled core set variants (MAF < 0.5%) were used for the plot. All core set variants were assorted into 20 bins each represents a different VARITY score range (see X axis). For each bin, the probability of pathogenicity (Y axis; fraction of variants annotated as either likely pathogenic or pathogenic in ClinVar) was calculated as number of variants labelled as ‘putatively pathogenic’ divided by number of total variants in the bin.

Supplemental Tables

Table S1: Features used by VARITY models

Feature Group	Feature Names	Feature Description	Source
Conservation Scores	PROVEAN_selected_score	Provean[7] score	dbNSFP (V4.0b2)[17]
	SIFT_selected_score	SIFT[8-9] score	
	Evm_selected_score	EVMutation[10] score	
	Integrated_fitCons_score	fitCons[11] score	
	LRT_score	LRT[12] score	
	GERP++_RS	GERP++[13] score	
	phyloP30way_mammalian	phyloP[14] score	
	phastCons30way_mammalian	phastCons[15] score	
	SiPhy_29way_logOdds	SiPhy[16] score	
AA Delta properties	mw_delta	Change in molecular weight	"*Handbook of Chemistry and Physics*"[18]
	pka_delta	Change in acid dissociation constant pK_a	
	pkb_delta	Change in base dissociation constant pK_b	
	pi_delta	Change in isoelectric point pI	
	hi_delta	Change in hydropathy index	Kyte, J. & Doolittle, R. F.[19]
	pbr_delta	Change in percent buried residues	*"Proteins and proteomics: A laboratory manual"*[20]
	avbr_delta	Change in average buried residue volume	
	vadw_delta	Change in Van der Waals volume	
	asa_delta	Change in side chain accessible surface area	
	cyclic_delta	Change to/from cyclic amino acid (proline)	*"Biochemistry"*[21]
	positve_delta	Change to/from positive-charge amino acid	
	negative_delta	Change to/from negative-charge amino acid	
	charge_delta	Change to/from charged amino acid	
	Hydrophobic_delta	Change to/from hydrophobic amino acid	
	polar_delta	Change to/from polar amino acid	
	aromatic_delta	Change to/from aromatic amino acid	
	aliphatic_delta	Change to/from aliphatic amino acid	
	size_delta	Change in size	
	ionizable_delta	Change to/from ionizable amino acid	
	hbond_delta	Change to/from hydrogen-bonding amino acid	
	sulfur_delta	Change to/from sulfur-containing amino acid	
	essential_delta	Change to/from human-essential amino acid	
Secondary Structure	aa_psipred_H	Alpha Helix	PSIPRED[22]
	aa_psipred_E	Beta Sheet	
	aa_psipred_C	Coiled Coil	
Accessible Surface Area	asa_mean	Accessible Surface Area	PDBePISA[23]
Protein-protein interaction	bsa_max	Maximum solvent-accessible surface area buried by interaction partners	
	solv_ne_abs_max	Maximum solvation energy change by interaction partners	
	h_bond_max	Maximum # of hydrogen bonds with interaction partners	
	salt_bridge_max	Maximum # of salt bridges with interaction partners	
	covelent_bond_max	Maximum # of covalent bonds with interaction partners	
	disulfide_bond_max	Maximum # of disulfide bonds with interaction partners	
BLOSUM	blosum100	Blosum100	Henikoff, S. & Henikoff, J. G.[24]

In/Out Domain	in_domain	Located in vs. out of Pfam Domain	Pfam [25]

Table S2: Multiplexed Assays of Variant Effect (MAVE) variants included in training

MAVE Proteins	Uniprot ID	MIM ID	Number of Variants	Source
UBE2I	P63279	601661	2,879	Weile et al[26]
SUMO1	P63165	601912	1,779	
CALM1	P0DP23	114180	2,525	
TPK1	Q9H3S4	606370	4,124	
NCS1	P62166	603315	2,997	Sun et al., in preparation
GDI1	P31150	300104	4,365	Silverstein et al., in preparation
TECR	Q9NZ01	610057	3,756	Kishore et al., in preparation
MTHFR	P42898	607093	11,737	Weile et al[27]
CBS	P35520	613381	8,354	Sun, S. et al[28]
BRCA1	P38398	113705	1,837	Findlay, G. M. et al[29]
PTEN	P60484	601728	4,112	Matreyek, K. A. et al[30]
TPMT	P51580	187680	3,689	

Table S3: Core and add-on training sets, informative properties and hyperparameters

Resources	Training sets	# of Variants	VARITY_R	VARITY_ER	Informative Properties	Hyperparameters
ClinVAR[1]	Extremely Rare Positive (AF <10^{-6})	17,936	Core	Core	Allele frequency and ClinVAR review stars	L, k, x_0 parameters from each logistic function used for weighting are optimized for each pairing of a training set and informative property.
	Extremely Rare Negative (AF <10^{-6})	856	Core	Core		
	Moderately Rare Positive (0.5% > AF >= 10^{-6})	6,800	Core	Add-on		
	Common Positive (AF >= 0.5%)	30	Add-on			
	Moderately Rare Negative (0.5% > AF >= 10^{-6})	8,495	Core	Add-on		
	Common Negative (AF >= 0.5%)	3,864	Add-on	Add-on		
HGMD[6] (2015 version)	HGMD Positive	40,066	Add-on	Add-on	Allele frequency	
HumsaVar[2]	HumsaVar Positive	2,042	Add-on	Add-on		
	HumsaVar Common Negative (AF >= 0.5%)	1,546	Add-on	Add-on		
	HumsaVar Rare Negative (AF < 0.5%)	4,381	Add-on	Add-on		
gnomAD[3]	gnomAD Common Negative (AF >= 0.5%)	1,480	Add-on	Add-on	Allele frequency and Number of homozygotes	
	gnomAD Rare Negative (AF < 0.5%)	18,579	Add-on	Add-on		
MAVE[26-30]	MAVE Positive	20,147	Add-on	Add-on	Label confidence and Mutational accessibility	
	MAVE Negative	31,486	Add-on	Add-on		

Table S4: Algorithm level hyperparameters used in VARITY

Name	Description
n_estimators	Number of trees in the model
eta (learning rate)	Step size shrinkage used in each boosting step to prevent overfitting
gamma	Minimum loss reduction required to further partition a leaf node
max_depth	Maximum depth of a tree
min_child_weight	Minimum sum of instance weight (Hessian) needed in a child node
subsample	Subsample ratio of the training instances when constructing each tree
colsample_bytree	Subsample ratio of columns(features) when constructing each tree

Table S5: Performance on *de novo* variants from neurodevelopmental disorder studies

Test set: 215 *de novo* variants (188 positively and 27 negatively labelled examples)<br>Statistical test: one sided Z test based on 2,000 bootstrapped test sets										
**Methods**	**AUBPRC**					**R90BP**				
	**Value**	**SE**	**Effect Size**	**95% CI (one sided)**	**P Value (one sided)**	**Value**	**SE**	**Effect Size**	**95% CI (one sided)**	**P Value (one sided)**
VARITY_ER	0.812	0.029				0.476	0.105			
VARITY_R	0.794	0.040	0.019	-0.005 ~ inf	1.22E-01	0.398	0.183	0.077	-0.021 ~ inf	1.90E-01
MPC[31]	0.767	0.041	0.045	-0.017 ~ inf	1.25E-01	0.366	0.165	0.109	-0.101 ~ inf	2.69E-01
MutationAssessor[32]	0.764	0.038	0.048	0.005 ~ inf	2.85E-02	0.370	0.131	0.106	-0.016 ~ inf	7.36E-02
M-CAP[33]	0.764	0.044	0.049	-0.012 ~ inf	9.85E-02	0.261	0.127	0.214	0.026 ~ inf	3.64E-02
PrimateAI[34]	0.749	0.044	0.063	0.015 ~ inf	1.81E-02	0.254	0.166	0.221	0.032 ~ inf	2.13E-02
REVEL[35]	0.747	0.040	0.065	0.010 ~ inf	2.34E-02	0.281	0.116	0.195	0.011 ~ inf	3.40E-02
Provean[7]	0.746	0.047	0.066	0.020 ~ inf	1.26E-02	0.288	0.208	0.188	-0.043 ~ inf	1.01E-01
CADD[36]	0.746	0.041	0.066	0.013 ~ inf	1.71E-02	0.289	0.138	0.187	0.016 ~ inf	4.34E-02
Polyphen2_HVAR[37-38]	0.741	0.040	0.071	0.003 ~ inf	4.52E-02	0.300	0.129	0.176	-0.112 ~ inf	1.41E-01
SIFT[8-9]	0.729	0.046	0.083	0.030 ~ inf	3.60E-03	0.157	0.138	0.319	0.033 ~ inf	6.35E-03
Eigen[39]	0.700	0.046	0.112	0.055 ~ inf	6.15E-04	0.197	0.137	0.279	0.080 ~ inf	1.03E-02
MetaLR[40]	0.699	0.040	0.113	0.043 ~ inf	3.86E-03	0.211	0.057	0.265	0.090 ~ inf	7.75E-03
MutationTaster[41]	0.697	0.039	0.116	0.068 ~ inf	7.11E-04	0.152	0.037	0.323	0.151 ~ inf	8.07E-04
Polyphen2_HDIV[37-38]	0.692	0.046	0.120	0.036 ~ inf	6.69E-03	0.145	0.094	0.331	0.054 ~ inf	9.71E-03
LRT[12]	0.685	0.038	0.127	0.072 ~ inf	2.58E-04	0.140	0.035	0.335	0.165 ~ inf	4.78E-04
MetaSVM[40]	0.680	0.043	0.132	0.063 ~ inf	8.48E-04	0.162	0.075	0.313	0.143 ~ inf	1.77E-03
FATHMM[41]	0.676	0.038	0.136	0.063 ~ inf	1.58E-03	0.202	0.056	0.273	0.085 ~ inf	8.55E-03
GenoCanyon[42]	0.647	0.034	0.165	0.093 ~ inf	3.63E-05	0.148	0.066	0.328	0.128 ~ inf	3.31E-03
GERP++[13]	0.636	0.046	0.176	0.073 ~ inf	1.18E-03	0.071	0.050	0.405	0.206 ~ inf	3.29E-04
phastCons[15]	0.626	0.052	0.187	0.082 ~ inf	1.17E-03	0.050	0.061	0.426	0.222 ~ inf	2.10E-04
SiPhy[16]	0.610	0.035	0.203	0.124 ~ inf	4.71E-06	0.044	0.060	0.432	0.228 ~ inf	2.36E-04
DANN[44]	0.596	0.031	0.217	0.136 ~ inf	2.76E-07	0.028	0.036	0.448	0.255 ~ inf	3.72E-05
phyloP[14]	0.593	0.037	0.220	0.127 ~ inf	3.97E-06	0.036	0.032	0.440	0.261 ~ inf	3.70E-05
fitCons[11]	0.590	0.041	0.222	0.135 ~ inf	1.67E-06	0.035	0.045	0.440	0.260 ~ inf	3.36E-05

Table S6: ROC performance on *de novo* variants in neurodevelopmental disorder studies

Test set: 215 *de novo* variants (188 positively and 27 negatively labelled examples)<br>Statistical test: one sided Z test based on 2,000 bootstrapped test set					
**Methods**	**AUROC**				
	**Value**	**SE**	**Effect Size**	**95% CI (one sided)**	**P Value (one sided)**
VARITY_ER	0.737	0.038			
VARITY_R	0.730	0.042	0.007	-0.014 ~ inf	2.79E-01
M-CAP[33]	0.717	0.048	0.020	-0.050 ~ inf	3.28E-01
MutationAssessor[32]	0.697	0.046	0.040	-0.020 ~ inf	1.32E-01
MPC[31]	0.694	0.046	0.043	-0.025 ~ inf	1.53E-01
PrimateAI[34]	0.685	0.048	0.052	-0.006 ~ inf	7.28E-02
CADD[36]	0.675	0.047	0.062	-0.000 ~ inf	4.59E-02
REVEL[35]	0.673	0.045	0.064	-0.001 ~ inf	4.91E-02
Polyphen2_HVAR[37-38]	0.673	0.048	0.064	-0.006 ~ inf	6.69E-02
SIFT[8-9]	0.673	0.052	0.064	0.002 ~ inf	4.15E-02
Provean[7]	0.668	0.045	0.069	0.028 ~ inf	2.87E-03
Polyphen2_HDIV[37-38]	0.644	0.053	0.093	0.011 ~ inf	3.30E-02
MetaLR[40]	0.637	0.054	0.100	0.003 ~ inf	4.21E-02
Eigen[39]	0.629	0.050	0.108	0.047 ~ inf	2.02E-03
MetaSVM[40]	0.609	0.051	0.129	0.040 ~ inf	8.87E-03
FATHMM[41]	0.599	0.051	0.139	0.035 ~ inf	1.51E-02
GERP++[13]	0.593	0.054	0.144	0.020 ~ inf	2.32E-02
LRT[12]	0.585	0.050	0.152	0.075 ~ inf	4.12E-04
MutationTaster[41]	0.585	0.048	0.152	0.085 ~ inf	8.75E-05
phastCons[15]	0.576	0.045	0.161	0.057 ~ inf	5.19E-03
phyloP[14]	0.564	0.046	0.173	0.057 ~ inf	2.79E-03
fitCons[11]	0.557	0.042	0.181	0.085 ~ inf	6.09E-04
GenoCanyon[42]	0.555	0.039	0.182	0.103 ~ inf	5.66E-05
DANN[44]	0.553	0.040	0.184	0.085 ~ inf	2.90E-04
SiPhy[16]	0.548	0.036	0.189	0.097 ~ inf	1.35E-04

Table S7: Comparing VARITY_ER with MPC[31] and M_CAP[45] Individually on *de novo* variants in neurodevelopmental disorder studies

Statistical test: one sided Z test based on 2,000 bootstrapped test set							
**Methods**	**Number of variants**	**Metrics**	**VARITY_ER**	**Value**	**Effect Size**	**95% CI**	**P Value**
MPC[31]	323<br>[P:285, N:38]	AUBPRC	0.791±0.025	0.731±0.040	0.059	0.000 ~ inf	4.79E-02
		R90BP	0.428±0.086	0.209±0.156	0.219	-0.067 ~ inf	9.43E-02
		AUROC	0.787±0.026	0.712±0.047	0.075	0.004 ~ inf	4.30E-02
M-CAP[33]	365<br>[P:320, N:45]	AUBPRC	0.765±0.025	0.709±0.035	0.056	0.007 ~ inf	2.91E-02
		R90BP	0.366±0.085	0.167±0.052	0.198	0.069 ~ inf	1.20E-02
		AUROC	0.691±0.033	0.674±0.042	0.017	-0.036 ~ inf	3.00E-01

Table S8: Pearson correlation between predictor scores and variant effect map scores

Pearson Correlation Coefficient Performance on 6 variant effect maps<br>Statistical test: one-sided paired t-test in 6 variant effect maps											
	BRCA1	***CALM1***	***CBS***	***PTEN***	***TPK1***	***VKORC1***	**PCC Mean**	**SE**	**Effect Size**	**95% CI (one sided)**	**P Value (one sided)**
VARITY_ER_LOO	0.570	0.211	0.475	0.493	0.199	0.485	0.405	0.065			
VARITY_ER	0.583	0.149	0.481	0.496	0.206	0.485	0.400	0.072	0.005	-0.016 ~ inf	3.29E-01
VARITY_R	0.554	0.123	0.444	0.444	0.226	0.471	0.377	0.067	0.028	-0.001 ~ inf	6.67E-02
VARITY_R_LOO	0.532	0.205	0.424	0.435	0.178	0.470	0.374	0.060	0.031	0.016 ~ inf	7.25E-03
REVEL[35]	0.595	0.134	0.411	0.417	0.188	0.450	0.366	0.071	0.040	0.009 ~ inf	3.14E-02
Eigen[39]	0.438	0.212	0.344	0.337	0.189	0.395	0.319	0.040	0.086	0.036 ~ inf	1.29E-02
Provean[7]	0.376	0.077	0.403	0.430	0.184	0.397	0.311	0.059	0.094	0.048 ~ inf	6.94E-03
MPC[31]	0.294	0.222	0.455	0.284	0.165	0.418	0.306	0.046	0.099	0.012 ~ inf	4.54E-02
CADD[36]	0.384	0.189	0.338	0.325	0.166	0.422	0.304	0.042	0.101	0.048 ~ inf	8.89E-03
M-CAP[33]	0.532	0.049	0.419	0.279	0.189	0.342	0.302	0.070	0.104	0.043 ~ inf	1.27E-02
MetaSVM[40]	0.453	0.131	0.287	0.244	0.218	0.435	0.295	0.052	0.111	0.038 ~ inf	1.89E-02
MetaLR[40]	0.436	0.111	0.250	0.219	0.221	0.425	0.277	0.052	0.128	0.047 ~ inf	1.68E-02
PrimateAI[34]	0.359	0.057	0.346	0.261	0.101	0.240	0.227	0.051	0.178	0.133 ~ inf	3.87E-04
SiPhy[16]	0.359	0.108	0.188	0.036	0.103	0.236	0.172	0.047	0.234	0.133 ~ inf	3.93E-03
GERP++[13]	0.290	0.123	0.168	0.054	0.125	0.257	0.170	0.036	0.236	0.131 ~ inf	4.42E-03
DANN[44]	0.165	0.068	0.175	0.131	0.070	0.290	0.150	0.034	0.255	0.168 ~ inf	1.51E-03
FATHMM[41]	0.358	0.030	0.157	0.003	0.114	0.188	0.142	0.052	0.264	0.159 ~ inf	2.81E-03
phyloP[14]	0.269	0.060	0.166	0.019	0.063	0.235	0.135	0.042	0.270	0.177 ~ inf	1.56E-03
LRT[12]	0.222	0.044	0.159	0.067	0.067	0.224	0.131	0.033	0.275	0.191 ~ inf	9.05E-04
GenoCanyon[42]	0.188	0.045	0.080	0.066	0.054	0.163	0.099	0.025	0.306	0.215 ~ inf	8.20E-04
phastCons[15]	0.161	0.002	0.155	0.009	0.043	0.139	0.085	0.031	0.320	0.229 ~ inf	6.81E-04
fitCons[11]	0.017	0.136	0.072	0.136	0.042	0.064	0.078	0.020	0.328	0.194 ~ inf	3.21E-03
MutationTaster[41]	0.073	0.065	0.111	0.047	0.001	0.049	0.058	0.015	0.348	0.240 ~ inf	9.82E-04

Table S9: Spearman correlation between predictor scores and variant effect map scores

Spearman Correlation Coefficient Performance on 6 variant effect maps<br>Statistical test: one-sided paired t-test in 6 variant effect maps											
	BRCA1	***CALM1***	***CBS***	***PTEN***	***TPK1***	***VKORC1***	**SRC Mean**	**SE**	**Effect Size**	**95% CI (one sided)**	**P Value (one sided)**
VARITY_R_LOO	0.470	0.220	0.461	0.425	0.184	0.480	0.373	0.055			
VARITY_R	0.483	0.132	0.474	0.430	0.223	0.481	0.370	0.063	0.003	-0.030 ~ inf	4.40E-01
VARITY_ER	0.473	0.151	0.458	0.435	0.198	0.485	0.367	0.061	0.007	-0.017 ~ inf	3.14E-01
VARITY_ER_LOO	0.460	0.145	0.453	0.437	0.190	0.485	0.362	0.062	0.012	-0.013 ~ inf	2.09E-01
REVEL[35]	0.520	0.121	0.411	0.401	0.182	0.453	0.348	0.065	0.025	-0.012 ~ inf	1.33E-01
Eigen[39]	0.452	0.202	0.365	0.338	0.195	0.421	0.329	0.044	0.045	0.013 ~ inf	2.49E-02
CADD[36]	0.364	0.190	0.351	0.315	0.172	0.445	0.306	0.043	0.067	0.032 ~ inf	8.10E-03
Provean[7]	0.323	0.083	0.389	0.402	0.173	0.450	0.303	0.059	0.070	0.025 ~ inf	1.73E-02
MPC[31]	0.266	0.195	0.435	0.309	0.173	0.418	0.299	0.045	0.074	0.018 ~ inf	2.91E-02
M-CAP[33]	0.493	0.045	0.406	0.277	0.203	0.349	0.295	0.065	0.078	0.013 ~ inf	3.90E-02
MetaSVM[40]	0.434	0.133	0.295	0.219	0.214	0.465	0.293	0.054	0.080	0.012 ~ inf	4.16E-02
MetaLR[40]	0.413	0.113	0.337	0.216	0.215	0.453	0.291	0.054	0.082	0.019 ~ inf	3.05E-02
PrimateAI[34]	0.302	0.026	0.338	0.280	0.103	0.239	0.214	0.050	0.159	0.117 ~ inf	4.76E-04
GERP++[13]	0.320	0.096	0.217	0.088	0.118	0.333	0.195	0.046	0.178	0.105 ~ inf	3.18E-03
LRT[12]	0.340	0.037	0.296	0.127	0.096	0.246	0.190	0.049	0.183	0.127 ~ inf	9.14E-04
DANN[44]	0.221	0.030	0.245	0.186	0.041	0.385	0.185	0.055	0.189	0.144 ~ inf	2.88E-04
GenoCanyon[42]	0.281	0.035	0.257	0.128	0.074	0.242	0.170	0.043	0.204	0.157 ~ inf	2.39E-04
SiPhy[16]	0.329	0.086	0.154	0.023	0.098	0.287	0.163	0.049	0.211	0.120 ~ inf	3.89E-03
FATHMM[41]	0.234	0.017	0.162	0.022	0.065	0.385	0.147	0.059	0.226	0.140 ~ inf	2.37E-03
phyloP[14]	0.243	0.041	0.195	0.049	0.039	0.281	0.141	0.045	0.232	0.171 ~ inf	4.67E-04
fitCons[11]	0.032	0.094	0.038	0.264	0.083	0.105	0.103	0.034	0.270	0.152 ~ inf	4.22E-03
phastCons[15]	0.182	0.036	0.147	0.058	0.014	0.143	0.097	0.028	0.277	0.216 ~ inf	2.05E-04
MutationTaster[41]	0.004	0.051	0.156	0.204	0.029	0.034	0.079	0.033	0.294	0.191 ~ inf	1.64E-03

Table S10: VARITY_R Performance (AUBPRC) comparison with 23 other predictors

Performance on core set (ClinVAR rare variants) using 10-fold nested cross validation based on 9,719 variants (5,912 positive and 3,807 negative examples; variants labelled by HGMD[6] were removed)
Statistical test: one-sided paired t-test on 10 outer-loop test sets relative to VARITY_R (9 degrees of freedom)

Methods	AUBPRC					R90BP				
	**Value**	**SE**	**Effect Size**	**95% CI (one sided)**	**P Value (one sided)**	**Value**	**SE**	**Effect Size**	**95% CI (one sided)**	**P Value (one sided)**
VARITY_R	0.920	0.003				0.752	0.012			
REVEL[35]	0.893	0.002	0.027	0.022 ~ inf	3.44E-06	0.623	0.012	0.129	0.103 ~ inf	5.64E-06
Eigen[39]	0.871	0.003	0.049	0.044 ~ inf	2.23E-08	0.494	0.024	0.258	0.218 ~ inf	7.14E-07
Provean[7]	0.862	0.005	0.058	0.050 ~ inf	1.06E-07	0.442	0.050	0.310	0.234 ~ inf	2.91E-05
SIFT[8-9]	0.853	0.002	0.067	0.062 ~ inf	2.64E-09	0.411	0.016	0.341	0.300 ~ inf	6.60E-08
PrimateAI[34]	0.846	0.005	0.074	0.067 ~ inf	7.41E-09	0.419	0.031	0.333	0.285 ~ inf	3.38E-07
Polyphen2_HVAR[3738]	0.844	0.004	0.076	0.072 ~ inf	7.64E-11	0.291	0.038	0.461	0.405 ~ inf	8.14E-08
MPC[31]	0.844	0.005	0.077	0.068 ~ inf	4.17E-08	0.429	0.021	0.323	0.282 ~ inf	1.13E-07
MutationAssessor[32]	0.838	0.003	0.082	0.076 ~ inf	3.57E-10	0.411	0.021	0.341	0.293 ~ inf	3.03E-07
MetaSVM[40]	0.832	0.003	0.088	0.081 ~ inf	1.52E-09	0.442	0.013	0.310	0.283 ~ inf	4.33E-09
CADD[36]	0.830	0.006	0.090	0.081 ~ inf	6.82E-09	0.149	0.049	0.603	0.522 ~ inf	2.06E-07
M-CAP[33]	0.828	0.006	0.092	0.082 ~ inf	3.06E-08	0.354	0.039	0.398	0.323 ~ inf	3.37E-06
Polyphen2_HDIV[3738]	0.827	0.004	0.093	0.087 ~ inf	7.05E-10	0.292	0.016	0.460	0.430 ~ inf	4.24E-10
MetaLR[40]	0.816	0.004	0.104	0.097 ~ inf	1.25E-09	0.384	0.015	0.368	0.337 ~ inf	3.39E-09
LRT[12]	0.807	0.005	0.113	0.106 ~ inf	1.76E-10	0.260	0.012	0.492	0.471 ~ inf	8.10E-12
MutationTaster[41]	0.747	0.003	0.173	0.166 ~ inf	7.56E-12	0.194	0.004	0.558	0.538 ~ inf	1.14E-12
phastCons[15]	0.714	0.003	0.207	0.200 ~ inf	6.12E-13	0.131	0.003	0.620	0.600 ~ inf	8.34E-13
GenoCanyon[42]	0.710	0.005	0.211	0.201 ~ inf	1.01E-11	0.044	0.004	0.708	0.685 ~ inf	5.56E-13
FATHMM[41]	0.702	0.004	0.218	0.209 ~ inf	7.09E-12	0.158	0.025	0.594	0.548 ~ inf	1.86E-09
SiPhy[16]	0.699	0.006	0.221	0.209 ~ inf	5.01E-11	0.009	0.004	0.743	0.722 ~ inf	2.26E-13
DANN[44]	0.695	0.005	0.225	0.216 ~ inf	6.67E-12	0.013	0.005	0.739	0.716 ~ inf	5.39E-13
GERP++[13]	0.675	0.004	0.245	0.239 ~ inf	4.69E-14	0.009	0.006	0.743	0.725 ~ inf	4.69E-14
phyloP[14]	0.650	0.004	0.270	0.263 ~ inf	1.35E-13	0.039	0.003	0.713	0.694 ~ inf	1.25E-13
fitCons[11]	0.532	0.004	0.388	0.378 ~ inf	9.66E-14	0.001	0.000	0.751	0.730 ~ inf	1.73E-13

Table S11: VARITY_R Performance (AUROC) comparison with 23 other predictors

Performance on core set (ClinVAR rare variants) using 10-fold nested cross validation based on 9,719 variants (5,912 positive 3,807 negative examples, variants labelled only by HGMD[6] were removed)
Statistical test: one-sided paired t-test on 10 outer-loop test sets relative to VARITY_R (9 degrees of freedom)

Methods	AUROC				
	Value	SE	Effect Size	95% CI (one sided)	P Value (one sided)
VARITY_R	0.919	0.002			
REVEL[35]	0.888	0.002	0.032	0.027 ~ inf	2.41E-07
Eigen[39]	0.877	0.003	0.042	0.038 ~ inf	1.76E-08
Provean[7]	0.87	0.004	0.049	0.044 ~ inf	3.02E-08
CADD[36]	0.86	0.004	0.06	0.054 ~ inf	4.42E-09
Polyphen2_HVAR[37-38]	0.858	0.003	0.062	0.058 ~ inf	2.49E-10
SIFT[8-9]	0.852	0.003	0.068	0.063 ~ inf	3.93E-10
PrimateAI[34]	0.849	0.004	0.071	0.064 ~ inf	7.45E-09
MPC[31]	0.834	0.005	0.085	0.077 ~ inf	9.06E-09
Polyphen2_HDIV[37-38]	0.831	0.004	0.089	0.084 ~ inf	1.31E-10
MutationAssessor[32]	0.828	0.003	0.092	0.087 ~ inf	6.28E-11
MetaSVM[40]	0.826	0.003	0.094	0.089 ~ inf	4.40E-11
M-CAP[33]	0.826	0.004	0.093	0.086 ~ inf	1.20E-09
MetaLR[40]	0.801	0.004	0.119	0.112 ~ inf	1.17E-10
LRT[12]	0.801	0.005	0.118	0.111 ~ inf	2.54E-10
SiPhy[16]	0.751	0.004	0.168	0.159 ~ inf	7.51E-11
DANN[44]	0.749	0.005	0.171	0.162 ~ inf	2.15E-11
GenoCanyon[42]	0.726	0.005	0.194	0.185 ~ inf	1.37E-11
GERP++[13]	0.723	0.004	0.197	0.191 ~ inf	2.27E-13
phastCons[15]	0.699	0.004	0.221	0.212 ~ inf	4.03E-12
phyloP[14]	0.679	0.004	0.24	0.232 ~ inf	9.63E-13
FATHMM[41]	0.671	0.004	0.248	0.239 ~ inf	1.40E-12
MutationTaster[41]	0.658	0.006	0.262	0.251 ~ inf	4.98E-12
fitCons[11]	0.531	0.006	0.389	0.377 ~ inf	3.10E-13

Table S12: Comparing VARITY_R performance with EVMutation[10] and DeepSequence[45]

Methods	Number of variants	Metrics	VARITY_R	Value	Effect Size	95% CI	P Value
EVMutation[10]	6,121 [P:4,517, N:1,604]	AUBPRC	0.920±0.005	0.859±0.009	0.060	0.050 ~ inf	1.46E-06
		R90BP	0.748±0.021	0.415±0.065	0.333	0.247 ~ inf	4.42E-05
		AUROC	0.921±0.004	0.864±0.007	0.057	0.051 ~ inf	5.89E-08
DeepSeqeuence[45]	200 [P:153, N:47]	AUBPRC	0.996±0.002	0.917±0.019	0.078	0.046 ~ inf	1.09E-03
		R90BP	0.982±0.009	0.696±0.088	0.286	0.144 ~ inf	3.26E-03
		AUROC	0.994±0.003	0.877±0.031	0.117	0.064 ~ inf	2.05E-03

Table S13: VARITY_ER Performance comparison (AUBRPC) with 23 other predictors

Performance on core set (ClinVAR extremely rare variants) using 10-fold nested cross validation based on 5,160 variants (4,675 positive and 485 negative examples, variants labelled only by HGMD[6] were removed)
Statistical test: one-sided paired t-test on 10 outer-loop test sets relative to VARITY_R (9 degrees of freedom)

Methods	AUBPRC					R90BP				
	Value	SE	Effect Size	95% CI (one sided)	P Value (one sided)	Value	SE	Effect Size	95% CI (one sided)	P Value (one sided)
VARITY_ER	0.899	0.011				0.671	0.078			
REVEL[35]	0.882	0.014	0.017	0.004 ~ inf	2.55E-02	0.533	0.075	0.138	0.071 ~ inf	2.82E-03
Eigen[39]	0.869	0.011	0.030	0.007 ~ inf	2.30E-02	0.361	0.086	0.310	0.128 ~ inf	7.99E-03
Provean[7]	0.862	0.008	0.037	0.023 ~ inf	6.72E-04	0.346	0.086	0.325	0.139 ~ inf	7.10E-03
Polyphen2_HVAR[37-38]	0.859	0.009	0.040	0.021 ~ inf	2.24E-03	0.339	0.073	0.332	0.196 ~ inf	1.08E-03
Polyphen2_HDIV[37-38]	0.858	0.008	0.041	0.021 ~ inf	2.70E-03	0.403	0.052	0.268	0.149 ~ inf	1.78E-03
SIFT[8-9]	0.847	0.006	0.052	0.034 ~ inf	3.73E-04	0.355	0.042	0.316	0.195 ~ inf	7.05E-04
MutationAssessor[32]	0.838	0.008	0.061	0.052 ~ inf	2.69E-07	0.362	0.050	0.309	0.189 ~ inf	7.86E-04
CADD[36]	0.837	0.008	0.062	0.041 ~ inf	2.87E-04	0.204	0.045	0.467	0.315 ~ inf	2.30E-04
MPC[31]	0.835	0.015	0.064	0.032 ~ inf	3.23E-03	0.382	0.062	0.289	0.108 ~ inf	1.08E-02
PrimateAI[34]	0.817	0.014	0.082	0.051 ~ inf	6.14E-04	0.260	0.066	0.411	0.242 ~ inf	1.12E-03
MetaSVM[40]	0.817	0.015	0.082	0.067 ~ inf	3.53E-06	0.332	0.072	0.339	0.178 ~ inf	2.61E-03
LRT[12]	0.813	0.007	0.086	0.073 ~ inf	5.39E-07	0.239	0.011	0.432	0.305 ~ inf	1.09E-04
MetaLR[40]	0.796	0.011	0.103	0.092 ~ inf	3.62E-08	0.374	0.037	0.297	0.173 ~ inf	1.23E-03
DANN[44]	0.773	0.017	0.126	0.092 ~ inf	5.81E-05	0.132	0.054	0.539	0.402 ~ inf	3.78E-05
M-CAP[33]	0.765	0.018	0.134	0.107 ~ inf	6.10E-06	0.171	0.058	0.500	0.335 ~ inf	2.62E-04
SiPhy[16]	0.747	0.019	0.152	0.113 ~ inf	4.36E-05	0.056	0.030	0.615	0.479 ~ inf	1.26E-05
MutationTaster[41]	0.741	0.006	0.158	0.137 ~ inf	1.40E-07	0.188	0.005	0.483	0.347 ~ inf	8.42E-05
GERP++[13]	0.731	0.021	0.168	0.128 ~ inf	2.34E-05	0.059	0.022	0.612	0.478 ~ inf	1.13E-05
GenoCanyon[42]	0.728	0.009	0.170	0.147 ~ inf	2.59E-07	0.127	0.032	0.544	0.383 ~ inf	1.19E-04
phastCons[15]	0.728	0.010	0.171	0.142 ~ inf	1.75E-06	0.146	0.011	0.525	0.376 ~ inf	8.68E-05
FATHMM[41]	0.685	0.011	0.214	0.199 ~ inf	5.54E-10	0.143	0.035	0.528	0.379 ~ inf	8.29E-05
phyloP[14]	0.679	0.011	0.220	0.198 ~ inf	1.48E-08	0.051	0.006	0.620	0.487 ~ inf	9.96E-06
fitCons[11]	0.563	0.007	0.336	0.317 ~ inf	1.10E-10	0.027	0.012	0.644	0.511 ~ inf	7.42E-06

Table S14: VARITY_ER Performance (AUROC) comparison with 23 other predictors

Performance on core set (ClinVAR extremely rare variants) using 10-fold nested cross validation based on 5,160 variants (4,675 positive and 485 negative examples, variants labelled only by HGMD[6] were removed) Statistical test: one-sided paired t-test on 10 outer-loop test sets relative to VARITY_R (9 degrees of freedom)					
Methods	AUROC				
	Value	SE	Effect Size	95% CI (one sided)	P Value (one sided)
VARITY_ER	0.902	0.009			
Eigen[39]	0.878	0.008	0.024	0.009 ~ inf	1.10E-02
REVEL[35]	0.872	0.012	0.030	0.020 ~ inf	3.41E-04
Provean[7]	0.871	0.007	0.031	0.022 ~ inf	9.89E-05
Polyphen2_HVAR[37-38]	0.867	0.006	0.035	0.021 ~ inf	8.43E-04
Polyphen2_HDIV[3738]	0.860	0.007	0.042	0.026 ~ inf	7.12E-04
CADD[36]	0.856	0.006	0.047	0.031 ~ inf	2.91E-04
SIFT[8-9]	0.850	0.005	0.052	0.038 ~ inf	7.71E-05
MPC[31]	0.833	0.013	0.069	0.043 ~ inf	5.64E-04
MutationAssessor[32]	0.830	0.007	0.072	0.064 ~ inf	5.27E-08
PrimateAI[34]	0.827	0.011	0.075	0.051 ~ inf	1.94E-04
LRT[12]	0.809	0.009	0.093	0.084 ~ inf	1.52E-08
MetaSVM[40]	0.805	0.011	0.097	0.086 ~ inf	5.72E-08
DANN[44]	0.794	0.012	0.108	0.083 ~ inf	1.51E-05
SiPhy[16]	0.774	0.016	0.128	0.098 ~ inf	2.08E-05
MetaLR[40]	0.769	0.012	0.134	0.122 ~ inf	3.88E-09
M-CAP[33]	0.763	0.014	0.139	0.119 ~ inf	3.44E-07
GERP++[13]	0.749	0.019	0.153	0.119 ~ inf	1.39E-05
GenoCanyon[42]	0.719	0.007	0.183	0.162 ~ inf	4.19E-08
phastCons[15]	0.706	0.013	0.196	0.166 ~ inf	5.32E-07
phyloP[14]	0.694	0.014	0.208	0.185 ~ inf	3.71E-08
MutationTaster[41]	0.633	0.014	0.270	0.240 ~ inf	3.66E-08
FATHMM[41]	0.631	0.014	0.271	0.256 ~ inf	1.33E-10
fitCons[11]	0.538	0.007	0.364	0.342 ~ inf	1.40E-10

Table S15: Comparing VARITY_ER Performance with EVMutation[10]

	Size	Metrics	VARITY_R	Value	Effect Size	95% CI	P Value
EVMutation[10]	3,993 [P:3,794, N:199]	AUBPRC	0.886±0.017	0.866±0.013	0.020	0.001 ~ inf	5.37E-02
		R90BP	0.449±0.106	0.409±0.072	0.040	-0.117 ~ inf	3.35E-01
		AUROC	0.886±0.016	0.858±0.012	0.027	0.011 ~ inf	7.61E-03

References

1. Landrum, M.J., Lee, J.M., Benson, M., Brown, G.R., Chao, C., Chitipiralla, S., Gu, B., Hart, J., Hoffman, D., Jang, W., et al. (2018). ClinVar: Improving access to variant interpretations and supporting evidence. Nucleic Acids Res. 46, D1062–D1067.
2. The UniProt Consortium (2017). UniProt: the universal protein knowledgebase. Nucleic Acids Res. 45, D158.
3. Karczewski, K.J., Francioli, L.C., Tiao, G., Cummings, B.B., Alföldi, J., Wang, Q., Collins, R.L., Laricchia, K.M., Ganna, A., Birnbaum, D.P., et al. (2020). The mutational constraint spectrum quantified from variation in 141,456 humans. Nature 581, 434–443.
4. Bergstra, J., Bardenet, R., Bengio, Y., and Kégl, B. (2011). Algorithms for Hyper-Parameter Optimization. Adv. Neural Inf. Process. Syst. 24.
5. Bergstra, J., Komer, B., Eliasmith, C., Yamins, D., and Cox, D.D. (2015). Hyperopt: A Python library for model selection and hyperparameter optimization. Comput. Sci. Discov. 8.
6. Stenson, P.D., Mort, M., Ball, E. V., Evans, K., Hayden, M., Heywood, S., Hussain, M., Phillips, A.D., and Cooper, D.N. (2017). The Human Gene Mutation Database: towards a comprehensive repository of inherited mutation data for medical research, genetic diagnosis and next-generation sequencing studies. Hum. Genet. 136, 665–677.
7. Choi, Y., Sims, G.E., Murphy, S., Miller, J.R., and Chan, A.P. (2012). Predicting the Functional Effect of Amino Acid Substitutions and Indels. PLoS One 7.
8. Kumar, P., Henikoff, S. & Ng, P. C. Predicting the effects of coding non-synonymous variants on protein function using the SIFT algorithm. *Nat. Protoc.* **4**, 1073–1082 (2009).
9. Vaser, R., Adusumalli, S., Leng, S.N., Sikic, M., and Ng, P.C. (2016). SIFT missense predictions for genomes. Nat. Protoc. 11, 1–9.
10. Hopf, T.A., Ingraham, J.B., Poelwijk, F.J., Schärfe, C.P.I., Springer, M., Sander, C., and Marks, D.S. (2017). Mutation effects predicted from sequence co-variation. Nat. Biotechnol. 35, 128–135.
11. Gulko, B., Hubisz, M.J., Gronau, I., and Siepel, A. (2015). A method for calculating probabilities of fitness consequences for point mutations across the human genome. Nat. Genet. 47, 276–283.

12. Chun, S., and Fay, J.C. (2009). Identification of deleterious mutations within three human genomes. Genome Res. 19, 1553–1561.

13. Davydov, E. V., Goode, D.L., Sirota, M., Cooper, G.M., Sidow, A., and Batzoglou, S. (2010). Identifying a high fraction of the human genome to be under selective constraint using GERP++. PLoS Comput. Biol. 6,.

14. Pollard, K.S., Hubisz, M.J., Rosenbloom, K.R., and Siepel, A. (2010). Detection of nonneutral substitution rates on mammalian phylogenies. Genome Res. 20, 110–121.

15. Siepel, A., Bejerano, G., Pedersen, J.S., Hinrichs, A.S., Hou, M., Rosenbloom, K., Clawson, H., Spieth, J., Hillier, L.D.W., Richards, S., et al. (2005). Evolutionarily conserved elements in vertebrate, insect, worm, and yeast genomes. Genome Res. 15, 1034–1050.

16. Garber, M., Guttman, M., Clamp, M., Zody, M.C., Friedman, N., and Xie, X. (2009). Identifying novel constrained elements by exploiting biased substitution patterns. Bioinformatics 25, 54–62.

17. Liu, X., Wu, C., Li, C., and Boerwinkle, E. (2016). dbNSFP v3.0: A One-Stop Database of Functional Predictions and Annotations for Human Nonsynonymous and Splice-Site SNVs. Hum. Mutat. 37, 235–241.

18. D.R. Lide. (2006) CRC Handbook of Chemistry and Physics, 86th Edition (Boca Raton, FL: CRC Press)

19. J, K., and RF, D. (1982) A simple method for displaying the hydropathic character of a protein. J. Mol. Biol. 157, 105–132.

20. Simpson, R. J. (2003) Proteins and proteomics: A laboratory manual. (Cold Spring Harbor, NY: Cold Spring Harbor Laboratory Press)

21. Garrett, R. H., & Grishm, C. M. (2010) Biochemistry. (Belmont, CA: Brooks/Cole, Cengage Learning)

22. Buchan, D.W.A., and Jones, D.T. (2019). The PSIPRED Protein Analysis Workbench: 20 years on. Nucleic Acids Res. 47, W402–W407.

23. Krissinel, E., and Henrick, K. (2007). Inference of Macromolecular Assemblies from Crystalline State. J. Mol. Biol. 372, 774–797.

24. Henikoff, S., and Henikoff, J.G. (1992). Amino acid substitution matrices from protein blocks. Proc. Natl. Acad. Sci. U. S. A. 89, 10915–10919.

25. El-Gebali, S., Mistry, J., Bateman, A., Eddy, S.R., Luciani, A., Potter, S.C., Qureshi, M., Richardson, L.J., Salazar, G.A., Smart, A., et al. (2019). The Pfam protein families database in 2019. Nucleic Acids Res. 47, D427–D432.

26. Weile, J., Sun, S., Cote, A.G., Knapp, J., Verby, M., Mellor, J.C., Wu, Y., Pons, C., Wong, C., Lieshout, N., et al. (2017). A framework for exhaustively mapping functional missense variants. Mol. Syst. Biol. 13, 957.

27. Weile, J., Kishore, N., Sun, S., Maaieh, R., Verby, M., Li, R., Fotiadou, I., Kitaygorodsky, J., Wu, Y., Holenstein, A., et al. (2021). Shifting landscapes of human MTHFR missense-variant effects. Am. J. Hum. Genet. 108, 1283–1300.

28. Sun, S., Weile, J., Verby, M., Wu, Y., Wang, Y., Cote, A.G., Fotiadou, I., Kitaygorodsky, J., Vidal, M., Rine, J., et al. (2020). A proactive genotype-to-patient-phenotype map for cystathionine beta-synthase. Genome Med. 12, 1–18.

29. Findlay, G.M., Daza, R.M., Martin, B., Zhang, M.D., Leith, A.P., Gasperini, M., Janizek, J.D., Huang, X., Starita, L.M., and Shendure, J. (2018). Accurate classification of BRCA1 variants with saturation genome editing. Nature 562, 217–222.

30. Matreyek, K.A., Starita, L.M., Stephany, J.J., Martin, B., Chiasson, M.A., Gray, V.E., Kircher, M., Khechaduri, A., Dines, J.N., Hause, R.J., et al. (2018). Multiplex assessment of protein variant abundance by massively parallel sequencing. Nat. Genet. 50, 874–882.

31. Samocha, K., Kosmicki, J., Karczewski, K., O'Donnell-Luria, A., Pierce-Hoffman, E., MacArthur, D., Neale, B., and Daly, M. (2017). Regional missense constraint improves variant deleteriousness prediction. BioRxiv 148353.

32. Choi, Y., Sims, G.E., Murphy, S., Miller, J.R., and Chan, A.P. (2012). Predicting the Functional Effect of Amino Acid Substitutions and Indels. PLoS One 7.

33. Jagadeesh, K.A., Wenger, A.M., Berger, M.J., Guturu, H., Stenson, P.D., Cooper, D.N., Bernstein, J.A., and Bejerano, G. (2016). M-CAP eliminates a majority of variants of uncertain significance in clinical exomes at high sensitivity. Nat. Genet. 48, 1581–1586.

34. Sundaram, L., Gao, H., Padigepati, S.R., McRae, J.F., Li, Y., Kosmicki, J.A., Fritzilas, N., Hakenberg, J., Dutta, A., Shon, J., et al. (2018). Predicting the clinical impact of human mutation with deep neural networks. Nat. Genet. 50, 1161–1170.

35. Ioannidis, N.M., Rothstein, J.H., Pejaver, V., Middha, S., McDonnell, S.K., Baheti, S., Musolf, A., Li, Q., Holzinger, E., Karyadi, D., et al. (2016). REVEL: An Ensemble Method for Predicting the Pathogenicity of Rare Missense Variants. Am. J. Hum. Genet. 99, 877–885.

36. Kircher, M., Witten, D.M., Jain, P., O'roak, B.J., Cooper, G.M., and Shendure, J. (2014). A general framework for estimating the relative pathogenicity of human genetic variants. Nat. Genet. 46, 310–315.

37. Adzhubei, I.A., Schmidt, S., Peshkin, L., Ramensky, V.E., Gerasimova, A., Bork, P., Kondrashov, A.S., and Sunyaev, S.R. (2010). A method and server for predicting damaging missense mutations. Nat. Methods 7, 248–249.

38. Adzhubei, I., Jordan, D.M., and Sunyaev, S.R. (2013). Predicting functional effect of human missense mutations using PolyPhen-2. Curr. Protoc. Hum. Genet. Chapter 7.

39. Ionita-Laza, I., Mccallum, K., Xu, B., and Buxbaum, J.D. (2016). A spectral approach integrating functional genomic annotations for coding and noncoding variants. Nat. Genet. 48, 214–220.

40. Dong, C., Wei, P., Jian, X., Gibbs, R., Boerwinkle, E., Wang, K., and Liu, X. (2015). Comparison and integration of deleteriousness prediction methods for nonsynonymous SNVs in whole exome sequencing studies. Hum. Mol. Genet. 24, 2125–2137.

41. Schwarz, J.M., Cooper, D.N., Schuelke, M., and Seelow, D. (2014). Mutationtaster2: Mutation prediction for the deep-sequencing age. Nat. Methods 11, 361–362.

42. Shihab, H.A., Gough, J., Cooper, D.N., Day, I.N.M., and Gaunt, T.R. (2013). Predicting the functional consequences of cancer-associated amino acid substitutions. Bioinformatics 29, 1504–1510.

43. Lu, Q., Hu, Y., Sun, J., Cheng, Y., Cheung, K.H., and Zhao, H. (2015). A statistical framework to predict functional non-coding regions in the human genome through integrated analysis of annotation data. Sci. Rep. 5, 1–13.

44. Quang, D., Chen, Y., and Xie, X. (2015). DANN: A deep learning approach for annotating the pathogenicity of genetic variants. Bioinformatics 31, 761–763.

45. Riesselman, A.J., Ingraham, J.B., and Marks, D.S. (2018). Deep generative models of genetic variation capture the effects of mutations. Nat. Methods 15, 816–822.
